# Neuropeptide dynamics coordinate layered plasticity mechanisms adapting *Drosophila* circadian behavior to changing environment

**DOI:** 10.1126/sciadv.adt7168

**Published:** 2025-08-29

**Authors:** Abhishek Chatterjee, Joydeep De, Béatrice Martin, Elisabeth Chélot, Ping Zhong, François Rouyer

**Affiliations:** ^1^ Institut des Neurosciences Paris-Saclay, Université Paris-Saclay, CNRS, 91400 Saclay, France.; ^2^ Institute of Ecology and Environmental Sciences of Paris (iEES-*Paris*), INRAE, Sorbonne University, CNRS, IRD, UPEC, Université Paris Cité, 78026 Versailles, France.

## Abstract

The *Drosophila* brain contains distinct circadian oscillators responsible for generating the morning and evening bouts of locomotor activity in light-dark cycles. We lack a mechanistic understanding of how environmental changes reshape the resulting bimodal rest-activity pattern. Here, we uncover a seasonal switch mechanism that remodels the evening bout of activity. Under temperate summer-like conditions, levels of the pigment-dispersing factor (PDF) neuropeptide diminish, triggering a cascade. Lowered PDF receptor (PDFR) signaling disinhibits glycogen synthase kinase 3/SHAGGY to advance the evening output, and in parallel, weakens the siesta-promoting *vc*DN1p-SIF*amide* axis to expand the evening peak. Under these conditions, *vc*DN1p takes over the evening pacemaker role by exerting control over the dorsal lateral oscillator neurons. Our findings elucidate how environment-induced changes in PDFR signaling tip the balanced output of the clock network, aligning daily rhythms with seasonal time. Neuropeptide-driven parallel adjustment of clock circuitry and clock protein functioning likely represents a conserved strategy, enabling animals to adapt their daily behavior to seasonal changes.

## Teaser

Neuropeptide-guided plasticity of the clock neuron network operation enables behavioral adaptation to seasonal changes.

## INTRODUCTION

Nearly every organism has a circadian clock that organizes physiological and behavioral processes according to the time of the day. The working mechanism of eukaryotic clocks is centered on transcriptional feedback loops, with posttranscriptional controls setting the clocks’ period close to 24 hours (*1*). While the period of the external day-night cycles is characteristically invariant, the day-to-night ratio varies seasonally in most places on Earth. The clock-driven rhythms have therefore evolved to manifest a stable period but a labile waveform and phase. In mammals, encoding of daylength relies on the plasticity of the circadian waveform in the mammalian suprachiasmatic nucleus (SCN), but the network changes underlying waveform plasticity remain enigmatic (*2*). The 150 clock neurons of the *Drosophila* brain are particularly suited for network analysis, being organized into functionally and anatomically segregated, variably coupled oscillator nodes (*3*, *4*).

In the fruit fly, the conserved biochemical program of the circadian oscillator, at its core, involves CLOCK (CLK) - CYCLE (CYC)–driven transcription of *period (per)* and *timeless (tim)* genes (*1*). The PERIOD (PER) and TIMELESS (TIM) proteins associate to form complexes that repress CLK-CYC activity. A series of posttranscriptional modifications control the subcellular location, half-life, and transcriptional potency of these protein complexes. Light and temperature inflict state-change of the dynamic clock program, mostly by affecting the stability of PER-TIM (*5*–*7*). Consequently, light and temperature changes synchronize the brain clock with the external day-night cycles (entrainment) (*8*, *9*). The external time cues (zeitgeber) also tune the cross-talk between oscillator nodes and selectively gate the oscillator’s overt output (*10*–*12*). The multiscale effect of light and temperature on the clock guarantees exquisite malleability of the rhythms’ phase and waveform across phylogeny.

The daily rest-activity pattern of *Drosophila* is bimodal (*13*) as it is in humans (*14*, *15*). Dawn-active morning oscillator and dusk-active evening oscillator (EO) neurons in the *Drosophila* brain produce the two daily peaks of locomotor activity in anticipation of day/night transition (*16*–*19*). There is support for the general idea that the circadian clock is instrumental in seasonal time measurement (*20*–*22*). In the rodent SCN, the phase-angles of individual oscillator neurons diverge as the day gets longer, wherein the VIP neuropeptide (vasoactive intestinal peptide) plays a key role in adapting the behavioral activity profile to photoperiodic changes (*23*–*26*).

A similar mechanism implicating interoscillator phase gap was invoked to explain fly’s behavioral response to daylength change (*18*, *27*). Seasonal variation in the daily pattern of light and temperature changes necessitates flexibility in the timing of the two activity peaks—two peaks moving closer or apart depending on the prevailing daylength and temperature, and the morning and evening oscillators do show different responses to light and temperature (*18*, *28*–*30*). *Drosophila*’s activity peaks and their source oscillators, however, have a markedly limited ability to track the dawn and dusk transitions across different photoperiods (*31*, *32*), which challenges the long-standing idea that seasonal time is encoded by photoperiod-dependent shifts in interoscillator phase angle (*33*).

In addition to the phase changes, observations in seminatural conditions revealed a dramatic seasonal change in waveform: In tropical and Mediterranean climate, hot and bright summer days induce an afternoon bout of activity that depends on the TRPA1 heat-activated channel (*34*–*37*). Conversely, waveform plasticity was also observed in subarctic Drosophilids that experience unusually long yet mild summer days (*38*–*41*). Under these polar conditions, the evening peak becomes more pronounced, whereas in the standard laboratory setting characterized by high-amplitude 12-hour:12-hour light-dark (LD) cycles, similar to equinox days, the evening peak is only as strong as the morning one and remains tied to the dusk transition. Notably absent is our understanding of the plasticity mechanisms that enable the monitoring of seasonal changes and their translation into behavioral adjustments, especially under the integrative influence of variations in daylength, light, and temperature across seasons.

The neuropeptide pigment-dispersing factor (PDF), synthesized by morning cells, is involved in developmental and physiological responses to photoperiod (*41*–*44*). Downstream of the clock neuronal network, PDF receptor (PDFR) signaling promotes degradation of the EYES ABSENT phosphatase in insulin-producing cells causing female reproductive dormancy on winter days (*42*, *45*). PDF is also important for the photoperiodic adjustment of the fly’s evening activity peak (*46*–*52*). Nonetheless, the tunable organizational changes that enable clock outputs including PDF to drive seasonal adaptation of the daily rest-activity pattern remain elusive.

In this study, we demonstrate that PDF-mediated communication among clock neurons in the Drosophila brain edits the network organogram. By reducing the neural activity of a locomotor-suppressive output circuit, a larger evening peak is produced on summer days. This tunable balance between locomotor-suppressive and -promotive oscillators, set by neuropeptide dynamics, could allow the multioscillator mammalian clock to adaptively mold behavioral waveform and phase.

## RESULTS

### Summer-like conditions induce a drop of PDF levels in cosmopolitan *Drosophila* species

Wild-type *Drosophila melanogaster* flies start to build evening anticipatory activity 3 to 4 hours before lights-OFF in equinox days of standard laboratory conditions (LD 12:12, 25°C). To test flies in conditions that better correspond to their natural environment in temperate climates, we used long days and increased temperature. Owing to the formation of new leaves, light penetration and availability are strongly decreased on summer days in woodlands and fruit orchards that constitute the natural habitat of *Drosophila* (see Materials and Methods). Moreover, flies actively seek low light intensities, particularly at warmer temperatures (*53*–*56*). We thus applied lower light intensity in our laboratory-simulated summer days (LD 16:8 low light, 28°C).

**Fig. 1.**
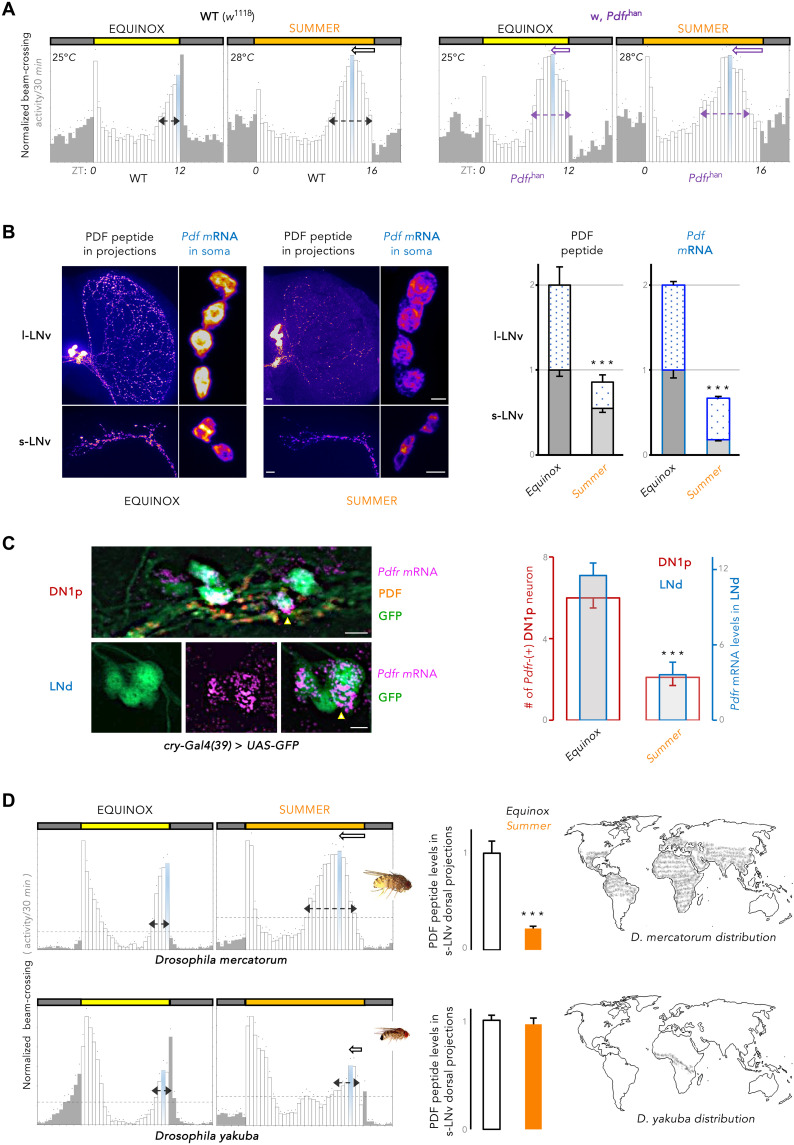
Broad and advanced evening peak of summer days is linked to seasonally weakened PDF signaling tone in cosmopolitan drosophilids. (**A**) Normalized average daily locomotor activity profiles (eductions) of wild-type (WT: *w*^1118^) and *Pdfr*^han^ mutant *D. melanogaster* flies in equinox and summer conditions. White bars, activity during the light phase (colored box); gray bars, activity in the dark period (gray box); dots, SEM. The peak of evening (E) activity is marked in blue. Dotted arrows denote the width of the E-peak, and solid arrows denote advancement of the E-peak’s phase. Photoperiod and temperature conditions are mentioned on the eductions. *n* = 10, 16, 16, and 19 flies from left to right. ZT, zeitgeber time. (**B**) PDF peptide and mRNA are quantified in s-LNv and l-LNv neurons at the midpoint of the photoperiod in equinox and summer conditions. Both PDF peptide in projections and mRNA in the soma were diminished in summer conditions (*n* ≥ 14 brain hemispheres; *P* < 0.0001 after unpaired two-tailed Student’s *t* test; error bar, SEM). (**C**) *Pdfr* mRNA detected by RNAscope in a subset of the LNd and DN1p neurons. On summer days, *Pdfr* level was lower in the LNds, and fewer DN1ps could be colabeled with the *Pdfr* probe (*n* ≥ 10 brain hemispheres; *P* < 0.0001 after unpaired two-tailed Student’s *t* test). (**D**) Cosmopolitan *D. mercatorum* is distributed widely as opposed to Afrotropical *D. yakuba.* Normalized average daily profile of *D. mercatorum* and *D. yakuba* locomotor activity in equinox and summer conditions (*n* = 15 to 17 flies). Like *D. melanogaster* and unlike *D. yakuba*, cosmopolitan *D. mercatorum* shows broadened and advanced evening (E) activity in summer, correlated with the lowering of PDF peptide level in the s-LNv dorsal termini (*n* ≥ 8 brain hemispheres; *P* < 0.0001 after unpaired two-tailed Student’s *t* test). ***P < 0.001.

A broader peak starting much earlier before lights-OFF was observed in temperate summer–like conditions (Fig. 1A and fig. S1A). Under these conditions, other laboratory strains and recently wild-caught flies from high latitudes also exhibited a broad evening peak that advanced in phase from the lights-OFF transition (fig. S1A). Since the absence of PDF advances the evening activity in laboratory LD 12:12 cycles (*46*), we asked whether environmental cues could be encoded by PDF signaling itself. In the absence of PDF signaling, an unexpectedly small effect of the environmental conditions was observed on evening activity, barring a modest increase in phase advancement (Fig. 1A and fig. S1A). This suggested that the repatterning of daily activity in summer-like conditions could be brought about by a change of PDF signaling. We thus quantified PDF levels under the two different conditions. In summer-like conditions, PDF peptide and *Pdf* mRNA were greatly diminished in the two subsets of ventral lateral neurons (LNvs) (Fig. 1B). In parallel, expression of the PDF receptor gene *(Pdfr)* also plummeted in the dorsal lateral neurons (LNds) and dorsal neurons 1, posterior (DN1ps) (Fig. 1C), which play key role in the control of evening activity (*10*, *11*, *16*, *17*). The absence of PDF decreased *Pdfr* expression and the absence of PDFR decreased PDF levels (fig. S1B). Therefore, we inferred that temperate summer–like conditions are marked by a coordinated drop in levels of PDF and its receptor.

On summer days, a broad afternoon peak is also displayed by many high latitude–restricted *Drosophila* species even if the photoperiodic adaptation strategy varies among these northern flies (*7*, *38*–*40*, *57*, *58*). In light of the changes in the activity pattern and PDF levels that we observed in *D. melanogaster*, we asked how other drosophilids adapt to summer-like conditions. Distantly related *D. mercatorum*, cosmopolitan in distribution like *D. melanogaster*, exhibited pronounced broadening of the evening activity and phase advancement of the evening peak in summer-like conditions (Fig. 1D and fig. S1C) (*58*). In contrast, the equatorial *D. yakuba* had restricted and delayed evening activity when confronted with high-latitude summer-like conditions (Fig. 1D and fig. S1C) (*59*). As observed in *D. melanogaster* on temperate summer days, PDF levels in the s-LNv axonal termini strongly decreased in *D. mercantorum*; conversely, PDF levels did not change between standard and summer-like conditions in the Afrotropical *D. yakuba* flies (Fig. 1D). *D. malerkotliana* flies, which have an intermediate latitudinal range, also underwent PDF decrease in summer days, accompanied by the pronouncement of its evening peak (fig. S1, C and D) (*60*). In temperate summer–like conditions, a broad early evening peak of activity thus appears to be correlated with a decrease of PDF signaling in cosmopolitan *Drosophila* species.

### A PDF-PKA pathway delays evening activity by inhibiting SGG-dependent TIM degradation

**Fig. 2.**
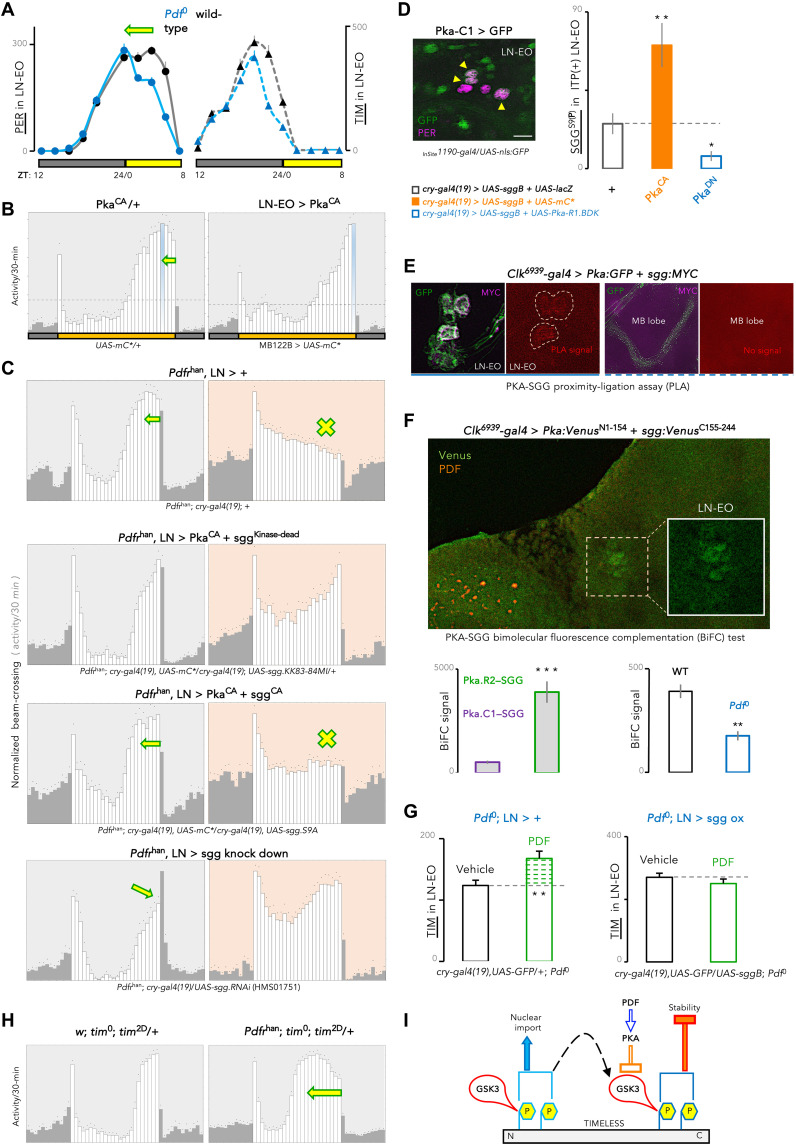
PDF-regulated PKA signaling working through SGG/GSK3 impinges on TIM to delay the LN-EO clockwork. (**A**) In 12:12 LD cycle, TIM and, subsequently, PER levels diminished significantly faster [*P* < 0.001 by two-way analysis of variance (ANOVA)] toward the end of the night in *Pdf*^0^ flies (*n* = 8-14 brain hemispheres per time point). (**B**) Under low-PDF summer conditions, forced up-regulation of PDFR signaling in the LN-EO through Pka^CA^ expression, set the advanced E peak (arrow) back to the lights-OFF transition. (**C**) Pka^CA^ expression or *sgg* knockdown rescued the advanced E peak of LD as well as the E-activity loss in RD of *Pdfr^han^* flies. Coexpression of Pka^CA^ and sgg^CA^ canceled this rescue. (**D**) In the LN-EO (left) expressing the *Pka-C1* gene, (right) catalytically inhibited S9-phosphorylated SGG increased (*P* < 0.01, *t* test) upon Pka^CA^ expression, and decreased (*P* < 0.05, *t* test) upon PKA down-regulation (R1.BDK). ZT6; *n* ≥ 14 hemispheres. (**E**) Positive PLA signal (red) between MYC-tagged SGG (purple) and GFP-tagged PKA holoenzyme (green) in the LN-EO soma at ZT2–4, but not in the mushroom body (MB). (**F**) In the LN-EO, reconstitution of *Venus* signal (green) through BiFC between SGG and PKA-C1, and SGG and PKA-R2. PKA-SGG interaction weakened (*P* < 0.01, *t* test) in the absence of PDF. *n* ≥ 10 hemispheres. (**G**) PDF (0.1 mM, for 3.5 hours at ZT14) stabilized TIM in LN-EO neurons of ex vivo *Pdf^01^* brains (*P* < 0.01, *t* test), but not with SGG overexpression (*P* = 0.17; *t* test). *n* ≥ 10 hemispheres (TIM signal is experiment-dependent and not cross-panel comparable). (**H**) Advanced E peak in *Pdfr^han^* flies with a variant of TIM (TIM^2D^) bearing phosphomimetic substitutions of two SGG-targeted amino acids. (**I**) PDF-activated PKA signaling inhibits SGG/GSK3 kinase activity, slowing TIM turnover. Low summer PDF relieves this inhibition on SGG kinase, accelerating TIM turnover. ***P < 0.001, **P < 0.01, and *P < 0.05.

To understand how weakened PDFR signaling on summer days broadens and advances the evening peak, we sought to determine whether the loss of PDF speeds up the evening oscillator in the CRY-positive PDF-negative LNs (LN-EO). In LD 12:12, TIM and PER proteins of *Pdf^0^* flies normally accumulated until the middle of the night, but then, TIM levels and subsequently PER levels decreased significantly faster than in the wild type (Fig. 2A). This suggested that enhanced PER/TIM degradation at the end of the night advances the evening peak in the absence of PDF.

Protein kinase A (PKA) signaling is involved in the PDF-dependent regulation of circadian behavior (*61*). However, PDFR signaling controls neuronal activity and oscillator properties also through PKA-independent pathways (*61*, *62*). We thus sought to determine whether the PKA pathway underlies the phase advancement of the E peak on temperate summer days, given that constitutively active PKA (PKA^CA^) offsets the advanced evening peak of *Pdfr^han^* mutants on equinox days (*61*). The phase-advancing effect of the summertime drop in PDF could be circumvented by overactivation of PDFR signaling in the LN-EO through PKA^CA^ expression (Fig. 2B).

In PDF-negative neurons, PKA promotes PER and TIM stability (*61*–*63*), but the mechanism is not known. The SHAGGY (SGG) kinase accelerates the clock by phosphorylating TIM (*64*, *65*), and SGG’s mammalian ortholog glycogen synthase kinase 3 (GSK3) is inhibited through Ser-9 phosphorylation by PKA (*66*, *67*). We thus hypothesized that SGG/GSK3 could be inhibited by PKA in the PDFR signaling pathway. We predicted that a simultaneous increase in SGG and PKA activity would inhibit PKA^CA^’s ability to rescue the advanced E peak of *Pdfr^han^* mutant (*61*). Constitutively active SGG^CA^ nullified the PKA^CA^-dependent rescue, whereas SGG knockdown rescued the advanced evening activity of *Pdfr^han^* flies having low PKA activity (Fig. 2C, left). The PDFR-PKA signaling pathway thus appears to delay evening activity by counteracting SGG. Under red-light LD (RD) cycles that prevent CRY activation, *Pdfr^han^* mutants lose the evening peak (*48*). We took advantage of this robust, qualitative RD phenotype to demonstrate the link between PDFR, PKA, and SGG. *Pdfr^han^* mutants expressing PKA^CA^ got back the evening peak in RD but not if SGG^CA^ was simultaneously present (Fig. 2C, right). Furthermore, SGG knockdown restored the evening peak of *Pdfr^han^* flies in RD (Fig. 2C, right). Together, the LD and RD results support that the PDFR-PKA pathway inhibits SGG to properly phase evening activity.

We directly assessed SGG S9 phosphorylation upon alteration of PKA activity in the LN-EO, which endogenously expresses the *Pka-C1* gene (Fig. 2D, left). PKA^CA^ produced significantly higher levels of S9-phosphorylated SGG, whereas a dominant negative PKA^DN^ reduced S9 phosphorylation (Fig. 2D, right). We then went on to examine whether PKA phosphorylates SGG by directly engaging with it. In situ proximity ligation assay (PLA) with green fluorescent protein (GFP)–tagged PKA and MYC-tagged SGG revealed evidence for PKA-SGG physical interaction in the cell body of the LN-EO (Fig. 2E). Bimolecular fluorescence complementation (BiFC) validated cytosolic interaction between PKA and SGG, further demonstrating that SGG interacted more strongly with the R2 regulatory subunit of PKA than with the C1 catalytic subunit (Fig. 2F). PKA-SGG interaction was weaker in *Pdf*^0^ mutant (Fig. 2F). This suggested that PDF signaling facilitates PKA-SGG interaction in the LN-EO, promoting the observed SGG S9 phosphorylation that signifies reduced SGG kinase activity.

Decreased PKA activity destabilizes TIM even in the absence of PER (*61*), and *Pdf*^0^ flies had faster decay of TIM and PER levels, with effect on TIM preceding that of PER (Fig. 2A). This suggested that the PDFR-PKA signaling would inhibit SGG to stabilize TIM. Bath-applied PDF peptide on ex vivo *Pdf*^0^ brains indeed increased TIM levels at night, at a time when *Pdfr* expression crests in the LN-EO (*68*), and the effect on TIM stabilization was annulled by SGG overexpression (Fig. 2G). To investigate how the PDF-PKA pathway controls SGG-dependent TIM phosphorylation, we used the TIM^2D^ variant that bears phosphomimetic modifications of two N-terminal amino acids targeted by SGG (*65*). *Tim*^2D^ flies only had a slight advance of the evening peak in LD conditions but displayed a dramatic phase advance in *Pdfr^han^* background (Fig. 2H and fig. S2A). Conversely, when the downstream target of the PDFR-PKA-SGG pathway, i.e., TIM, bears N-terminal phosphoinhibitory amino acids (TIM^2A^) on which SGG cannot act anymore (*65*), loss of PDFR signaling did not exert any discernible effect on the E-peak phase (Sup. Fig. 2B). These strong, complementary genetic interactions suggest that PDFR signaling may inhibit SGG phosphorylation at a secondary site whose phospho-occupancy is facilitated by earlier action of SGG on the N-terminal part of TIM (Fig. 2I). Together, our results strongly support a model where PDF delays the evening activity through a PKA-SGG kinase relay that stabilizes TIM at night. Thus, during temperate summer–like conditions, low PDF would induce TIM destabilization, thereby advancing the clock in PDFR-expressing evening cells.

### A specific DN1p-resident oscillator broadens the evening activity peak on summer-like conditions

Next, we set out to identify the clock neurons that are involved in the broadening of the evening peak in summer-like conditions. Evening activity is contributed by both the LN-EO and the DN1ps, while the contribution of the DN1ps is distinctly environment-sensitive and gated by PDF signaling (*10*, *11*, *69*). We thus hypothesized that a DN1p oscillator could sculpt the daily activity pattern on summer days in response to low PDF, in association with the PDFR-expressing LN-EO. DN1p neurons carrying an LL (constant light) oscillator have been postulated (*30*, *70*) and could be a good candidate for adapting evening activity to summer days.

**Fig. 3.**
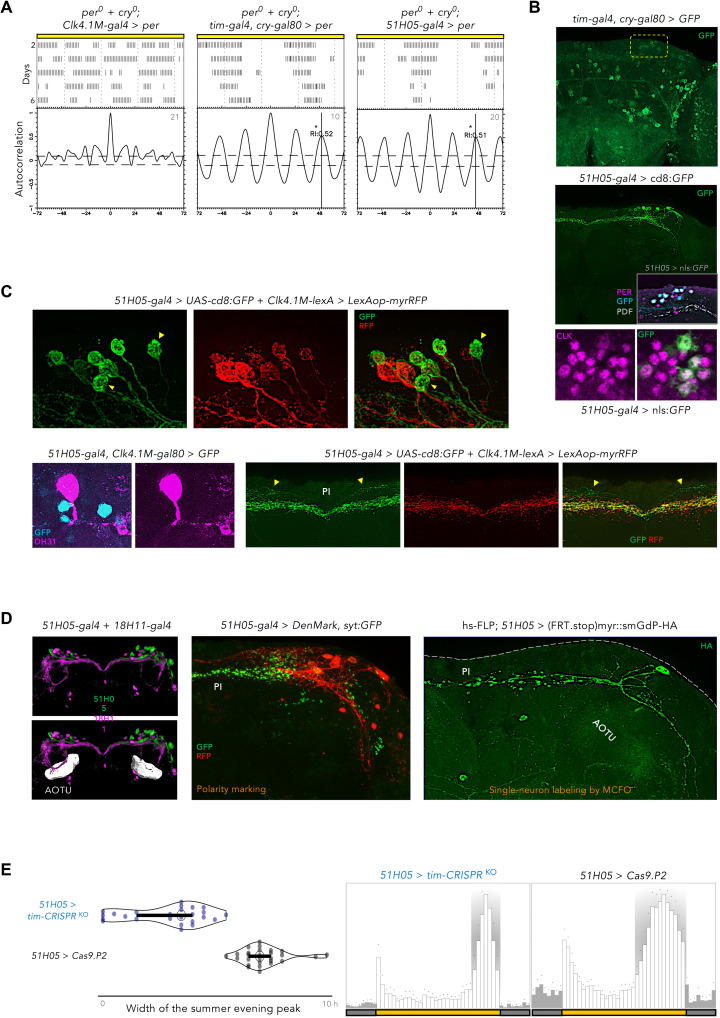
A novel, self-sustaining oscillator in the *vc*DN1p neurons is required for seasonal widening of the evening peak. (**A**) Double-plotted actograms of locomotor activity in LL (constant light). In absence of CRY (cryptochrome), functional clock was reinstated in *Clk4.1 M-gal4*, *tim-gal4.cry-gal80*, and *51H05-gal4* neurons. Autocorrelation analysis shows that both tim-gal4.cry-gal80 and 51H05-gal4 neurons could drive free-running LL rhythms, whereas *Clk4.1 M-gal4* neurons could not. *n* is marked on the corresponding autocorrelograms. (**B**) (Top) Expression pattern driven by *tim-gal4.cry-gal80* (green): The dorsal neurons are highlighted by the yellow box. (Bottom) Expression pattern of *51H05-gal4* (green): Eight to nine clock neurons (purple: PER^+^, CLK^+^) are detected, belonging to the DN1p cluster. (**C**) (Top) Some *51H05-gal4* DN1p neurons (green) are not addressable by *Clk4.1M-lexA* (red); examples are marked with yellow arrowheads. (Bottom left) These *51H05*-positive but *Clk4.1M*–negative DN1ps (cyan) do not express DH31 (purple). (Bottom right) *51H05* DN1ps (green) project additional neurites (yellow arrowheads) to the PI/PL region, compared to *4.1M-lexA* (red). (**D**) (Left) While *18H11* DN1ps (purple), as evidenced before, innervate the AOTU neuropil prominently, *51H05* DN1ps (green) barely does. Registration and comparison of Gal4 images on a common template (*Gif*) was through BrainGazer. (Middle) *51H05* neurons send their axons mostly to the PI/PL region, marked by *syt:GFP* (green) and extend ventral dendrites (DenMark: red). (Right) single-neuron labeling by Multi-Color Flip-Out (MCFO) show a *51H05* DN1ps projecting contralaterally and ventrally. (**E**) A functional clock in *51H05* DN1ps is required for building the summer-specific expanded evening (E) peak. Expression of *tim*-CRISPR^KO^ in *51H05* collapsed the wide E peak of summer days (*P* < 0.001 by Mann-Whitney *U* test).

Restriction of the functional clock to the *Clk4.1-GAL4–*expressing DN1p neurons failed to generate LL rhythms (Fig. 3A). However, we observed that PER rescue in most CRY-negative dorsal clock neurons (defined by the *tim-GAL4.cry-GAL80* driver combination) resulted in robust LL rhythms (Fig. 3A). To narrow down the key neuronal group, we tested several sparser *GAL4* lines driving expression in various subsets of clock neurons and observed rhythmic LL behavior when rescuing PER with the *51H05-GAL4* driver (Fig. 3A). The *51H05-GAL4* targets a subset of the glutamatergic DN1ps (Fig. 3B) (*71*). In each brain hemisphere, we observed about eight to nine *R51H05-*labeled neurons, with more than half of them being weakly colabeled by the *Clk4.1* driver (Fig. 3, B and C). The two to four *51H05* neurons that were excluded from *Clk4.1* labeling did not express the arousal-promoting (*71*) DH31 neuropeptide (Fig. 3C). In comparison to the *Clk4.*1 DN1ps, the *51H05* DN1p*s* extended additional dorsal neurites in the pars intercerebralis (PI) region (Fig. 3C). Notable nonoverlap in the projection patterns of *51H05* and the previously characterized *18H11* DN1p*s* (*71*–*74*) was also apparent (Fig. 3D). Unlike *18H11*, axons of the *51H05* DN1p*s* scantily innervated the anterior optic tubercle (AOTU) neuropil, if at all (Fig. 3D). Multicolor flip-out labeling (Fig. 3D) demonstrated that most *51H05* DN1p*s* belongs to the ventrally and contralaterally projecting *vc*DN1p group (*69*, *74*), whose role in clock-regulated behaviors remains unexplored (*73*–*75*).

To reveal the clock function of this novel DN1p oscillator, we rendered it clockless by expressing *tim*-CRISPR^KO^ and *cyc^DN^*. Targeted knockout of the clock in *51H05* as well as *tim-GAL4.cry- GAL80* neurons suppressed the broadening of the evening activity in summer-like conditions (Fig. 3E and fig. S3). This suggested that the circadian oscillator of the *51H05* DN1ps acts in the evening to either promote a rest-inhibiting function or suppress a rest-promoting function, thereby carving the characteristic evening peak of temperate summer days.

### Working through SIF*a* neurons, *51H05* DN1p*s* link PDF input to daytime rest

To examine the direct impact of the *51H05* neurons on locomotor behavior in summer conditions, we manipulated their firing activity. Silencing *51H05* neurons with Kir2.1 expression (*76*) allowed additional expansion of the summer days’ evening peak, whereas hyperexciting them with heat-activated TrpA1 (*77*) constricted it (fig. S4A). As their constant hyperexcitation recapitulated the phenotype of clock loss (Fig. 3E), we inferred that oscillator function in the *51H05* DN1p neurons is required for timed down-regulation of their rest-promoting neural activity.

**Fig. 4.**
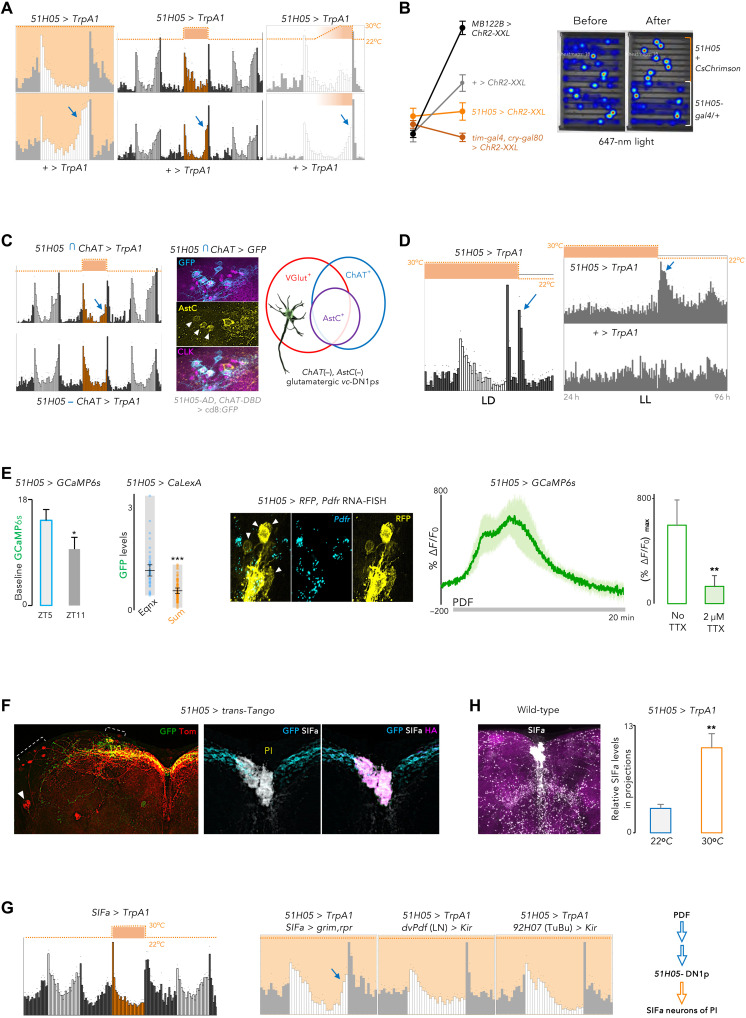
On equinox days, high levels of PDF activate 51H05→SIF*a* circuit to promote daytime quiescence. (**A**) Overactivation of *51H05*-DN1ps during the 24-hour day (left), 12-hour photophase (middle), or in the afternoon (right), suppressed the equinox E peak (*n* = 10 to 16). (**B**) (Left) Acute activation of DN1ps suppressed locomotor activity, contrasting with the arousal-promoting effect of LN-EO (*MB122B*) (*n* = 10 to 16). (Right) Position heatmaps visualize quiescence induced by DN1p activation (647-nm light of 4500 lux applied at 5 Hz for 10 min). (**C**) (Left) Hyperexcitation of noncholinergic *51H05* neurons (bottom), but not cholinergic ones (top) abolished the evening peak (*n* = 10 to 16). (Middle) AstC (yellow) was restricted to cholinergic *51H05* neurons (cyan, arrowheads). (Right) Neurochemical characterization of the DN1ps. (**D**) Deactivation of *51H05* generated locomotor peaks in LD (left), and clock-disabling LL (right), *n* = 10 to 16. (**E**) (Left) *51H05* DN1ps were more active at ZT5 versus ZT11 (*P* < 0.05, *t* test, *n* = 8 per time point). Summer conditions reduced their midday activity (*P* < 0.01, *U* test, *n* = 6 to 8). (Middle) Most *51H05* DN1ps lacked *Pdfr* mRNA (yellow, white arrowheads, via RNAscope). (Right) PDF triggered a Ca^2+^ rise in the *51H05* neurons in a TTX-dependent fashion (*P* < 0.01, *t* test, 30 μM PDF, *n* = 8 representative neurons from five brains). (**F**) Trans-Tango uncovered LNds (white arrowhead), DNs, and SIF*a* expressing PI neurons as direct downstream targets (red and magenta) of the *51H05* DN1ps (green and cyan). (**G**) (Left) Overactivation of SIFa neurons during the 12-hour photophase abolished E peak. (Middle) Their functional removal, but not of LNs or TuBu neurons, rescued the effect of *51H05* hyperexcitation (*n* = 10 to 16). (**H**) Twelve-hour-long *51H05* overactivation elevated SIFa peptide levels in the projections of the PI neurons around the fan-shaped body (*P* < 0.01, by *t* test, *n* ≥ 5 brains per condition; error bar, SEM). **P < 0.01.

To further characterize *51H05* neurons’ function, we activated them in LD 12:12 conditions. Hyperexcitation of the *51H05* (or *tim-GAL4.cry-GAL80*) neurons with TrpA1 abolished the evening peak in equinox days (Fig. 4A). Collapse of the evening peak was still apparent when the overexcitation was restricted to the daytime or even just the afternoon (Fig. 4A). Optogenetic activation with ChR2-XXL (*78*) underscored that the newly revealed DN1p-based oscillator, in stark contrast to the LN-EO, suppresses locomotor activity (Fig. 4B). Video recording of flies undergoing CsChrimson-mediated brief activation of the *51H05* neurons confirmed their lasting quiescence-triggering role (Fig. 4B).

Taking advantage of intersectional genetics, we proceeded to ascribe the quiescence- promoting role to more restricted subsets of the *51H05* neurons. Hyperexcitation of the *ChAT*(+) *51H05* DN1p*s* could not abolish the evening peak, while the *ChAT*(−) *51H05*-DN1p*s* could (Fig. 4C). Our results highlighted the noncholinergic *51H05*-DN1p*s* that do not express the AstC neuropeptide as the core subset of the locomotor-suppressive DN1p*s* (Fig. 4C).

The locomotor-suppressive *51H05* oscillator working alone could produce regular peaks of behavioral activity in LL in the absence of *cry* (Fig. 3A). We asked whether timed inhibition of these quiescence-promoting neurons, all by itself, could generate peaks of locomotor activity. Cessation of hyperexcitation of the *51H05* neurons could produce an additional, i.e., third activity peak at nighttime in LD conditions (Fig. 4D). The same manipulation could carve an activity peak even when the flies had previously been made arrhythmic by exposure to LL in the presence of *cry* (Fig. 4D). Thus, falling neural activity in activity-suppressive neurons can sculpt a peak of locomotor activity.

These results prompted us to posit that the *51H05* DN1p neurons would promote midday siesta and counteract the arousal-promotive effect of the LN-EO in the early afternoon. In other words, neural activity of the *51H05* neurons should be high around midday and then go down during the afternoon, allowing the manifestation of the evening anticipation. Basal GCaMP6*s* signal in the *51H05* neurons was significantly lower (Fig. 4E) before the lights-OFF transition, when the neural activity of the LN-EO crests (*19*, *75*, *79*). On temperate summer days, the evening bout of locomotor activity gets wider and the *51H05* neurons stayed hypoactive as reflected by the CaLexA reporter (Fig. 4E). We thus inferred that the summertime hypoactivity of the *51H05* neurons results from the attenuation of PDF signaling since PDF increased neural activity of the *51H05* DN1p*s* (Fig. 4E). The lack of *Pdfr* expression in most *51H05* cells (Fig. 4E) suggested an indirect pathway. PDF response of these DN1p neurons was down-regulated by tetrodotoxin (TTX) pretreatment (Fig. 4E), corroborating the participation of interneurons in the relay of the PDF message. Notably, single-cell RNA–based transcriptomic database of the clock neurons (*68*) supports the existence of a glutamatergic DN1p subclass that expresses neither *ChAT*, nor *Dh31*, *AstC*, and *Pdfr*.

To explore the output pathways downstream of the *51H05* DN1ps, we used trans-Tango (*80*). In addition to few DN and LNd neurons, four median neurosecretory cells of the PI were found to be postsynaptic to the *51H05* DN1p*s* (Fig. 4F). These PI neurons were marked by SIF*amide* (SIF*a*) expression (Fig. 4F). Notably, a different subset of the *Pdfr*(+) CRY(+) DN1ps were shown to act upstream of the SIF*a* neurons in a memory extinction circuit (*81*). Although SIF*a* has a limited impact on rest-activity rhythms (*82*, *83*), depletion of SIF*a* leads to hyperactivity (*84*), and activation of SIF*a* neurons promotes sleep (*85*). TrpA1-mediated activation of the SIF*a* neurons during the 12 hours of daytime completely suppressed the evening bout of locomotor activity (Fig. 4G), mirroring the effect of *51H05* neuron overactivation. Ablation of the SIF*a* neurons abolished the locomotor-suppressive effect of *51H05* overactivation (Fig. 4G). In contrast, silencing of neither the LNs nor the TuBu neurons of the AOTU, the known downstream targets of the DN1p*s* (*69*, *72*–*75*, *86*), could override the effect of *51H05* activation (Fig. 4G).

Drosophila EM connectome revealed synapses between the DN1p and SIF*a* neurons (*87*). Twelve-hour-long activation of the *51H05* neurons elevated SIF*a* levels in the projections of the PI neurons (Fig. 4H). In addition, pharmacogenetic excitation of the *51H05* cells induced calcium rise in SIF*a* neurons (fig. S4B). Together, the results indicate that *51H05* neurons directly activate rest-promoting SIF*a* neurons of the PI to attain quiescence. Their reduced activity in low PDF summer-like conditions is thus expected to induce a broadening of the evening peak.

### PDF-induced changes in oscillator coupling confer a prominent role to the DN1p clock in summer-like conditions

**Fig. 5.**
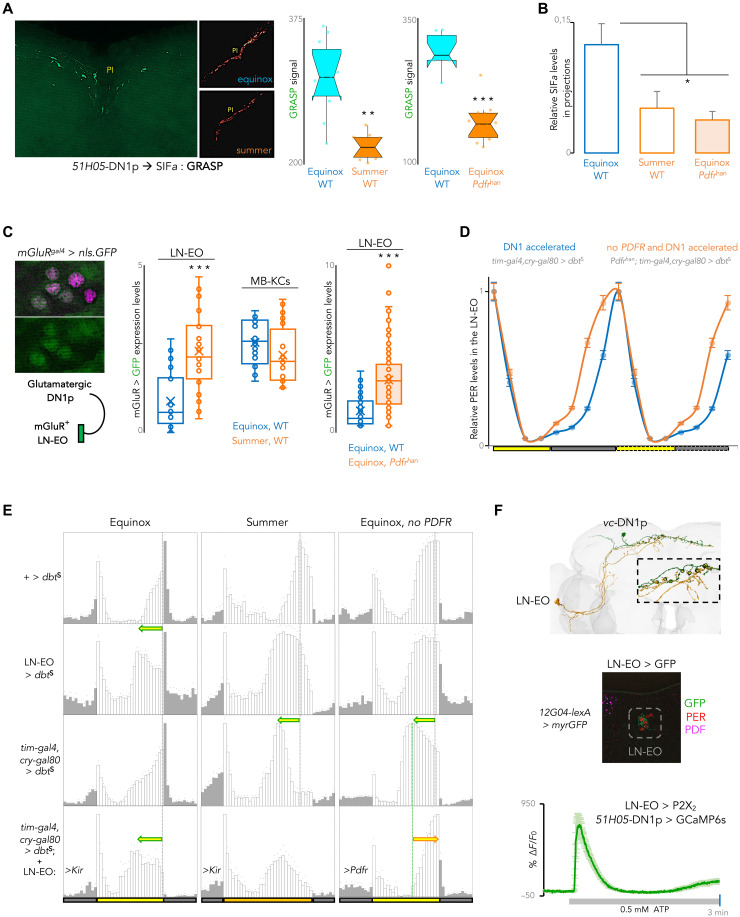
Rewired summer couplings accentuate DN1ps’ status in network hierarchy. (**A**) GRASP revealed weakened anatomical connectivity between the *51H05* DN1p and SIFa^+^ PI neurons in summer, and in *Pdfr* mutants in equinox (**P < 0.01 and ***P < 0.001, Mann-Whitney *U* test, *n* = 6 to 8 per condition, dissected at the middle of the photoperiod). (**B**) SIF*a* peptide levels in PI neurons’ projections were diminished in summer, or in the absence of *Pdfr* in equinox days (*P* < 0.05 by one-way ANOVA with Tukey’s post hoc test, *n* = 5 to 6 brains per condition, dissected at the middle of the photoperiod). (**C**) A T2A-Gal4 knock-in in *mGluR* drove nuclear-GFP (green) expression in LN-EO neurons (PER, purple). DN1 to LNd communication relies on *mGluR* ^68^. *mGluR* expression in the LN-EO increased in summer and in *Pdfr* mutants in equinox but not in the *mGluR*-expressing Kenyon cells (MB-KCs) (***P < 0.001, *n* = 6 to 8 brains per condition, dissected at the middle of the photoperiod). (**D**) In *Pdfr* mutants, DBT^S^-driven DN1p acceleration advanced PER oscillations in the LN-EO (*P* < 0.001, two-way ANOVA, *n* = 5 to 8 brains per time point), suggesting a stronger interoscillator (DN1p→LN-EO) coupling. (**E**) (First to third rows) On equinox days, LN-EO determined the E-peak phase. However, DN1ps assumed this role in summer or without *Pdfr*. (Fourth row) LN-EO silencing unmasked DN1p’s control in the presence of *Pdfr*, revealing a PDF-dependent conditional hierarchy for phase control (*n* = 10 to 16). (**F**) FlyWire reconstructions confirmed input synapses (yellow dot on inset) from ITP(+) LN-EO (720575940625254636, ochre) to *vc*DN1p (720575940644609440, green). The hemibrain connectome contains recurrent synapses between the ITP(+) LN-EO and DN1pA (*vc*DN1p). (middle) The LN-EO neurons were addressed through the sparse *12G04-LexA* driver. (right) P2X2-mediated LN-EO activation induced calcium rise in *51H05* DN1ps (*n* = 8), underscoring functional connectivity.

We set out to determine how the novel DN1p oscillator senses PDF and imparts its command to the downstream circuit elements. With the SIF*a* neurons of the PI, *51H05* DN1p*s* displayed prominent GRASP (GFP Reconstitution Across Synaptic Partners), indicative of their close anatomical proximity (Fig. 5A). Notable diminution of this GRASP signal was seen in summer-like conditions (Fig. 5A). Likewise, diminished GRASP was apparent in flies lacking *Pdfr* (Fig. 5A). Levels of the SIF*a* peptide in projections of the PI neurons also went down on summer days (Fig. 5B), and *Pdfr^han^* mutants recapitulated this loss of SIF*a* peptide on standard equinox days (Fig. 5B). Therefore, the feedforward input from the *51H05* DN1p*s* to the SIF*a* neurons wanes under temperate summer conditions in a PDF-dependent manner, reducing the levels of the SIF*a* neuropeptide in the brain. We infer that decreased PDF levels in summer conditions would be sensed by the LN-EO to inform the PDFR(−) *51H05* DN1ps about the environment. Notably, *51H05*–LN-EO anatomical connectivity did not change with seasons (fig. S5A), suggesting targeted structural plasticity in the *51H05*–SIF*a* pathway.

*51H05* DN1p*s* extend prominent projections ventrally toward the LN-EO, the postsynaptic targets of the *51H05* neuron*s* confirmed by trans-Tango (Fig. 4F). *mGluR* in the LN-EO mediates glutamate reception from the DN1p*s* (*72*). We asked whether this glutamatergic pathway manifests seasonal plasticity. In wild-type flies, *mGluR*-driven GFP levels, a proxy for *mGluR* expression in the LN-EO, increased under summer-like conditions (Fig. 5C). Other *mGluR*(+) neurons, e.g., the Kenyon cells of the mushroom body did not exhibit summer-driven enhancement of *mGluR* expression (Fig. 5C). *mGluR* expression in the LN-EO also increased in *Pdfr^han^* flies on standard equinox days (Fig. 5C). These results suggested that the connection strength between the glutamatergic DN1p*s* and the LN-EO is increased by weakened PDFR signaling under temperate summer conditions. Therefore, coupling between DN1ps and LN-EO should become stronger in *Pdfr^han^* flies even under standard equinox conditions. When DN1p*s* were forced to run faster, PER oscillations in the LN-EO were advanced in *Pdfr^han^* mutants in comparison to control flies (Fig. 5D). Summer-like conditions thus increase the control of the LN-EO by the *51H05* DN1p*s*.

**Fig. 6.**
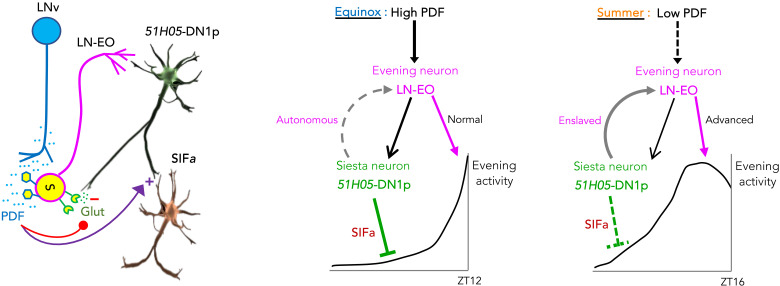
A neuropeptide-directed bilayered plasticity mechanism adapts the evening peak of locomotor activity to seasonal changes in environmental conditions. Our working model explains how seasonal remodeling of the evening activity bout is accomplished. PDF(+) LNv neurons send input to the *51H05* DN1ps via PDFR-expressing LN-EO neurons. These DN1ps in turn feedback on the LN-EO and also communicate with downstream SIF*a*(+) medial neurosecretory cells that promote daytime quiescence. PDFR signaling down-regulates the DN1p to LN-EO connection and up-regulates DN1p to SIF*a* connectivity. On summer days, attenuation of the PDF-PDFR signaling tone weakens DN1ps’ quiescence-promoting output on SIF*a* neurons and thereby expands the evening bout of locomotor activity. Moreover, attenuated PDF*ergic* tone in summer potentiates the *51H05* DN1p oscillator’s authority over the LN-EO pacemaker, leading to the emergence of a new chain of command for phasing evening activity. The evening oscillators accelerate as PDF levels drop. As a result, the summer evening peak starts to form earlier during the afternoon. Together, opposing and flexible changes along distributed circuit nodes orchestrate seasonal expansion and advancement of the evening activity in *Drosophila*.

To understand how the LN-EO and the *51H05* DN1ps interact to adapt the evening activity to summer-like conditions, we manipulated the clock of each oscillator and assessed the behavioral consequence in standard and summer-like conditions, as well as in standard conditions with no PDFR signaling. Accelerating the ITP^+^ clock neurons (key constituent of the LN-EO) advanced the evening peak more in standard conditions than in summer-like conditions (Fig. 5E and fig. S5B). In contrast, acceleration of the DN1p clock had no effect in equinox conditions but strongly advanced the evening peak on summer conditions or in the absence of PDFR (Fig. 5E and fig. S5B). Thus, absent or reduced PDFR signaling allows DN1p to conditionally gain control over the phasing of evening activity, whereas high PDF cancels their contribution to phasing. Selectively restoring PDFR signaling in the LN-EO rescinded the control of the DN1p*s* on evening phase (Fig. 5E and fig. S5B). Hence, PDF signal is measured by the LN-EO, which then gates the contribution of the DN1p clock. Connectome data indicate the presence of input synapses from the LN-EO to the *vc*DN1ps and activating the *12G04*-LexA–expressing LN-EO neurons induced Ca2^+^ increase in the *vc*DN1ps, supporting a functional connection between the two groups (Fig. 5F). This implies that functional synaptic connections between the LN-EO and *vc*DN1ps are leveraged to broadcast PDF information through the clock network. When the LN-EO was incapacitated through *Kir* expression, the phase of the evening peak was determined by the DN1p clock in both standard and summer-like conditions (Fig. 5E). Together, our data support a model where PDF sensing by the LN-EO, on one hand, increases the rest-promoting function of the *51H05* DN1ps and, on the other hand, decreases the contribution of the *51H05* DN1p clock to the phasing of the evening peak (Fig. 6).

## DISCUSSION

### Neuropeptide dynamics mirror external environmental conditions in the brain

Adapting the activity profile to seasonally varying light and temperature conditions is a key part of fitness in nonequatorial animal species. Our work centered on the evening activity bout of *Drosophila* uncovers key molecular and circuit mechanisms of this adaptive plasticity. Our behavioral data reveal that flies exposed to temperate summer days show a broader and advanced evening activity in comparison to standard laboratory conditions (equinox days). This behavioral adaptability is largely reduced in flies lacking PDF, suggesting that PDF neuropeptide is involved in adaptation to summer days. Wild-type flies have decreased levels of both PDF and PDFR in temperate summer days. The advanced evening peak and the decreased PDF levels in summer conditions are also observed in the *D. mercatorum* and *D. malerkotliana* species, which have a broad latitudinal distribution. Since these correlated behavioral and PDF changes are not recapitulated by equatorial drosophila species like the *D. Yakuba*, we hypothesize that PDF- dependent behavioral change is restricted to species that encounter strong seasonal variations in their environment. In contrast to the *D. melanogaster* flies, photoperiodic adjustment of the evening peak is also absent in the equatorial island species *D. sechellia*, and this difference has been attributed to divergence in *Pdf* gene regulation between these two species (*88*). At the other extreme, the evening peak of many subarctic *Drosophila* species under polar summer conditions also shows greater prominence, marked by a broader activity window and an advanced phase (*7*, *39*). In the temperate zone, distinctly long daylength occurs around the summer solstice, yet daily temperature and light maxima rarely reach obnoxious levels. Over the late afternoon, weather conditions in the temperate zone likely become optimized for the heterothermic fruit fly to be most active at least energetic cost. Melanogaster flies under laboratory-simulated as well as outdoor experiments tested in Leicester (52.6°N) around mid-July displayed mostly a single prominent daily activity peak in the late afternoon, shaped broadly and advanced in phase (*34*, *89*). Using *Pdf*^0^ mutants, these studies also suggested that PDF signaling modulates the temperature-dependent onset of the evening peak under seminatural conditions (*89*). Our findings demonstrate that PDF plays a major role in the adaptation of the evening peak to temperate zone summer days.

High-latitude flies from the *Drosophila* subgenus *virilis-repleta*, which thrive in temperate summers, do not have PDF in the small ventral lateral neurons (sLNvs) (*39*, *58*). Consistent with this, in *D. melanogaster*, the decrease of PDF immunoreactivity in temperate summer days that we uncover here, is associated with lower *Pdf* mRNA levels, particularly in sLNvs, suggesting a transcriptional control. Under LD laboratory conditions, PDF immunoreactivity cycles (*90*) but *Pdf* mRNA does not (*90*, *91*). On the other hand, high light induces *Pdf* mRNA increase in the sLNvs (*11*), while subphysiological cold temperatures (*92*), characteristic of harsh winter days, decrease *Pdf* mRNA (*6*, *42*). Collectively, these findings highlight that the regulation of *Pdf* mRNA levels is crucial to environmental context-dependent PDF dynamics.

Wild-type flies tend to build their evening activity earlier in the afternoon at low temperatures (*6*). The previously observed decrease in PDF levels in cold winter days could thus contribute to this phase advancement of the evening onset. Conversely, under long photoperiod (LD 16:8), wild-type melanogaster flies delay their evening activity peak, while flies lacking PDF cannot (*47*, *52*). However, even a drastic change in photoperiod from 8 to 16 hours does not notably alter the mean PDF levels or the amplitude of PDF cycling in the sLNvs (*42*). Together, these results indicate that the marked decrease in PDF observed during temperate summer days is likely due to synergistic interaction between moderately high temperature, low light intensity, and long photoperiod. PDF thus appears to be a key component to modify the locomotor behavior according to environmental variations, thus allowing adaptation to daily or seasonal environmental changes in temperature, light intensity, and photoperiod.

Other mechanisms contribute to seasonal adaptation in fruit flies. Several species of Drosophila also exhibit seasonal changes in midday activity, orchestrated by the *dmpi8-daywake* genetic unit (*93*). Temperature and light regulate the splicing efficiency of the *dmpi8* intron located in the 3′ untranslated region of the *period* gene (*6*, *59*, *94*). Under cold winter conditions, the efficient splicing of *dmpi8*, acting in trans, promotes peripheral expression of the linked anti-siesta gene *daywake*. This effect is counterbalanced by light-induced inhibition of *dmpi8* splicing, which involves the NORPA phospholipase C of the visual system (*94*–*96*). Notably, the seasonal adjustment of midday activity by the *dmpi8-daywake* mechanism occurs independently of the PER protein and without altering the core clock mechanism (*97*), while the neuropeptide-guided seasonal plasticity of the evening peak relies on the clock gene network and the clock neuronal network.

Being controlled by light, temperature, and the circadian clock, PDF levels are logically central in phasing activity within the 24-hour day-night cycle. Nevertheless, given that our model is based on highly reductive laboratory conditions mimicking midlatitude summers, complementary future studies are needed to address the importance of the PDF-driven plasticity mechanisms under seminatural outdoor conditions.

### PDF guides phase plasticity via an inhibitory PKA-SGG pathway targeting TIM

How does the biochemical clock program getting reset by PDF still remain unclear? PDFR signaling, particularly through the cyclic adenosine monophosphate (cAMP)/PKA pathway, posttranslationally controls PER/TIM half-life, but the exact effects seem to be diverse and cell specific (*61*, *63*, *98*). In addition, PDFR-dependent transcriptional regulation of clock genes affects the amplitude of the molecular rhythms (*62*, *99*).

Our results show that the LNv-derived PDF decelerates the PDFR^+^ LNd oscillator, highlighting a causal basis of the PDF-induced delay in the phase of calcium oscillations and behavioral output generated by the LNd’s evening oscillator (*46*, *79*). Subsequently, we implicate GSK3/SGG as the core clock component directly translating PDF-evoked upsurge of PKA activity into pace change of the LNd oscillator. Our data strongly suggest that SGG is part of the PDFR signaling pathway and is a direct target of PKA-catalyzed inhibitory phosphorylation. In the *Drosophila* clockwork, the principal substrate of SGG kinase is TIM (*64*, *65*). On the S297 and T301 residue, SGG-catalyzed TIM phosphorylation promotes its nuclear accumulation, albeit that the strength of this effect widely varies across clock neurons (*65*). Our work hints that these phosphorylation events facilitate additional SGG-regulated TIM modification, resulting in increased TIM turnover. Notably, sequential and hierarchical phosphorylation events are also hallmarks of CK1/DBT’s catalysis on PER (*100*), although the DBT-PER interaction, unlike the SGG-TIM interaction, is not known to vary seasonally. Notably, PDFR and SGG-influenced regulation of TIM stability and oscillations was potent in the evening oscillator neurons. The distinct composition of the PDFR signalosome between morning and evening neurons (*101*) and the distinct architecture of the biochemical clock program itself (*102*) could underpin a differential effect of PDFR on morning/evening oscillators.

Inhibitory S9 phosphorylation of SGG is strongly regulated by PKB/AKT signaling, and sensitivity of the *Drosophila* behavioral rhythms to nutrient status and energy level is thought to be mediated by AKT and TOR pathways acting on SGG activity to control nuclear accumulation of TIM (*103*). Furthermore, SGG’s action on TIM also interfaces with CRY-dependent TIM-degradation induced by light. Light-responsive serotonin/5HT1B signaling modulates TIM stability through S9 phosphorylation of SGG (*104*). TIM is the central determinant of the clock’s photosensitivity, with the different alleles of *tim* regulating the adaptation of the behavior to the photoperiod as a function of latitude (*105*–*109*). *Tim* is also regulated by ambient temperature through thermosensitive alternative splicing, which leads to changes in TIM protein dynamics (*110*–*112*). While the functional roles of the winter-dominant splice forms of *tim* have been characterized, the impact of the summer-dominant splice form (*tim-M*) on the locomotor activity peaks remains unexplored (*112*). We hypothesize that the TIM protein isoforms expressed in summer and winter might exhibit differential sensitivity to the PDFR-PKA-SGG pathway, thus contributing to the seasonal remodeling of the daily activity pattern. TIM indeed appears to sense a series of internal states and environmental changes to translate these signals into modifications of the molecular clock. The LNd evening neurons may be uniquely able to segregate the effect of different upstream pathways on SGG-TIM interaction by opting for time-dependent usage of alternatively spliced SGG isoforms.

### Neural circuit mechanism underlying seasonal waveform plasticity

We identified a small subset of glutamatergic DN1p neurons as key regulators of siesta. These two to four CRY-negative neurons are best characterized by the expression of the *51H05-Gal4* driver, which can autonomously drive sustained behavioral rhythms in LL. The DN1ps are highly heterogenous (*68*) and are involved in different sleep-promoting and sleep-inhibiting effects through other clock neurons or downstream nonclock cells (*69*, *71*–*75*, *113*). The DH31-negative, CRY-negative *51H05* neurons belong to the anatomically defined *vc*DN1p group (*74*), whose function remained unknown until now. We demonstrated that a functional clock in these DN1ps is required to broaden evening activity in temperate summer days. Overactivation of these neurons extends the afternoon siesta, whereas silencing them widens the evening peak of activity to the afternoon. PDF increases the neuronal activity of the *51H05* DN1ps. Hence, temperate summer days with low PDF levels keep them hypoactive, allowing the evening peak to expand into the afternoon.

This DN1p siesta-promoting circuit that we reveal here appears to be unrelated to the previously described DN1p-TuBu sleep-promoting and sleep-inhibiting circuits (*69*, *72*–*75*). Whether PDF has some effects on the other DN1p-controlled circuits is unknown, although PDFR is expressed in the CRY-positive glutamatergic DN1ps (*68*). Despite being PDF regulated, *51H05* DN1ps are PDFR-negative cells, indirectly receiving PDF signal through PDFR-expressing LN-EO. The LN-EO uses the PDFR-PKA-SGG pathway to phase adjust the E-peak according to seasonal PDFergic tone, yet it may convey its inferred PDF status to upstream *51H05* DN1ps via an alternative, potentially PKA-independent mechanism. Future studies should determine whether a shared molecular mediator, regulated by PDFR signaling, orchestrates summer-specific modifications in both the phase and waveform of the evening peak.

Our results also reveal a complementary interaction between the two subsets of the evening neurons: LN-EO and *51H05* DN1p. While accelerating the *51H05* DN1p clock in standard days does not affect the evening peak of activity, it dramatically advances evening activity by several hours in temperate summer days or in the absence of PDF signaling. In these summer conditions, a decrease of the sleep-promoting effect of the *51H05* DN1ps is thus associated with an increase of their contribution to the pace of the circadian network.

How could such changes occur? Internal state-dependent, axonal branch–specific anatomical plasticity is shown by specific dopaminergic neurons (PPM2) in the fly brain (*114*). In the clock network, PDF-expressing sLNvs show a daily rhythm of axonal arborization expansion (*115*–*117*). This arborization is required for the integration of temperature inputs through glutamatergic DN1ps (*118*), and the daily cycling is regulated by PDFR signaling (*119*). It is thus possible that the LNd/DN1p switch and/or the decrease of DN1p activity in low-PDF temperate summer days are regulated by related mechanisms. Similarly, on temperate summer days, *51H05* DN1p neurons alter their medial connections with the SIF*a* neurons of the PI while maintaining their ventral projections to LN-EO. We hypothesize that in low-PDF summer conditions, the hypoactive glutamatergic DN1ps strengthen their functional connections with LN-EO through increased postsynaptic mGluR expression and possibly enhanced glutamate release. Differential regulation and release of cotransmitted neurotransmitters/neuropeptides based on firing patterns and rates, as widely seen in both invertebrate and mammalian neurons (*120*, *121*), support the potential for selective strengthening of DN1p glutamatergic synapses in their ventral branch under low-PDF conditions. Given that DN1ps, like SCN clock neurons, coexpress multiple output molecules, selective and branch-specific synaptic adaptation likely represents a general mechanism for modifying the circadian neural circuitry based on internal and external states.

This switch from the LN-EO clock to the DN1p clock under low-PDF conditions may enhance the plasticity of evening activity in response to environmental changes as the DN1p clock neurons are more sensitive to temperature and light fluctuations (*10*, *11*, *50*, *69*, *122*, *123*). On harsher Mediterranean-type summer days, light, temperature, and PDF counteract circadian signals, delaying evening activity closer to nightfall (*53*, *87*). However, during temperate summer days, the circadian network takes the lead through the otherwise hypoactive *51H05* siesta neurons to, on one hand, keep evening activity elevated in the afternoon and, on the other hand, to accelerate the main evening clock, i.e., LN-EO. We speculate that higher latitude–restricted drosophilids have evening circuits permanently locked in the latter configuration.

### SIF*a* neurons link the clock to seasonal changes in systemic physiology

We show that the locomotor-suppressive dorsal clock neurons communicate with a defined subset of neurosecretory cells in the PI that express the neuropeptide SIF*a* and that activation of *51H05* neurons induces an increase in SIF*a* levels. Only two pairs of SIF*a* neurons extend projections onto the entire *Drosophila* central nervous system to regulate reproduction, food intake, and sleep (*84*, *124*–*126*). 51H05 DN1ps need SIF*a* neurons to promote siesta and overactivated SIF*a* neurons suppress the evening peak of activity, in agreement with their sleep-promoting function (*84*, *85*). Under temperate summer conditions or in the absence of PDF signaling, the anatomical connection between DN1ps and SIF*a* neurons is reduced and SIF*a* levels decrease. A drop of orexigenic SIF*a*, commanded by the upstream PDF-DN1p circuit, thus permits the fly to sustain high-level locomotor activity during temperate summer days. A similar circuit mechanism reliant on shifting connectivities of a separate LNv-DN1a circuit also underlies daytime-dependent plasticity of light-evoked locomotor response (*127*).

In mammals, efferent SCN neurons characteristically restrict their innervation within the hypothalamus. The neuroendocrine command center, i.e., PI of the insect brain displays physiological and developmental equivalence with the vertebrate hypothalamus (*128*). The PI houses regulatory centers for sleep, arousal, and rhythmic rest activity, as does the hypothalamus in mammals (*83*, *129*, *130*). Notably, the evolutionary homolog of SIF*a*R in vertebrates NPFFR1/2, senses RF amide–related peptides (RFRPs), whose hypothalamic expression varies seasonally (*131*). Widespread control of vertebrate seasonal reproduction by RFRP neurons that receive input from the SCN (*131*) suggests a potential role of SIF*a* in bridging insect reproductive physiology with the brain circadian network.

While suppressing sleep, a reduction in SIF*a* under temperate summer conditions should also facilitate mating at the expense of feeding, and consequently, reduce lifespan (*124*, *125*). This behavioral goal switch is complemented by overactivated insulin/dilp2 signaling driven by the fly’s preference for yeast intake during hot summer days (*132*, *133*). The clock neuronal network thus uses parallel light–modulated outputs via DN1p-SIF*a* and LNv-LHLK pathways that integrate sleep with hunger (*134*, *135*). It will be interesting to see how these circuits coordinate to control siesta and evening activity on summer days as a function of light levels and feeding status.

## MATERIALS AND METHODS

### Fly lines

A list of the fly lines, their source, or corresponding references are provided in the table S1. All fly stains were maintained on corn meal media at 25°C in 12:12 LD.

### Generation of transgenic flies

#### ITP-gal4

To obtain the ITP-gal4 line, the gal4 sequence included in the vector pBS-KS-attB1-2- GT-SA-Gal4-Hsp70pA (DGRC stock# 1325) was inserted in place of the transposon Mi{MIC}ITP(MI00349) contained in the fly stock RRID:BDSC_30713 using Recombination Mediated Cassette Exchange technique (*136*) as previously described (*11*). Transgenesis was performed by BestGene.

#### LexAop-PDFr-mCherry

LexAop-PDFr-mCherry transgene was constructed by replacing the myr-GFP sequence of plasmid pJFRC19-13XLexAop2-IVS-myr::GFP (pJFRC19, Addgene plasmid #26224) with the PDFr-mCherry sequence as described below. The PDFr ORF (from exon2 excepted 27 first base pairs to exon9 before last codon) sequence ends with glutamine in frame with mCherry sequence. PDFr-mCherry sequence was polymerase chain reaction (PCR) amplified from c13758 mCherry pcDNA3 (gift from P. Taghert) using two primers containing respectively SalI and XbaI restriction sites: For. 5′AT*GTCGAC*ATGACCCTCCTGTCGAACAT3′ and Rev. 5′CC*TCTAGA*GGCTACTTGTACAGCTCGTCCATG3′. It was then inserted in pJFRC19 previously digested by XhoI and XbaI restriction enzymes using T4 DNA ligase. After confirmation by sequencing, this construct was introduced inside the third chromosome attP docking site VK00005 doing a phiC31 integrase–mediated transformation (BestGene).

#### Transgenes for BiFC technology

Transgenes UAS- pkaC1-VenusC, UAS-pkaR2-VenusC, and UAS-VenusN-SGG were constructed in two steps. Firstly, DNA fragments coding for C-terminal 155–238 and N-terminal 1–154 parts of Venus protein were PCR amplified from respectively plasmids UAStHth-VC and UASTsh-VN (gift from S. Merabet). Primers For. 5′*atatCTCGAG*GCCGACAAGCAGAA3′ plus Rev. 5′*tgcgTCTAGA*TTACTtgtACAGCTCGTCC 3′ and for 5′att*CTCGAG*ATGGTGAGCAAGGGAGA3′ plus Rev. 5′aat*TCTAGA*tccaccactacctccGGTGATATAGACGTTGTGG3′ were used, respectively. Those primers include XhoI and XbaI restriction sites to allow the insertion in the new plasmid, and when necessary, a linker of five amino acids GGSGG were added to separate the Venus fragment from the protein of interest, as well as bases to keep the frame with SGG coding sequence. Each Venus moiety was inserted in a plasmid containing UAS sequence (pJFRC-MUH, addgene 26213) via XhoI and XbaI sites. Secondly, the entire coding sequence of pkaC1, pkaR2, and SGG were PCR amplified from, respectively, FMO06689, FMOO13004, and LD44595 (DGRC clones, Indiana University) and consequently cloned using the Sequence and Ligation Independent Cloning (SLIC) method (*137*) into pJFRC-MUH-VenusC for PkaC1 coding sequences and pJFRC-MUH-VenusN for SGG coding sequence. PkaR2 sequence were cloned into pJFRC-MUH-VenusC using classical cloning technics. Primers For. 5′ATACAAGAAGAGAACTCTGAATAGATCTGCGGCCGCCAGTCGACATGGGCAACAAC3′ and Rev. 5′GCCTTGATGCCGTTCTTCTGCTTGTCGGCCTCGAGTCCACCACTACCTCCGAATTCA 3′ were designed for pkaC1, For. 5′*ATTgcggccgc*ATGTCGAGCGATTCGAGTCG3′ and Rev. 5′AA*gtcgac*tccaccactacctccCAAATTCGTATTACGGCGG3′ for pkaR2, For. 5′ CTATATCACCggaggtagtggtggaTCTAGAcacggtcacgctttacagtc 3′ and Rev. 5′AAGTAAGGTTCCTTCACAAAGATCCtctagaTTTTTTTTTTTTTTTTTTACT3′ for SGG.

BestGene did the transgenesis using the PhiC31 integrase system. UAS-pkaC1-VenusC was inserted in the attP40 docking site 2 L (25C6), UAS-pkaR2-VenusC, and UAS-VenusN-SGG were inserted in the attP2 docking site 3 L (68A4).

### Behavioral experiments

All behavioral experiments were performed with adult flies aged between 3 and 6 days. These flies were raised at 25°C and in 12:12 LD in mixed-sex groups. The activity-rest rhythm was recorded in *Drosophila* Activity Monitor (DAM2 5 mm; TriKinetics) as previously described. For the bigger *D. mercatorum* flies, 7-mm DAM2 tubes were used. 25°C temperature, 12-hour photoperiod, and 500 to 1000 lux (white) light intensity were used for all standard laboratory equinox experiments, unless otherwise mentioned (e.g., for TrpA1 activation). Summer-like conditions were imposed by a constant 28°C temperature and 16-hour photoperiod. Adult *D. melanogaster* flies dwell on fallen plant material in fruit orchards, woodlands, and forests (*138*, *139*). From boreal to subtropical zones, seasonal changes in foliage lead to a dense canopy on summer days and lower light flux at the understorey of fruit orchards and woodlands is a common feature (*140*–*143*). Consequently, flies are paradoxically exposed to lower light intensity in summer months compared to spring and autumn days (*144*–*146*). Furthermore, outside the tropics, twilights marked by low- intensity light availability are substantially longer during summer (*21*), strengthening the association of temperate summer twilights with low light for the crepuscular flies. In light of these, we used lower light intensity (10 to 50 lux, using gray neutral density filters) for summer-like conditions.

For RD experiments, red light was administered by 620- to 650-nm light-emitting diodes (LEDs, Lunartec, Pearl diffusion). USB200 (Ocean Optics) spectrometer was used to measure light spectra and irradiance. For TrpA1 experiments, temperature of the Percival incubators was changed by software control either in 1°C step/hour or directly, and temperature values were cross-checked with independent loggers. Data were analyzed using Faas software, which is derived from Brandeis Rhythm Package. Faas is compatible on Apple Macintosh OSX computers and is freely available (see https://neuropsi.cnrs.fr/en/departments/cnn/group-leader-francois-rouyer/). Activity data are presented as an average of 5 to 6 days and of *n* flies leaving the first 2 to 3 days of the experiment out. On the average activity profiles, each gray/white bar denotes the mean activity levels in a 0.5-hour interval during the light phase, and each black bar denotes the mean activity levels in a 0.5-hour interval during the dark phase of the LD cycle. The dot on each bar shows the SEM. For the LL experiments, rhythmic flies were defined by autocorrelation (RI jitter = 5 bins and maximum lag = 144 bins) function of Faas. To calculate the width of the evening peak, we followed criteria described in (*147*) for defining the onset of an activity peak. For optogenetic experiments, 1- to 2-day-old flies were starved overnight and fed 400 μM all-trans Retinal (ATR in 5% sucrose and 2% LMP agar) for 3 days before starting the experiment during which they continued to feed on a new batch of ATR-laced food. For video analysis of CsChrimson activation, Noldus suite was used and EthoVision software was used to produce the location heatmaps.

### Immunostainings

The immunolabelings were performed using whole-mounted adult *Drosophila* brain. Before dissection, particularly in summer-like conditions, the flies were first placed in TriKinetics monitors to assess their behavior. Brains were dissected in cold phosphate-buffered saline (PBS), fixed in 4% paraformaldehyde (PFA) for 1 hour at room temperature, washed six times in 10-min steps with PBST (PBS containing 0.3% Triton X-100) and permeabilized with PBS containing 1% Triton X-100 before undergoing overnight blocking in 1% bovine serum albumin (BSA) solution. Primary antibodies anti-PER (rabbit; 1:15000), anti-PDF (mouse; 1:20000), anti-GFP (chicken; 1:1000), anti-CLK (guinea-pig; 1:2000), anti-TIM (rat; 1:10000), anti-dsRED (rabbit; 1:2000), anti–S9P-SGG (mouse; 1:20), anti-SGG (mouse; 1:500), anti-SIF*a* (rabbit; 1:5000), anti-hemagglutinin (rat; 1:200), anti-AstC (rabbit; 1:250), and anti- DH31 (guinea-pig; 1:5000) were diluted in PBST containing 0.1% BSA and used for 2 to 3 nights at 4°C. After washing, the brains were treated with secondary antibodies for 2 nights at 4°C, washed, and mounted. Secondary antibodies were Alexa 350–, Alexa 488–, Alexa 547– FP546-, FP568-, and Alexa 647–conjugated goat antibodies (1:1000 to 1:2000 dilution) directed against IgGs of the appropriate species (Invitrogen). To test the effect of PDF on TIM stability on live fly brains (pulse-chase experiment), dissection was undertaken in adult haemolymph like (AHL) solution at room temperature, under red light (>600-nm plastic filter). The brains were incubated in AHL containing 100 μM PDF peptide and kept in an incubator at dark 3.5 hours. Then, the brains were washed, fixed, and processed as above. Fluorescence signals were analyzed with a Zeiss AxioImager Z1 semiconfocal microscope equipped with an AxioCam MRm digital camera and an ApoTome2 module set with an automatically adjustable grid that provided structured illumination. Fiji software was used to quantify fluorescence intensity from digital images. Integrated densities over a defined thresholded area of the axonal arbors of the s-LNvs acquired with a 40× objective were analyzed for quantifying signal in the dorsal projection of the PDF neurons as well as for quantitative GRASP measurement. Use of the wand tool of Fiji yielded equivalent result. For all quantifications from soma, we used the formula: *I* = 100 × (*S*−*B*)/*B* to calculate the fluorescence percentage above background (*S* = mean intensity inside the cell; *B* = the mean intensity of the region adjacent to the positive cell). For studying clock protein oscillations, a 63× objective was used to acquire images.

### Proximity ligation assay

Flies expressing MYC-tagged Sgg10 and GFP-tagged PKA (both regulatory and catalytic subunits were tagged with GFP) pan-clock neuronally by *Clock*^6939^*-Gal4*, were dissected in ice-cold PBS around ZT 2 to 4. The brains were fixed in 4% PFA for 1 hour at room temperature. The whole mounts were treated for regular immunohistochemistry until the addition of the primary antibodies, α-MYC (Novus 9E10; 1:400; rabbit) and α-GFP (Thermo Fisher Scientific, A11120; 1:2000; mouse). After 48 hours, the brains were washed and then incubated with the anti-rabbit and anti-mouse secondary antibodies conjugated to PLUS and MINUS PLA probes [see (*148*)]. T4 ligation and rolling circle amplification of the probes were undertaken using Duolink-provided reagents. The brains were mounted in Prolong-Diamond. The appearance of fluorescent spots signifying successful amplification was detected by a Leica SP8 confocal microscope. No fluorescent spots were detected when the fly expressed MYC-tagged sgg10 alone. Moreover, when the entire procedure was performed without adding the probes, no fluorescence was apparent.

### BiFc assay

BiFC was performed essentially as previously described, with necessary adaptation of the technique for adult brains (*149*, *150*). Transgenes UAS-pkaC1-VenusC, UAS-pkaR2-VenusC, and UAS-VenusN-SGG were constructed as mentioned above. Binary combinations of UAS lines were driven by a pan-clock Gal4 line (*Clock*^6939^*-Gal4*). Brains from 2-to 3-day old adult flies were dissected in PBS and after a quick (10 min) fixation in 4% PFA were immediately imaged in a Leica SP8 confocal microscope using a 20× objective. The quick fixation was necessary for preventing rapid photobleaching. Brains were dissected at ZT2 and ZT14. In the beginning, some of the BiFC brains were additionally costained with anti-PDF antibody. A 515-nm laser was used for Venus excitation, while emission was collected around 528 nm using LasX software interface. Identical parameters of confocal acquisition were applied for every treatment.

### mRNA in-situ hybridization

The RNAscope Multiplex Fluorescent Reagent Kit v2 (ACD Bio) was used on whole-mount *Drosophila* adult brains as previously described (*151*). Dissected brains were fixed, washed, and then treated with 3% hydrogen peroxide (3% H2O2) for 20 min at room temperature (RT). The target retrieval treatment was performed for 2 min at 95°C in 1× Target Retrieval solution, and, subsequently, the brains were treated at room temperature with Protease IV for 30 min, and washed. The probes (*Pdf* probe: catalog no. 457471-C3 and *Pdfr* probe: catalog no. 576571-C2, created by ACD Bio for the present study) were warmed to 40°C and added to the brains incubated with the probe diluent solution after 1:50 dilution. After overnight incubation at 40°C, the brains were washed and incubated with two to three drops of RNAscope Multiplex FL v2 at 40°C for 30 min and rewashed. These steps were repeated with Amp 2 and Amp 3 incubations, followed by C2 and/or C3 treatments. In the last step, the samples were incubated for 30 min at 40°C with Opal 520 (FP1487001KT, 1:2000 dilution) and/or Opal 650 (FP1496001KT, 1:2000 dilution). The brains were washed and incubated overnight at 4°C with anti-PDF or anti–red fluorescent protein antibodies. The brains were imaged with semiconfocal microscope, and quantification of signal intensity was carried out as for immunostainings.

### GCaMP imaging

Adult flies were dissected under ice-cold AHL for PDF bath-application experiments and under ice-cold HL3 for P2X2 experiments was used. The whole-brain explants were placed on 42-mm-diameter coverslips treated with poly-d-lysine and Laminin. Then, the preparation was immersed in oxygenated AHL or HL3. Calcium imaging was performed using a Zeiss Axio Examiner D1 upright microscope with Apochromat 40× W numerical aperture 1.0 immersion lens. GCaMP6s probe was excited (25-ms exposure time) with a Colibri 470-nm LED light source, and images were acquired using AxioCam MRm at 0.5- to 2-Hz sampling rate. Adenosine triphosphate (ATP, 0.5 mM; Sigma-Aldrich Chemical) was used to stimulate the P2X2 channel. When used, 30 μM PDF (PolyPeptide) was added after at least a minute of baseline recording. ATP was dissolved in HL3 solution, whereas PDF was dissolved in 0.1% dimethyl sulfoxide in AHL, essentially as in (*11*). Preincubation with 2 μM TTX was performed for 15 to 20 min, and fresh 2 μM TTX was reapplied immediately before image acquisition [see (*135*)]. To measure baseline GCaMP6s intensity, live brains in room-temperature AHL were imaged immediately after dissection. *xyzt* stacks were acquired for 1 min, and a maximum intensity projection was rendered, which was subsequently treated as a static image. The average fluorescence of all pixels for each time point in a defined region of interest (ROI) was subtracted from the average background fluorescence of an identical size ROI elsewhere in the brain. The resulting pixel fluorescence value for each time point was defined as trace Fb. Changes in fluorescence were computed as %d*F*/*F*_0_ = [(Fb − *F*_0_)/*F*_0_] × 100, where *F*_0_ is defined as the average background-subtracted baseline fluorescence for the 30 to 60 frames preceding the stimulus application. Fiji (ImageJ) software was used for processing and quantifying all images. Maximum GCaMP6s fluorescence change values (Max d*F*/*F*_0_) were determined as the maximum percentage change observed for each trace over the entire duration in a given imaging experiment. Maximum values for each treatment and genotype were averaged to calculate the mean maximum change from baseline.

## Supplementary Materials

### This PDF file includes:

Figs. S1 to S5 Table S1 References

## References

[1] A. Patke, M. W. Young, S. Axelrod, Molecular mechanisms and physiological importance of circadian rhythms. Nat. Rev. Mol. Cell Biol. 21, 67–84 (2020). 31768006 10.1038/s41580-019-0179-2

[2] S. Michel, J. H. Meijer, From clock to functional pacemaker. Eur. J. Neurosci. 51, 482–493 (2020). 30793396 10.1111/ejn.14388 PMC7027845

[3] M. Ahmad, W. Li, D. Top, Integration of circadian clock information in the *Drosophila* circadian neuronal network. J. Biol. Rhythms 36, 203–220 (2021). 33641476 10.1177/0748730421993953 PMC8114447

[4] S. L. Crespo-Flores, A. F. Barber, The *Drosophila* circadian clock circuit is a nonhierarchical network of peptidergic oscillators. Curr. Opin. Insect Sci. 52, 100944 (2022). 35709899 10.1016/j.cois.2022.100944

[5] D. Sidote, J. Majercak, V. Parikh, I. Edery, Differential effects of light and heat on the *Drosophila* circadian clock proteins PER and TIM. Mol. Cell. Biol. 18, 2004–2013 (1998). 9528772 10.1128/mcb.18.4.2004 PMC121430

[6] J. Majercak, D. Sidote, P. E. Hardin, I. Edery, How a circadian clock adapts to seasonal decreases in temperature and day length. Neuron 24, 219–230 (1999). 10677039 10.1016/s0896-6273(00)80834-x

[7] C. Helfrich-Förster, E. Bertolini, P. Menegazzi, Flies as models for circadian clock adaptation to environmental challenges. Eur. J. Neurosci. 51, 166–181 (2020). 30269385 10.1111/ejn.14180 PMC7027873

[8] C. Helfrich-Förster, Light input pathways to the circadian clock of insects with an emphasis on the fruit fly *Drosophila* melanogaster. J. Comp. Physiol. A Neuroethol. Sens. Neural Behav. Physiol. 206, 259–272 (2020). 31691095 10.1007/s00359-019-01379-5 PMC7069913

[9] R. George, R. Stanewsky, Peripheral sensory organs contribute to temperature synchronization of the circadian clock in *Drosophila* melanogaster. Front. Physiol. 12, 622545 (2021). 33603678 10.3389/fphys.2021.622545 PMC7884628

[10] Y. Zhang, Y. Liu, D. Bilodeau-Wentworth, P. E. Hardin, P. Emery, Light and temperature control the contribution of specific DN1 neurons to *Drosophila* circadian behavior. Curr. Biol. 20, 600–605 (2010). 20362449 10.1016/j.cub.2010.02.044 PMC2862552

[11] A. Chatterjee, A. Lamaze, J. De, W. Mena, E. Chélot, B. Martin, P. Hardin, S. Kadener, P. Emery, F. Rouyer, Reconfiguration of a multi-oscillator network by light in the *Drosophila* circadian clock. Curr. Biol. 28, 2007–2017 (2018). 29910074 10.1016/j.cub.2018.04.064 PMC6039274

[12] M. Schlichting, P. Weidner, M. Diaz, P. Menegazzi, E. Dalla Benetta, C. Helfrich-Förster, M. Rosbash, Light-mediated circuit switching in the *Drosophila* neuronal clock network. Curr. Biol. 29, 3266–3276.e3 (2019). 31564496 10.1016/j.cub.2019.08.033

[13] O. T. Shafer, A. C. Keene, The regulation of *Drosophila* sleep. Curr. Biol. 31, R38–R49 (2021). 33434488 10.1016/j.cub.2020.10.082

[14] D. Monsivais, A. Ghosh, K. Bhattacharya, R. I. M. Dunbar, K. Kaski, Tracking urban human activity from mobile phone calling patterns. PLOS Comput. Biol. 13, e1005824 (2017). 29161270 10.1371/journal.pcbi.1005824 PMC5697809

[15] T. Roenneberg, Twitter as a means to study temporal behaviour. Curr. Biol. 27, R830–R832 (2017). 28898641 10.1016/j.cub.2017.08.005

[16] B. Grima, E. Chélot, R. Xia, F. Rouyer, Morning and evening peaks of activity rely on different clock neurons of the *Drosophila* brain. Nature 431, 869–873 (2004). 15483616 10.1038/nature02935

[17] D. Stoleru, P. Peng, J. Agosto, M. Rosbash, Coupled oscillators control morning and evening locomotor behavior of *Drosophila*. Nature 431, 862–868 (2004). 15483615 10.1038/nature02926

[18] D. Rieger, O. T. Shafer, K. Tomioka, C. Helfrich-Forster, Functional analysis of circadian pacemaker neurons in *Drosophila* melanogaster. J. Neurosci. 26, 2531–2543 (2006). 16510731 10.1523/JNEUROSCI.1234-05.2006 PMC6793667

[19] X. Liang, T. E. Holy, P. H. Taghert, Synchronous *Drosophila* circadian pacemakers display nonsynchronous Ca^2+^ rhythms in vivo. Science 351, 976–981 (2016). 26917772 10.1126/science.aad3997 PMC4836443

[20] E. Bünning, Circadian rhythms and the time measurement in photoperiodism. Cold Spring Harb. Symp. Quant. Biol. 25, 249–256 (1960).

[21] R. A. Hut, S. Paolucci, R. Dor, C. P. Kyriacou, S. Daan, Latitudinal clines: An evolutionary view on biological rhythms. Proc. Biol. Sci. 280, 20130433 (2013). 23825204 10.1098/rspb.2013.0433 PMC3712436

[22] D. Saunders, Insect photoperiodism: Bunning’s hypothesis, the history and development of an idea. Eur. J. Entomol. 118, 1–13 (2021).

[23] H. T. Vanderleest, T. Houben, S. Michel, T. Deboer, H. Albus, M. J. Vansteensel, G. D. Block, J. H. Meijer, Seasonal encoding by the circadian pacemaker of the SCN. Curr. Biol. 17, 468–473 (2007). 17320387 10.1016/j.cub.2007.01.048

[24] E. A. Lucassen, H. C. van Diepen, T. Houben, S. Michel, C. S. Colwell, J. H. Meijer, Role of vasoactive intestinal peptide in seasonal encoding by the suprachiasmatic nucleus clock. Eur. J. Neurosci. 35, 1466–1474 (2012). 22512278 10.1111/j.1460-9568.2012.08054.x

[25] A. Porcu, A. Nilsson, S. Booreddy, S. A. Barnes, D. K. Welsh, D. Dulcis, Seasonal changes in day length induce multisynaptic neurotransmitter switching to regulate hypothalamic network activity and behavior. Sci. Adv. 8, eabn9867 (2022). 36054362 10.1126/sciadv.abn9867 PMC10848959

[26] S. Michel, L. Kervezee, One seasonal clock fits all? J. Comp. Physiol. A Neuroethol. Sens. Neural Behav. Physiol. 210, 641–647 (2024). 37947808 10.1007/s00359-023-01680-4 PMC11226558

[27] P. Menegazzi, S. Vanin, T. Yoshii, D. Rieger, C. Hermann, V. Dusik, C. P. Kyriacou, C. Helfrich- Forster, R. Costa, Drosophila clock neurons under natural conditions. J. Biol. Rhythms 28, 3–14 (2013). 23382587 10.1177/0748730412471303

[28] K. Tomioka, M. Yukizane, A specific area of the compound eye in the cricket *Gryllus* bimaculatus sends photic information to the circadian pacemaker in the contralateral optic lobe. J. Comp. Physiol. A 180, 63–70 (1997).

[29] M. Picot, P. Cusumano, A. Klarsfeld, R. Ueda, F. Rouyer, Light activates output from evening neurons and inhibits output from morning neurons in the *Drosophila* circadian clock. PLOS Biol. 5, e315 (2007). 18044989 10.1371/journal.pbio.0050315 PMC2229858

[30] D. Stoleru, P. Nawathean, L. Fernandez Mde, J. S. Menet, M. F. Ceriani, M. Rosbash, The *Drosophila* circadian network is a seasonal timer. Cell 129, 207–219 (2007). 17418796 10.1016/j.cell.2007.02.038

[31] O. T. Shafer, J. D. Levine, J. W. Truman, J. C. Hall, Flies by night: Effects of changing day length on *Drosophila*’s circadian clock. Curr. Biol. 14, 424–432 (2004). 15028219 10.1016/j.cub.2004.02.038

[32] W. Bywalez, P. Menegazzi, D. Rieger, B. Schmid, C. Helfrich-Forster, T. Yoshii, The dual-oscillator system of *Drosophila melanogaster* under natural-like temperature cycles. Chronobiol. Int. 29, 395–407 (2012). 22489637 10.3109/07420528.2012.668505

[33] C. Pittendrigh, S. Daan, A functional analysis of circadian pacemakers in nocturnal rodents. IV. Entrainment: Pacemaker as clock. J. Comp. Physiol. 106, 291–331 (1976).

[34] S. Vanin, S. Bhutani, S. Montelli, P. Menegazzi, E. W. Green, M. Pegoraro, F. Sandrelli, R. Costa, C. P. Kyriacou, Unexpected features of *Drosophila* circadian behavioural rhythms under natural conditions. Nature 484, 371–375 (2012). 22495312 10.1038/nature10991

[35] P. Menegazzi, T. Yoshii, C. Helfrich-Forster, Laboratory versus nature: The two sides of the *Drosophila* circadian clock. J. Biol. Rhythms 27, 433–442 (2012). 23223369 10.1177/0748730412463181

[36] J. De, V. Varma, S. Saha, V. Sheeba, V. K. Sharma, Significance of activity peaks in fruit flies, *Drosophila melanogaster*, under seminatural conditions. Proc. Natl. Acad. Sci. U.S.A. 110, 8984–8989 (2013). 23671102 10.1073/pnas.1220960110 PMC3670394

[37] E. W. Green, E. K. O’Callaghan, C. N. Hansen, S. Bastianello, S. Bhutani, S. Vanin, J. D. Armstrong, R. Costa, C. P. Kyriacou, *Drosophila* circadian rhythms in seminatural environments: Summer afternoon component is not an artifact and requires TrpA1 channels. Proc. Natl. Acad. Sci. U.S.A. 112, 8702–8707 (2015). 26124142 10.1073/pnas.1506093112 PMC4507215

[38] H. Kauranen, P. Menegazzi, R. Costa, C. Helfrich-Forster, A. Kankainen, A. Hoikkala, Flies in the north: Locomotor behavior and clock neuron organization of *Drosophila montana*. J. Biol. Rhythms 27, 377–387 (2012). 23010660 10.1177/0748730412455916

[39] P. Menegazzi, E. Dalla Benetta, M. Beauchamp, M. Schlichting, I. Steffan-Dewenter, C. Helfrich-Förster, Adaptation of circadian neuronal network to photoperiod in high-Latitude european *Drosophilids*. Curr. Biol. 27, 833–839 (2017). 28262491 10.1016/j.cub.2017.01.036

[40] E. Bertolini, F. K. Schubert, D. Zanini, H. Sehadová, C. Helfrich-Förster, P. Menegazzi, Life at high latitudes does not require circadian behavioral rhythmicity under constant darkness. Curr. Biol. 29, 3928–3936.e3 (2019). 31679928 10.1016/j.cub.2019.09.032

[41] D. Nagy, P. Cusumano, G. Andreatta, A. M. Anduaga, C. Hermann-Luibl, N. Reinhard, J. Gesto, C. Wegener, G. Mazzotta, E. Rosato, C. P. Kyriacou, C. Helfrich-Förster, R. Costa, Peptidergic signaling from clock neurons regulates reproductive dormancy in *Drosophila melanogaster*. PLOS Genet. 15, e1008158 (2019). 31194738 10.1371/journal.pgen.1008158 PMC6592559

[42] S. Hidalgo, M. Anguiano, C. A. Tabuloc, J. C. Chiu, Seasonal cues act through the circadian clock and pigment-dispersing factor to control EYES ABSENT and downstream physiological changes. Curr. Biol. 33, 675–687.e5 (2023). 36708710 10.1016/j.cub.2023.01.006 PMC9992282

[43] S. Hidalgo, J. C. Chiu, Integration of photoperiodic and temperature cues by the circadian clock to regulate insect seasonal adaptations. J. Comp. Physiol. A Neuroethol. Sens. Neural Behav. Physiol. 210, 585–599 (2024). 37584703 10.1007/s00359-023-01667-1 PMC11057393

[44] C. Helfrich-Förster, Neuropeptidergic regulation of insect diapause by the circadian clock. Curr. Opin. Insect Sci. 63, 101198 (2024). 38588944 10.1016/j.cois.2024.101198

[45] A. Abrieux, Y. Xue, Y. Cai, K. M. Lewald, H. N. Nguyen, Y. Zhang, J. C. Chiu, EYES ABSENT and TIMELESS integrate photoperiodic and temperature cues to regulate seasonal physiology in *Drosophila*. Proc. Natl. Acad. Sci. U.S.A. 117, 15293–15304 (2020). 32541062 10.1073/pnas.2004262117 PMC7334534

[46] S. C. Renn, J. H. Park, M. Rosbash, J. C. Hall, P. H. Taghert, A pdf neuropeptide gene mutation and ablation of PDF neurons each cause severe abnormalities of behavioral circadian rhythms in *Drosophila*. Cell 99, 791–802 (1999). 10619432 10.1016/s0092-8674(00)81676-1

[47] T. Yoshii, C. Wulbeck, H. Sehadova, S. Veleri, D. Bichler, R. Stanewsky, C. Helfrich-Forster, The neuropeptide pigment-dispersing factor adjusts period and phase of *Drosophila*’s clock. J. Neurosci. 29, 2597–2610 (2009). 19244536 10.1523/JNEUROSCI.5439-08.2009 PMC6666242

[48] P. Cusumano, A. Klarsfeld, E. Chélot, M. Picot, B. Richier, F. Rouyer, PDF-modulated visual inputs and cryptochrome define diurnal behavior in *Drosophila*. Nat. Neurosci. 12, 1431–1437 (2009). 19820704 10.1038/nn.2429

[49] B. C. Lear, L. Zhang, R. Allada, The neuropeptide PDF acts directly on evening pacemaker neurons to regulate multiple features of circadian behavior. PLOS Biol. 7, e1000154 (2009). 19621061 10.1371/journal.pbio.1000154 PMC2702683

[50] L. Zhang, B. C. Lear, A. Seluzicki, R. Allada, The CRYPTOCHROME photoreceptor gates PDF neuropeptide signaling to set circadian network hierarchy in *Drosophila*. Curr. Biol. 19, 2050–2055 (2009). 19913424 10.1016/j.cub.2009.10.058 PMC2805779

[51] M. Schlichting, P. Menegazzi, K. R. Lelito, Z. Yao, E. Buhl, E. Dalla Benetta, A. Bahle, J. Denike, J. J. Hodge, C. Helfrich-Förster, O. T. Shafer, A neural network underlying circadian entrainment and photoperiodic adjustment of cleep and activity in *Drosophila*. J. Neurosci. 36, 9084–9096 (2016). 27581451 10.1523/JNEUROSCI.0992-16.2016 PMC5005721

[52] K. M. Vaze, C. Helfrich-Förster, The neuropeptide PDF is crucial for delaying the phase of *Drosophila*’s evening neurons under long zeitgeber periods. J. Biol. Rhythms 36, 442–460 (2021). 34428956 10.1177/07487304211032336 PMC8442139

[53] W. Bachleitner, L. Kempinger, C. Wulbeck, D. Rieger, C. Helfrich-Forster, Moonlight shifts the endogenous clock of *Drosophila* melanogaster. Proc. Natl. Acad. Sci. U.S.A. 104, 3538–3543 (2007). 17307880 10.1073/pnas.0606870104 PMC1805525

[54] D. Rieger, C. Fraunholz, J. Popp, D. Bichler, R. Dittmann, C. Helfrich-Forster, The fruit fly *Drosophila melanogaster* favors dim light and times its activity peaks to early dawn and late dusk. J. Biol. Rhythms 22, 387–399 (2007). 17876060 10.1177/0748730407306198

[55] S. Lazopulo, A. Lazopulo, J. D. Baker, S. Syed, Daytime colour preference in *Drosophila* depends on the circadian clock and TRP channels. Nature 574, 108–111 (2019). 31534223 10.1038/s41586-019-1571-y

[56] G. T. Meyerhof, S. Easwaran, A. E. Bontempo, C. Montell, D. J. Montell, Altered circadian rhythm, sleep, and rhodopsin 7-dependent shade preference during diapause in *Drosophila melanogaster*. Proc. Natl. Acad. Sci. U.S.A. 121, e2400964121 (2024). 38917005 10.1073/pnas.2400964121 PMC11228485

[57] H. Kauranen, O. Ala-Honkola, M. Kankare, A. Hoikkala, Circadian clock of *Drosophila montana* is adapted to high variation in summer day lengths and temperatures prevailing at high latitudes. J. Insect Physiol. 89, 9–18 (2016). 26993661 10.1016/j.jinsphys.2016.03.005

[58] M. Beauchamp, E. Bertolini, P. Deppisch, J. Steubing, P. Menegazzi, C. Helfrich-Förster, Closely related fruit fly species living at different latitudes diverge in their circadian clock anatomy and rhythmic behavior. J. Biol. Rhythms 33, 602–613 (2018). 30203704 10.1177/0748730418798096

[59] K. H. Low, C. Lim, H. W. Ko, I. Edery, Natural variation in the splice site strength of a clock gene and species-specific thermal adaptation. Neuron 60, 1054–1067 (2008). 19109911 10.1016/j.neuron.2008.10.048 PMC2631419

[60] A. Kopp, O. Barmina, Evolutionary history of the *Drosophila* bipectinata species complex. Genet. Res. 85, 23–46 (2005). 16089034 10.1017/s0016672305007317

[61] A. Seluzicki, M. Flourakis, E. Kula-Eversole, L. Zhang, V. Kilman, R. Allada, Dual PDF signaling pathways reset clocks via TIMELESS and acutely excite target neurons to control circadian behavior. PLOS Biol. 12, e1001810 (2014). 24643294 10.1371/journal.pbio.1001810 PMC3958333

[62] V. Sabado, L. Vienne, J. M. Nunes, M. Rosbash, E. Nagoshi, Fluorescence circadian imaging reveals a PDF-dependent transcriptional regulation of the *Drosophila* molecular clock. Sci. Rep. 7, 41560 (2017). 28134281 10.1038/srep41560 PMC5278502

[63] Y. Li, F. Guo, J. Shen, M. Rosbash, PDF and cAMP enhance PER stability in *Drosophila* clock neurons. Proc. Natl. Acad. Sci. U.S.A. 111, E1284–E1290 (2014). 24707054 10.1073/pnas.1402562111 PMC3977231

[64] S. Martinek, S. Inonog, A. S. Manoukian, M. W. Young, A role for the segment polarity gene shaggy/GSK-3 in the *Drosophila* circadian clock. Cell 105, 769–779 (2001). 11440719 10.1016/s0092-8674(01)00383-x

[65] D. Top, E. Harms, S. Syed, E. L. Adams, L. Saez, GSK-3 and CK2 kinases converge on timeless to regulate the master clock. Cell Rep. 16, 357–367 (2016). 27346344 10.1016/j.celrep.2016.06.005 PMC4945451

[66] X. Fang, S. X. Yu, Y. Lu, R. C. J. Bast, J. R. Woodgett, G. B. Mills, Phosphorylation and inactivation of glycogen synthase kinase 3 by protein kinase A. Proc. Natl. Acad. Sci. U.S.A. 97, 11960–11965 (2000). 11035810 10.1073/pnas.220413597 PMC17277

[67] M. Li, X. Wang, M. K. Meintzer, T. Laessig, M. J. Birnbaum, K. A. Heidenreich, Cyclic AMP promotes neuronal survival by phosphorylation of glycogen synthase kinase 3beta. Mol. Cell. Biol. 20, 9356–9363 (2000). 11094086 10.1128/mcb.20.24.9356-9363.2000 PMC102192

[68] D. Ma, D. Przybylski, K. C. Abruzzi, M. Schlichting, Q. Li, X. Long, M. Rosbash, A transcriptomic taxonomy of *Drosophila* circadian neurons around the clock. eLife 10, e63056 (2021). 33438579 10.7554/eLife.63056 PMC7837698

[69] A. Lamaze, R. Stanewsky, DN1p or the “fluffy” cerberus of clock outputs. Front. Physiol. 10, 1540 (2019). 31969832 10.3389/fphys.2019.01540 PMC6960142

[70] A. Murad, M. Emery-Le, P. Emery, A subset of dorsal neurons modulates circadian behavior and light responses in *Drosophila*. Neuron 53, 689–701 (2007). 17329209 10.1016/j.neuron.2007.01.034 PMC1852515

[71] M. Kunst, M. E. Hughes, D. Raccuglia, M. Felix, M. Li, G. Barnett, J. Duah, M. N. Nitabach, Calcitonin gene-related peptide neurons mediate sleep-specific circadian output in *Drosophila*. Curr. Biol. 24, 2652–2664 (2014). 25455031 10.1016/j.cub.2014.09.077 PMC4255360

[72] F. Guo, J. Yu, H. J. Jung, K. C. Abruzzi, W. Luo, L. C. Griffith, M. Rosbash, Circadian neuron feedback controls the *Drosophila* sleep-activity profile. Nature 536, 292–297 (2016). 27479324 10.1038/nature19097 PMC5247284

[73] F. Guo, M. Holla, M. M. Díaz, M. Rosbash, A circadian output circuit controls sleep-wake arousal in *Drosophila*. Neuron 100, 624–635.e4 (2018). 30269992 10.1016/j.neuron.2018.09.002

[74] A. Lamaze, P. Krätschmer, K. F. Chen, S. Lowe, J. E. C. Jepson, A wake-promoting circadian output circuit in *Drosophila*. Curr. Biol. 28, 3098–3105.e3 (2018). 30270186 10.1016/j.cub.2018.07.024

[75] F. Guo, X. Chen, M. Rosbash, Temporal calcium profiling of specific circadian neurons in freely moving flies. Proc. Natl. Acad. Sci. U.S.A. 114, E8780–E8787 (2017). 28973886 10.1073/pnas.1706608114 PMC5642695

[76] R. A. Baines, J. P. Uhler, A. Thompson, S. T. Sweeney, M. Bate, Altered electrical properties in *Drosophila* neurons developing without synaptic transmission. J. Neurosci. 21, 1523–1531 (2001). 11222642 10.1523/JNEUROSCI.21-05-01523.2001 PMC6762927

[77] F. N. Hamada, M. Rosenzweig, K. Kang, S. R. Pulver, A. Ghezzi, T. J. Jegla, P. A. Garrity, An internal thermal sensor controlling temperature preference in *Drosophila*. Nature 454, 217–220 (2008). 18548007 10.1038/nature07001 PMC2730888

[78] A. Dawydow, R. Gueta, D. Ljaschenko, S. Ullrich, M. Hermann, N. Ehmann, S. Gao, A. Fiala, T. Langenhan, G. Nagel, R. J. Kittel, Channelrhodopsin-2-XXL, a powerful optogenetic tool for low-light applications. Proc. Natl. Acad. Sci. U.S.A. 111, 13972–13977 (2014). 25201989 10.1073/pnas.1408269111 PMC4183338

[79] X. Liang, T. E. Holy, P. H. Taghert, A series of suppressive signals within the *Drosophila* circadian neural circuit generates sequential daily outputs. Neuron 94, 1173–1189.e4 (2017). 28552314 10.1016/j.neuron.2017.05.007 PMC5502710

[80] M. Talay, E. B. Richman, N. J. Snell, G. G. Hartmann, J. D. Fisher, A. Sorkaç, J. F. Santoyo, C. Chou-Freed, N. Nair, M. Johnson, J. R. Szymanski, G. Barnea, Transsynaptic mapping of second-order taste neurons in flies by trans-Tango. Neuron 96, 783–795.e4 (2017). 29107518 10.1016/j.neuron.2017.10.011 PMC5693608

[81] Y. Zhang, Y. Zhou, X. Zhang, L. Wang, Y. Zhong, Clock neurons gate memory extinction in *Drosophila*. Curr. Biol. 31, 1337–1343.e4 (2021). 33545046 10.1016/j.cub.2021.01.008

[82] L. Bai, Y. Lee, C. T. Hsu, J. A. Williams, D. Cavanaugh, X. Zheng, C. Stein, P. Haynes, H. Wang, D. H. Gutmann, A. Sehgal, A conserved circadian function for the neurofibromatosis 1 gene. Cell Rep. 22, 3416–3426 (2018). 29590612 10.1016/j.celrep.2018.03.014 PMC5898822

[83] D. J. Cavanaugh, J. D. Geratowski, J. R. Wooltorton, J. M. Spaethling, C. E. Hector, X. Zheng, E. C. Johnson, J. H. Eberwine, A. Sehgal, Identification of a circadian output circuit for rest:activity rhythms in *Drosophila*. Cell 157, 689–701 (2014). 24766812 10.1016/j.cell.2014.02.024 PMC4003459

[84] S. Park, J. Y. Sonn, Y. Oh, C. Lim, J. Choe, SIFamide and SIFamide receptor defines a novel neuropeptide signaling to promote sleep in *Drosophila*. Mol. Cells 37, 295–301 (2014). 24658384 10.14348/molcells.2014.2371 PMC4012077

[85] H. Huang, D. R. Possidente, C. G. Vecsey, Optogenetic activation of SIFamide (SIFa) neurons induces a complex sleep-promoting effect in the fruit fly *Drosophila melanogaster*. Physiol. Behav. 239, 113507 (2021). 34175361 10.1016/j.physbeh.2021.113507 PMC8440390

[86] N. Reinhard, A. Fukuda, G. Manoli, E. Derksen, A. Saito, G. Möller, M. Sekiguchi, D. Rieger, C. Helfrich-Förster, T. Yoshii, M. Zandawala, Synaptic connectome of the *Drosophila* circadian clock. Nat. Commun. 15, 10392 (2024). 39638801 10.1038/s41467-024-54694-0 PMC11621569

[87] L. K. Scheffer, C. S. Xu, M. Januszewski, Z. Lu, S. Y. Takemura, K. J. Hayworth, G. B. Huang, K. Shinomiya, J. Maitlin-Shepard, S. Berg, J. Clements, P. M. Hubbard, W. T. Katz, L. Umayam, T. Zhao, D. Ackerman, T. Blakely, J. Bogovic, T. Dolafi, D. Kainmueller, T. Kawase, K. A. Khairy, L. Leavitt, P. H. Li, L. Lindsey, N. Neubarth, D. J. Olbris, H. Otsuna, E. T. Trautman, M. Ito, A. S. Bates, J. Goldammer, T. Wolff, R. Svirskas, P. Schlegel, E. Neace, C. J. Knecht, C. X. Alvarado, D. A. Bailey, S. Ballinger, J. A. Borycz, B. S. Canino, N. Cheatham, M. Cook, M. Dreher, O. Duclos, B. Eubanks, K. Fairbanks, S. Finley, N. Forknall, A. Francis, G. P. Hopkins, E. M. Joyce, S. Kim, N. A. Kirk, J. Kovalyak, S. A. Lauchie, A. Lohff, C. Maldonado, E. A. Manley, S. McLin, C. Mooney, M. Ndama, O. Ogundeyi, N. Okeoma, C. Ordish, N. Padilla, C. M. Patrick, T. Paterson, E. E. Phillips, E. M. Phillips, N. Rampally, C. Ribeiro, M. K. Robertson, J. T. Rymer, S. M. Ryan, M. Sammons, A. K. Scott, A. L. Scott, A. Shinomiya, C. Smith, K. Smith, N. L. Smith, M. A. Sobeski, A. Suleiman, J. Swift, S. Takemura, I. Talebi, D. Tarnogorska, E. Tenshaw, T. Tokhi, J. J. Walsh, T. Yang, J. A. Horne, F. Li, R. Parekh, P. K. Rivlin, V. Jayaraman, M. Costa, G. S. Jefferis, K. Ito, S. Saalfeld, R. George, I. A. Meinertzhagen, G. M. Rubin, H. F. Hess, V. Jain, S. M. Plaza, A connectome and analysis of the adult *Drosophila* central brain. eLife 9, e57443 (2020). 32880371 10.7554/eLife.57443 PMC7546738

[88] M. P. Shahandeh, L. Abuin, L. Lescuyer De Decker, J. Cergneux, R. Koch, E. Nagoshi, R. Benton, Circadian plasticity evolves through regulatory changes in a neuropeptide gene. Nature 635, 951–959 (2024). 39415010 10.1038/s41586-024-08056-x PMC11602725

[89] S. Bhutani, Natural entrainment of the Drosophila melanogaster circadian clock. PhD thesis. University of Leicester UK (2009).

[90] J. H. Park, J. C. Hall, Isolation and chronobiological analysis of a neuropeptide pigment- dispersing factor gene in *Drosophila melanogaster*. J. Biol. Rhythms 13, 219–228 (1998). 9615286 10.1177/074873098129000066

[91] K. C. Abruzzi, A. Zadina, W. Luo, E. Wiyanto, R. Rahman, F. Guo, O. Shafer, M. Rosbash, RNA- seq analysis of *Drosophila* clock and non-clock neurons reveals neuron-specific cycling and novel candidate neuropeptides. PLOS Genet. 13, e1006613 (2017). 28182648 10.1371/journal.pgen.1006613 PMC5325595

[92] A. A. Hoffmann, Physiological climatic limits in *Drosophila*: Patterns and implications. J. Exp. Biol. 213, 870–880 (2010). 20190112 10.1242/jeb.037630

[93] Y. Yang, I. Edery, Daywake, an anti-siesta gene linked to a splicing-based thermostat from an adjoining clock gene. Curr. Biol. 29, 1728–1734.e4 (2019). 31080079 10.1016/j.cub.2019.04.039 PMC6586486

[94] B. H. Collins, E. Rosato, C. P. Kyriacou, Seasonal behavior in *Drosophila melanogaster* requires the photoreceptors, the circadian clock, and phospholipase C. Proc. Natl. Acad. Sci. U.S.A. 101, 1945–1950 (2004). 14766972 10.1073/pnas.0308240100 PMC357032

[95] J. Majercak, W. F. Chen, I. Edery, Splicing of the period gene 3’-terminal intron is regulated by light, circadian clock factors, and phospholipase C. Mol. Cell. Biol. 24, 3359–3372 (2004). 15060157 10.1128/MCB.24.8.3359-3372.2004 PMC381688

[96] C. Breda, E. Rosato, C. P. Kyriacou, Norpa signalling and the seasonal circadian locomotor phenotype in *Drosophila*. Biology 9, 130 (2020). 32560221 10.3390/biology9060130 PMC7345481

[97] I. Edery, Did a small thermosensitive intron contribute to the temperate adaptation of *Drosophila melanogaster*? Med. Res. Arch. 11, 4624 (2023). 38144715 10.18103/mra.v11i11.4624 PMC10745283

[98] F. Guo, I. Cerullo, X. Chen, M. Rosbash, PDF neuron firing phase-shifts key circadian activity neurons in *Drosophila*. eLife 3, e02780 (2014). 24939987 10.7554/eLife.02780 PMC4092873

[99] S. Mezan, J. D. Feuz, B. Deplancke, S. Kadener, PDF signaling is an integral part of the *Drosophila* circadian molecular oscillator. Cell Rep. 17, 708–719 (2016). 27732848 10.1016/j.celrep.2016.09.048 PMC5081397

[100] J. C. Chiu, H. W. Ko, I. Edery, NEMO/NLK phosphorylates PERIOD to initiate a time-delay phosphorylation circuit that sets circadian clock speed. Cell 145, 357–370 (2011). 21514639 10.1016/j.cell.2011.04.002 PMC3092788

[101] L. B. Duvall, P. H. Taghert, E and M circadian pacemaker neurons use different PDF receptor signalosome components in drosophila. J. Biol. Rhythms 28, 239–248 (2013). 23929551 10.1177/0748730413497179 PMC3980953

[102] D. Top, M. W. Young, Coordination between differentially regulated circadian clocks generates rhythmic behavior. Cold Spring Harb. Perspect. Biol. 10, a033589 (2018). 28893860 10.1101/cshperspect.a033589 PMC6028074

[103] X. Zheng, A. Sehgal, AKT and TOR signaling set the pace of the circadian pacemaker. Curr. Biol. 20, 1203–1208 (2010). 20619819 10.1016/j.cub.2010.05.027 PMC3165196

[104] Q. Yuan, F. Lin, X. Zheng, A. Sehgal, Serotonin modulates circadian entrainment in *Drosophila*. Neuron 47, 115–127 (2005). 15996552 10.1016/j.neuron.2005.05.027

[105] E. Tauber, M. Zordan, F. Sandrelli, M. Pegoraro, N. Osterwalder, C. Breda, A. Daga, A. Selmin, K. Monger, C. Benna, E. Rosato, C. P. Kyriacou, R. Costa, Natural selection favors a newly derived timeless allele in *Drosophila melanogaster*. Science 316, 1895–1898 (2007). 17600215 10.1126/science.1138412

[106] F. Sandrelli, E. Tauber, M. Pegoraro, G. Mazzotta, P. Cisotto, J. Landskron, R. Stanewsky, A. Piccin, E. Rosato, M. Zordan, R. Costa, C. P. Kyriacou, A molecular basis for natural selection at the timeless locus in *Drosophila melanogaster*. Science 316, 1898–1900 (2007). 17600216 10.1126/science.1138426

[107] P. Deppisch, J. M. Prutscher, M. Pegoraro, E. Tauber, C. Wegener, C. Helfrich-Förster, Adaptation of *Drosophila melanogaster* to long photoperiods of high-latitude summers is facilitated by the ls-timeless allele. J. Biol. Rhythms 37, 185–201 (2022). 35301885 10.1177/07487304221082448 PMC9008550

[108] A. Lamaze, C. Chen, S. Leleux, M. Xu, R. George, R. Stanewsky, A natural timeless polymorphism allowing circadian clock synchronization in “white nights”. Nat. Commun. 13, 1724 (2022). 35361756 10.1038/s41467-022-29293-6 PMC8971440

[109] K. M. Vaze, G. Manoli, C. Helfrich-Förster, *Drosophila ezoana* uses morning and evening oscillators to adjust its rhythmic activity to different daylengths but only the morning oscillator to measure night length for photoperiodic responses. J. Comp. Physiol. A Neuroethol. Sens. Neural Behav. Physiol. 210, 535–548 (2024). 37329349 10.1007/s00359-023-01646-6 PMC11226516

[110] C. E. Boothroyd, H. Wijnen, F. Naef, L. Saez, M. W. Young, Integration of light and temperature in the regulation of circadian gene expression in *Drosophila*. PLOS Genet. 3, e54 (2007). 17411344 10.1371/journal.pgen.0030054 PMC1847695

[111] S. Montelli, G. Mazzotta, S. Vanin, L. Caccin, S. Corrà, C. De Pittà, C. Boothroyd, E. W. Green, C. P. Kyriacou, R. Costa, period and timeless mRNA splicing profiles under natural conditions in *Drosophila melanogaster*. J. Biol. Rhythms 30, 217–227 (2015). 25994101 10.1177/0748730415583575

[112] A. Martin Anduaga, N. Evantal, I. L. Patop, O. Bartok, R. Weiss, S. Kadener, Thermosensitive alternative splicing senses and mediates temperature adaptation in *Drosophila*. eLife 8, e44642 (2019). 31702556 10.7554/eLife.44642 PMC6890466

[113] E. A. Nettnin, T. R. Sallese, A. Nasseri, S. Saurabh, D. J. Cavanaugh, Dorsal clock neurons in *Drosophila* sculpt locomotor outputs but are dispensable for circadian activity rhythms. iScience 24, 103001 (2021). 34505011 10.1016/j.isci.2021.103001 PMC8413890

[114] Q. Liu, M. Tabuchi, S. Liu, L. Kodama, W. Horiuchi, J. Daniels, L. Chiu, D. Baldoni, M. N. Wu, Branch-specific plasticity of a bifunctional dopamine circuit encodes protein hunger. Science 356, 534–539 (2017). 28473588 10.1126/science.aal3245 PMC5513152

[115] M. P. Fernández, J. Berni, M. F. Ceriani, Circadian remodeling of neuronal circuits involved in rhythmic behavior. PLOS Biol. 6, e69 (2008). 18366255 10.1371/journal.pbio.0060069 PMC2270325

[116] J. M. Duhart, A. Herrero, G. de la Cruz, J. I. Ispizua, N. Pírez, M. F. Ceriani, Circadian structural plasticity drives remodeling of E cell output. Curr. Biol. 30, 5040–5048.e5 (2020). 33065014 10.1016/j.cub.2020.09.057

[117] E. A. Gorostiza, A. Depetris-Chauvin, L. Frenkel, N. Pirez, M. F. Ceriani, Circadian pacemaker neurons change synaptic contacts across the day. Curr. Biol. 24, 2161–2167 (2014). 25155512 10.1016/j.cub.2014.07.063 PMC4175170

[118] M. P. Fernandez, H. L. Pettibone, J. T. Bogart, C. J. Roell, C. E. Davey, A. Pranevicius, K. V. Huynh, S. M. Lennox, B. S. Kostadinov, O. T. Shafer, Sites of circadian clock neuron plasticity mediate sensory integration and entrainment. Curr. Biol. 30, 2225–2237.e5 (2020). 32386535 10.1016/j.cub.2020.04.025 PMC7314648

[119] A. Herrero, T. Yoshii, J. I. Ispizua, C. Colque, J. A. Veenstra, N. I. Muraro, M. F. Ceriani, Coupling neuropeptide levels to structural plasticity in *Drosophila* clock neurons. Curr. Biol. 30, 3154–3166.e4 (2020). 32619484 10.1016/j.cub.2020.06.009

[120] M. P. Nusbaum, D. M. Blitz, E. Marder, Functional consequences of neuropeptide and small-molecule co-transmission. Nat. Rev. Neurosci. 18, 389–403 (2017). 28592905 10.1038/nrn.2017.56 PMC5547741

[121] V. Svensson, R. Vento-Tormo, S. A. Teichmann, Exponential scaling of single-cell RNA-seq in the past decade. Nat. Protoc. 13, 599–604 (2018). 29494575 10.1038/nprot.2017.149

[122] L. Zhang, B. Y. Chung, B. C. Lear, V. L. Kilman, Y. Liu, G. Mahesh, R. A. Meissner, P. E. Hardin, R. Allada, DN1_p_ circadian neurons coordinate acute light and PDF inputs to produce robust daily behavior in *Drosophila*. Curr. Biol. 20, 591–599 (2010). 20362452 10.1016/j.cub.2010.02.056 PMC2864127

[123] S. Yadlapalli, C. Jiang, A. Bahle, P. Reddy, E. Meyhofer, O. T. Shafer, Circadian clock neurons constantly monitor environmental temperature to set sleep timing. Nature 555, 98–102 (2018). 29466329 10.1038/nature25740

[124] S. Terhzaz, P. Rosay, S. F. Goodwin, J. A. Veenstra, The neuropeptide SIFamide modulates sexual behavior in *Drosophila*. Biochem. Biophys. Res. Commun. 352, 305–310 (2007). 17126293 10.1016/j.bbrc.2006.11.030

[125] C. Martelli, U. Pech, S. Kobbenbring, D. Pauls, B. Bahl, M. V. Sommer, A. Pooryasin, J. Barth, C. W. P. Arias, C. Vassiliou, A. J. F. Luna, H. Poppinga, F. G. Richter, C. Wegener, A. Fiala, T. Riemensperger, SIFamide translates hunger signals into appetitive and feeding behavior in *Drosophila*. Cell Rep. 20, 464–478 (2017). 28700946 10.1016/j.celrep.2017.06.043

[126] A. P. Dreyer, M. M. Martin, C. V. Fulgham, D. A. Jabr, L. Bai, J. Beshel, D. J. Cavanaugh, A circadian output center controlling feeding:fasting rhythms in *Drosophila*. PLOS Genet. 15, e1008478 (2019). 31693685 10.1371/journal.pgen.1008478 PMC6860455

[127] B. J. Song, S. J. Sharp, D. Rogulja, Daily rewiring of a neural circuit generates a predictive model of environmental light. Sci. Adv. 7, eabe4284 (2021). 33762336 10.1126/sciadv.abe4284 PMC7990339

[128] V. Hartenstein, The neuroendocrine system of invertebrates: A developmental and evolutionary perspective. J. Endocrinol. 190, 555–570 (2006). 17003257 10.1677/joe.1.06964

[129] Y. H. Belgacem, J. R. Martin, Neuroendocrine control of a sexually dimorphic behavior by a few neurons of the pars intercerebralis in *Drosophila*. Proc. Natl. Acad. Sci. U.S.A. 99, 15154–15158 (2002). 12399547 10.1073/pnas.232244199 PMC137559

[130] K. Foltenyi, R. J. Greenspan, J. W. Newport, Activation of EGFR and ERK by rhomboid signaling regulates the consolidation and maintenance of sleep in *Drosophila*. Nat. Neurosci. 10, 1160–1167 (2007). 17694052 10.1038/nn1957

[131] J. B. Henningsen, F. Gauer, V. Simonneaux, RFRP neurons - The doorway to understanding seasonal reproduction in mammals. Front. Endocrinol. 7, 36 (2016). 10.3389/fendo.2016.00036 PMC4853402 27199893

[132] M. Brankatschk, T. Gutmann, O. Knittelfelder, A. Palladini, E. Prince, M. Grzybek, B. Brankatschk, A. Shevchenko, Ü. Coskun, S. Eaton, A temperature-dependent switch in feeding preference improves *Drosophila* development and survival in the cold. Dev. Cell 46, 781–793.e4 (2018). 30253170 10.1016/j.devcel.2018.05.028

[133] W. S. Lin, S. R. Yeh, S. Z. Fan, L. Y. Chen, J. H. Yen, T. F. Fu, M. S. Wu, P. Y. Wang, Insulin signaling in female *Drosophila* links diet and sexual attractiveness. FASEB J. 32, 3870–3877 (2018). 29475396 10.1096/fj.201800067R

[134] A. C. Keene, E. R. Duboue, D. M. McDonald, M. Dus, G. S. Suh, S. Waddell, J. Blau, Clock and cycle Limit Starvation-Induced Sleep Loss in Drosophila. Curr. Biol. 20, 1209–1215 (2010). 20541409 10.1016/j.cub.2010.05.029 PMC2929698

[135] M. Cavey, B. Collins, C. Bertet, J. Blau, Circadian rhythms in neuronal activity propagate through output circuits. Nat. Neurosci. 19, 587–595 (2016). 26928065 10.1038/nn.4263 PMC5066395

[136] K. J. Venken, K. L. Schulze, N. A. Haelterman, H. Pan, Y. He, M. Evans-Holm, J. W. Carlson, R. W. Levis, A. C. Spradling, R. A. Hoskins, H. J. Bellen, MiMIC: A highly versatile transposon insertion resource for engineering *Drosophila melanogaster* genes. Nat. Methods 8, 737–743 (2011). 21985007 10.1038/nmeth.1662 PMC3191940

[137] M. Z. Li, S. J. Elledge, Harnessing homologous recombination in vitro to generate recombinant DNA via SLIC. Nat. Methods 4, 251–256 (2007). 17293868 10.1038/nmeth1010

[138] S. Mansourian, A. Enjin, E. V. Jirle, V. Ramesh, G. Rehermann, P. G. Becher, J. E. Pool, M. C. Stensmyr, Wild African *Drosophila melanogaster* are seasonal specialists on Marula fruit. Curr. Biol. 28, 3960–3968.e3 (2018). 30528579 10.1016/j.cub.2018.10.033 PMC7065024

[139] L. Soto-Yéber, J. Soto-Ortiz, P. Godoy, R. Godoy-Herrera, The behavior of adult *Drosophila* in the wild. PLOS ONE 13, e0209917 (2018). 30596767 10.1371/journal.pone.0209917 PMC6312304

[140] M. S. Ross, L. B. Flanagan, G. H. L. Roi, Seasonal and successional changes in light quality and quantity in the understory of boreal forest ecosystems. Can. J. Bot. 24, 2792–2799 (1986).

[141] M. H. Turnbull, D. J. Yates, Seasonal variation in the red/far-red ratio and photon flux density in an Australian sub-tropical rainforest. Agric. For. Meteorol. 64, 111–127 (1993).

[142] H. R. Nuñez, R. C. de Gouvenain, Seasonal variation in understory light near a gap edge and its association with conifer seedling survival in a southern New England forest. Northeast Nat. 22, 613–629 (2015).

[143] S. M. Hartikainen, M. Pieristè, J. Lassila, T. M. Robson, Seasonal patterns in spectral irradiance and leaf UV-A absorbance under forest canopies. Front. Plant Sci. 10, 1762 (2019). 32133015 10.3389/fpls.2019.01762 PMC7040076

[144] A. Deckard, R. C. Anafi, J. B. Hogenesch, S. B. Haase, J. Harer, Design and analysis of large-scale biological rhythm studies: A comparison of algorithms for detecting periodic signals in biological data. Bioinformatics 29, 3174–3180 (2013). 24058056 10.1093/bioinformatics/btt541 PMC4471443

[145] O. M. Chaves, G. Avalos, Do seasonal changes in light availability influence the inverse leafing phenology of the neotropical dry forest understory shrub *Bonellia nervosa* (*Theophrastaceae*). Rev. Biol. Trop. 56, 257–268 (2008). 18624241

[146] S. Tripathi, R. Bhadouria, P. Srivastava, R. S. Devi, R. Chaturvedi, A. S. Raghubanshi, Effects of light availability on leaf attributes and seedling growth of four tree species in tropical dry forest. Ecol. Process 9, 2 (2020).

[147] C. Choi, G. Cao, A. K. Tanenhaus, E. V. McCarthy, M. Jung, W. Schleyer, Y. Shang, M. Rosbash, J. C. Yin, M. N. Nitabach, Autoreceptor control of peptide/neurotransmitter corelease from PDF neurons determines allocation of circadian activity in *Drosophila*. Cell Rep. 2, 332–344 (2012). 22938867 10.1016/j.celrep.2012.06.021 PMC3432947

[148] M. Damulewicz, G. M. Mazzotta, E. Sartori, E. Rosato, R. Costa, E. M. Pyza, Cryptochrome is a regulator of synaptic plasticity in the visual system of *Drosophila melanogaster*. Front. Mol. Neurosci. 10, 1660 (2017). 10.3389/fnmol.2017.00165 PMC5448152 28611590

[149] J. Bischof, E. M. Sheils, M. Bjorklund, K. Basler, Generation of a transgenic ORFeome library in *Drosophila*. Nat. Protoc. 9, 1607–1620 (2014). 24922270 10.1038/nprot.2014.105 PMC4150248

[150] B. Hudry, S. Viala, Y. Graba, S. Merabet, Visualization of protein interactions in living *Drosophila* embryos by the bimolecular fluorescence complementation assay. BMC Biol. 9, 5 (2011). 21276241 10.1186/1741-7007-9-5 PMC3041725

[151] F. Alejevski, A. Saint-Charles, C. Michard-Vanhée, B. Martin, S. Galant, D. Vasiliauskas, F. Rouyer, The HisCl1 histamine receptor acts in photoreceptors to synchronize *Drosophila* behavioral rhythms with light-dark cycles. Nat. Commun. 10, 252 (2019). 30651542 10.1038/s41467-018-08116-7 PMC6335465

[152] A. Klarsfeld, S. Malpel, C. Michard-Vanhée, M. Picot, E. Chélot, F. Rouyer, Novel features of cryptochrome-mediated photoreception in the brain circadian clock of *Drosophila*. J. Neurosci. 24, 1468–1477 (2004). 14960620 10.1523/JNEUROSCI.3661-03.2004 PMC6730330

[153] J. O. Gummadova, G. A. Coutts, N. R. Glossop, Analysis of the *Drosophila* clock promoter reveals heterogeneity in expression between subgroups of central oscillator cells and identifies a novel enhancer region. J. Biol. Rhythms 24, 353–367 (2009). 19755581 10.1177/0748730409343890

[154] M. T. Li, L. H. Cao, N. Xiao, M. Tang, B. Deng, T. Yang, T. Yoshii, D. G. Luo, Hub-organized parallel circuits of central circadian pacemaker neurons for visual photoentrainment in *Drosophila*. Nat. Commun. 9, 4247 (2018). 30315165 10.1038/s41467-018-06506-5 PMC6185921

[155] J. H. Bahn, G. Lee, J. H. Park, Comparative analysis of Pdf-mediated circadian behaviors between Drosophila melanogaster and D. virilis. Genetics 181, 965–975 (2009). 19153257 10.1534/genetics.108.099069 PMC2651067

[156] W. Li, J. T. Ohlmeyer, M. E. Lane, D. Kalderon, Function of protein kinase A in hedgehog signal transduction and *Drosophila* imaginal disc development. Cell 80, 553–562 (1995). 7867063 10.1016/0092-8674(95)90509-x

[157] E. Blanchardon, B. Grima, A. Klarsfeld, E. Chélot, P. E. Hardin, T. Préat, F. Rouyer, Defining the role of *Drosophila* lateral neurons in the control of circadian rhythms in motor activity and eclosion by targeted genetic ablation and PERIOD protein overexpression. Eur. J. Neurosci. 13, 871–888 (2001). 11264660 10.1046/j.0953-816x.2000.01450.x

[158] T. Copf, Developmental shaping of dendritic arbors in *Drosophila* relies on tightly regulated intra-neuronal activity of protein kinase A (PKA). Dev. Biol. 393, 282–297 (2014). 25017992 10.1016/j.ydbio.2014.07.002

[159] H. Kanuka, E. Kuranaga, K. Takemoto, T. Hiratou, H. Okano, M. Miura, Drosophila caspase transduces Shaggy/GSK-3beta kinase activity in neural precursor development. EMBO J. 24, 3793–3806 (2005). 16222340 10.1038/sj.emboj.7600822 PMC1276714

[160] S. Tanoue, P. Krishnan, B. Krishnan, S. E. Dryer, P. E. Hardin, Circadian clocks in antennal neurons are necessary and sufficient for olfaction rhythms in *Drosophila*. Curr. Biol. 14, 638–649 (2004). 15084278 10.1016/j.cub.2004.04.009

[161] M. J. Muskus, F. Preuss, J. Y. Fan, E. S. Bjes, J. L. Price, Drosophila DBT lacking protein kinase activity produces long-period and arrhythmic circadian behavioral and molecular rhythms. Mol. Cell. Biol. 27, 8049–8064 (2007). 17893330 10.1128/MCB.00680-07 PMC2169192

[162] S. Hyun, Y. Lee, S. T. Hong, S. Bang, D. Paik, J. Kang, J. Shin, J. Lee, K. Jeon, S. Hwang, E. Bae, J. Kim, Drosophila GPCR Han is a receptor for the circadian clock neuropeptide PDF. Neuron 48, 267–278 (2005). 16242407 10.1016/j.neuron.2005.08.025

[163] M. D. Gordon, K. Scott, Motor control in a *Drosophila* taste circuit. Neuron 61, 373–384 (2009). 19217375 10.1016/j.neuron.2008.12.033 PMC2650400

[164] K. Feng, M. T. Palfreyman, M. Hasemeyer, A. Talsma, B. J. Dickson, Ascending SAG neurons control sexual receptivity of *Drosophila* females. Neuron 83, 135–148 (2014). 24991958 10.1016/j.neuron.2014.05.017

