# Supplementary material for: Neuropeptide dynamics coordinate layered plasticity mechanisms adapting Drosophila circadian behavior to changing environment
Source: Sci Adv. 2025 Aug 29;11(35):eadt7168. doi: 10.1126/sciadv.adt7168 (PMC12396332; doi:10.1126/sciadv.adt7168)
Supplement: Supplementary file 1 — Figs. S1 to S5 Table S1 References [file sciadv.adt7168_sm.pdf]

Science Advances
AAAS

Supplementary Materials for

Neuropeptide dynamics coordinate layered plasticity mechanisms adapting *Drosophila* circadian behavior to changing environment

Abhishek Chatterjee *et al.*

Corresponding author: François Rouyer, francois.rouyer@universite-paris-saclay.fr;
Abhishek Chatterjee, abhishek.chatterjee@inrae.fr

Sci. Adv. **11**, eadt7168 (2025)
DOI: 10.1126/sciadv.adt7168

This PDF file includes:

Figs. S1 to S5
Table S1
References

A

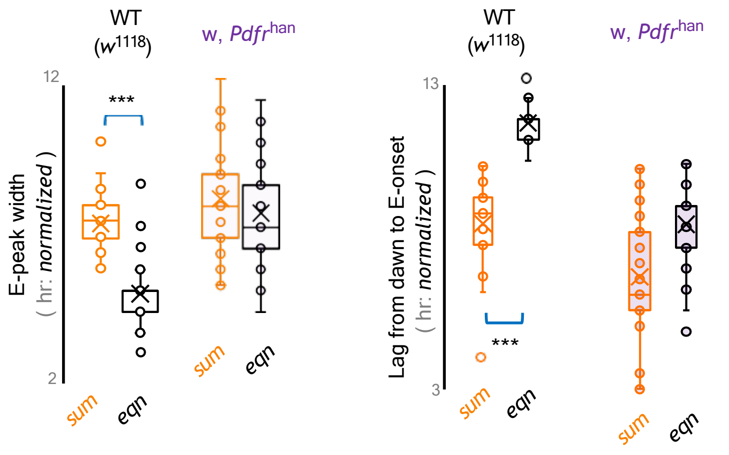

	sum	Eqn	Sum	Eqn
E-peak width	8.1 ±0.3	5.4 ±0.5	7.6 ±0.2	5.5 ±0.3
lag of E-onset (*norm*)	6.4 ±0.2	8.7 ±0.4	6.7 ±0.2	9.0 ±0.3
	Viltain		Berlin	

B

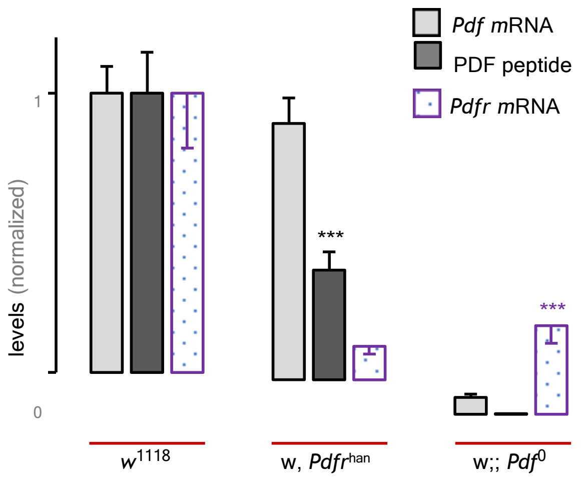

C

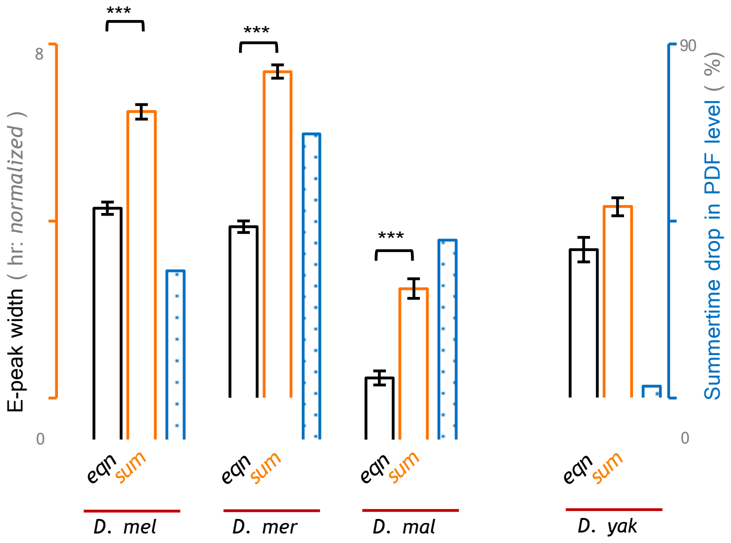

D

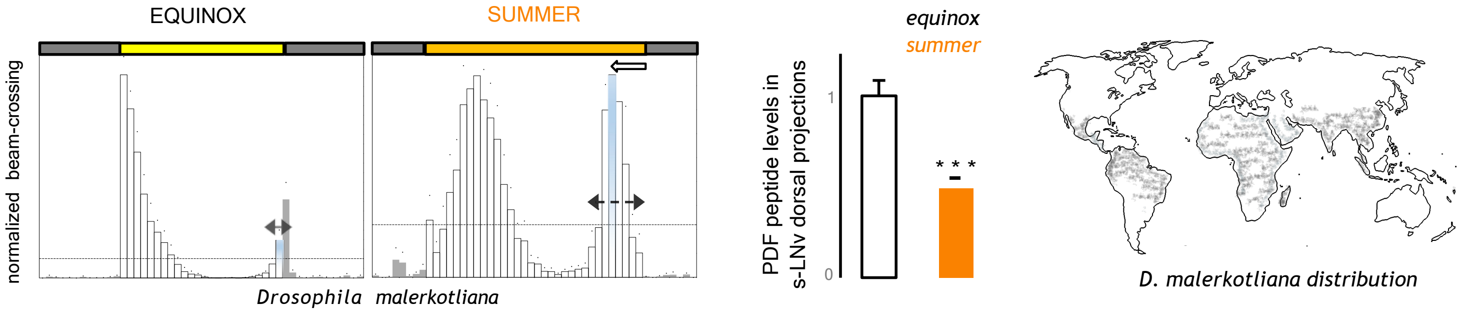

Figure S1. The width and phase of E activity are correlated with seasonally varying PDF

A. In summer, the E-activity is both expanded and advanced in wild-type (*w*^{1118}) flies. The width of E-peak is broader and the onset of the E-activity is advanced in summer compared to equinox. Both these differences largely disappear in *Pdfr*^{han} mutants. (Table) Recently wild-caught strains like *Viltain* and standard laboratory strains like *Berlin* also exhibit broader, advanced E-activity on summer days. Normalized E-peak width and the lag of E-onset from the dawn light-ON transition are in hours.

B. The absence of PDFR decreases PDF peptide levels. On the other hand, absence of PDF decreases *Pdfr* mRNA. This is in accordance with our finding that, in summer, not only PDF levels in the LNvs but also its receptor levels drop in the LNd and DN1p.

C. In summer, broadening of the E-activity is associated with a decrease in PDF levels in different Drosophilids. Cosmopolitan *melanogaster* and *mercatorum* exhibit both broadening of E-activity and drop in PDF levels in summer whereas afrotropical *yakuba* exhibit neither.

D. Moderately distributed *malerkotliana,* like *melanogaster* and *mercatorum* and unlike *yakuba*, exhibit both broadening of E-activity and an associated drop in PDF levels.

A

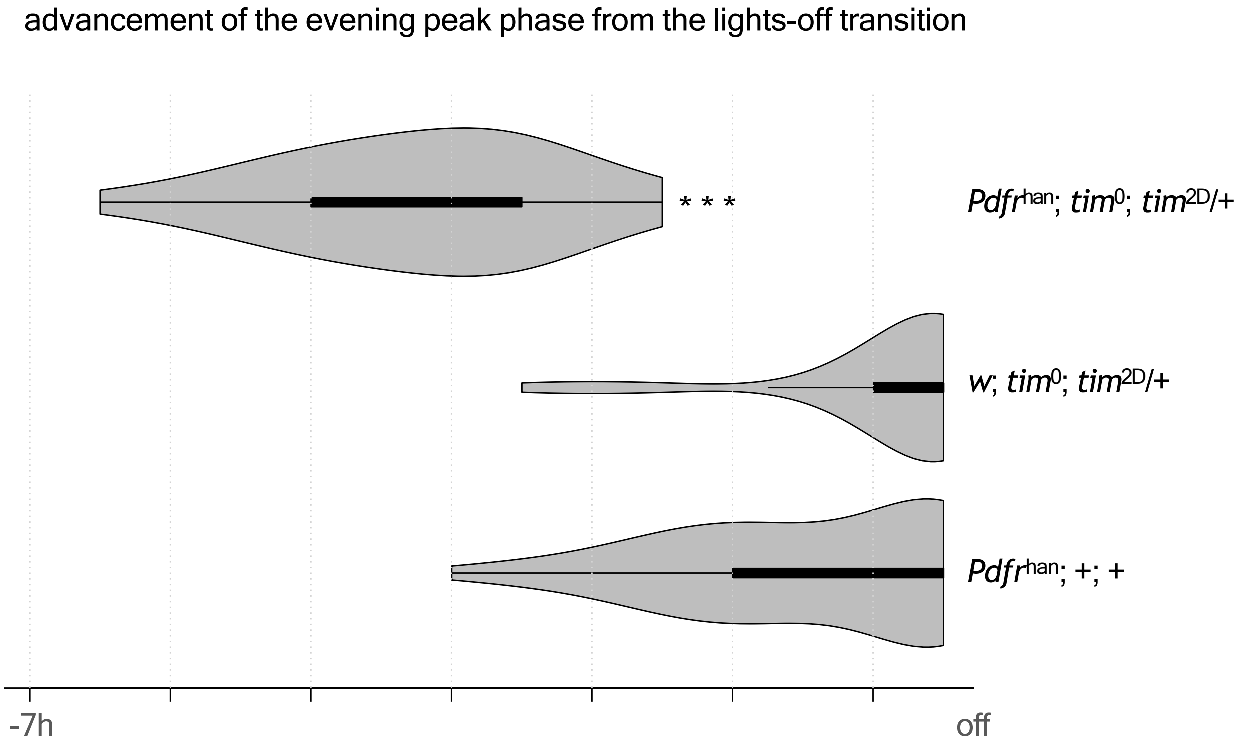
advancement of the evening peak phase from the lights-off transition
* * *
Pdfrhan; tim0; tim2D/+
w; tim0; tim2D/+
Pdfrhan; +; +
-7h
off

B

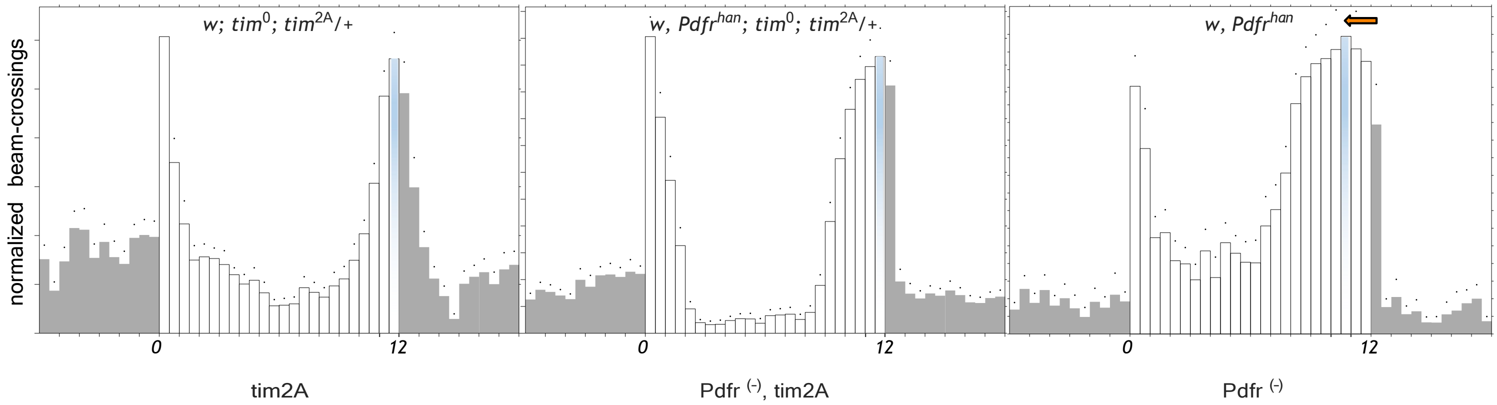
normalized beam-crossings
w; tim0; tim2A/+
w, Pdfrhan; tim0; tim2A/+
w, Pdfrhan
0
12
tim2A
Pdfr (-), tim2A
Pdfr (-)

Figure S2. PDF signaling impinges onto TIM to regulate E phase

A. Quantification of a genetic interaction between a variant of TIM (TIM^{2D}) and *Pdfr*^{han}. Advancement of the E-peak phase from the lights OFF transition is greater in the flies with both $Pdfr^{han}$ mutation and TIM^{2D} variant of TIM than flies with only *Pdfr*^{han} mutation or TIM^{2D} variant of TIM.

B. *Pdfr*^{han} mutants with wild-type *tim* have a 1.5-2 hr advanced E-peak (arrow) under equinox conditions. However, when TIM (*tim*^{2A}) is phospho-deficient for SGG, the same *Pdfr*^{han} mutation is unable to bring out any striking advancement of the E-peak.

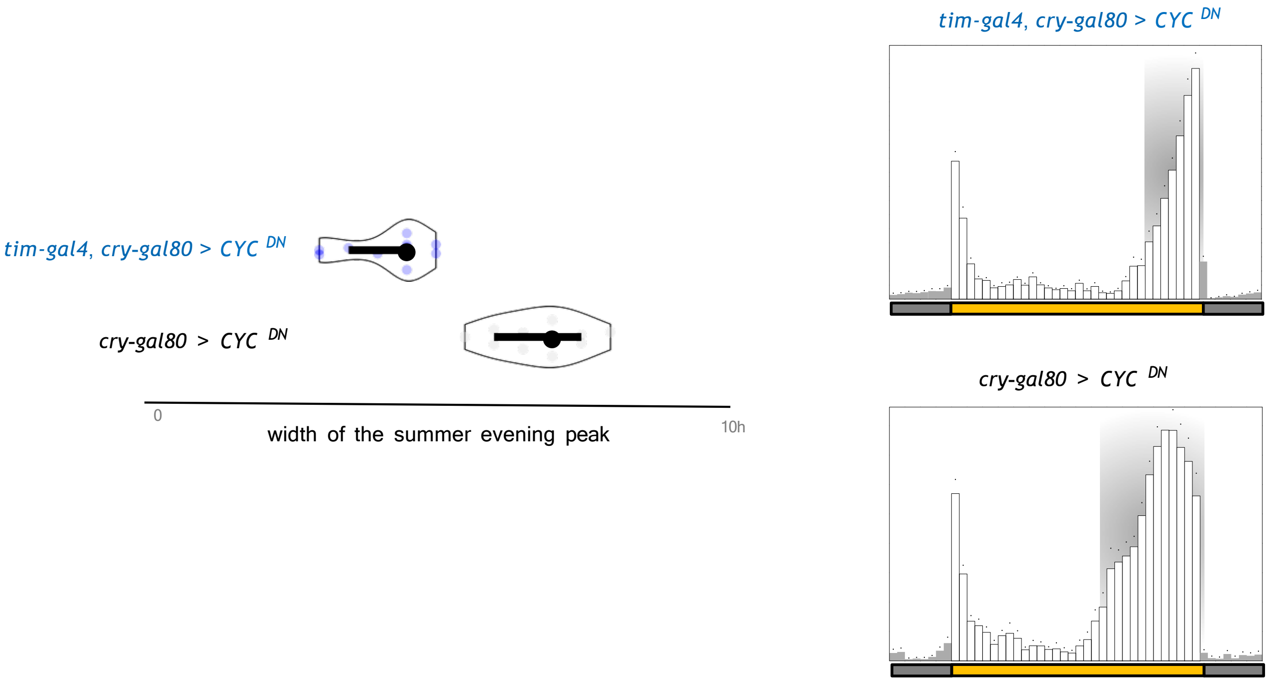
tim-gal4, cry-gal80 > CYC DN
cry-gal80 > CYC DN
0
width of the summer evening peak
10h
tim-gal4, cry-gal80 > CYC DN
cry-gal80 > CYC DN

Figure S3. An oscillator for summer

A functional clock in the tim-gal4, cry-gal80 neurons is necessary for the expanded E-activity in summer. Expression of dominant negative variant of CYCLE in tim-gal4, cry-gal80 DN1p neurons diminished the width of summer E peak.

A

B

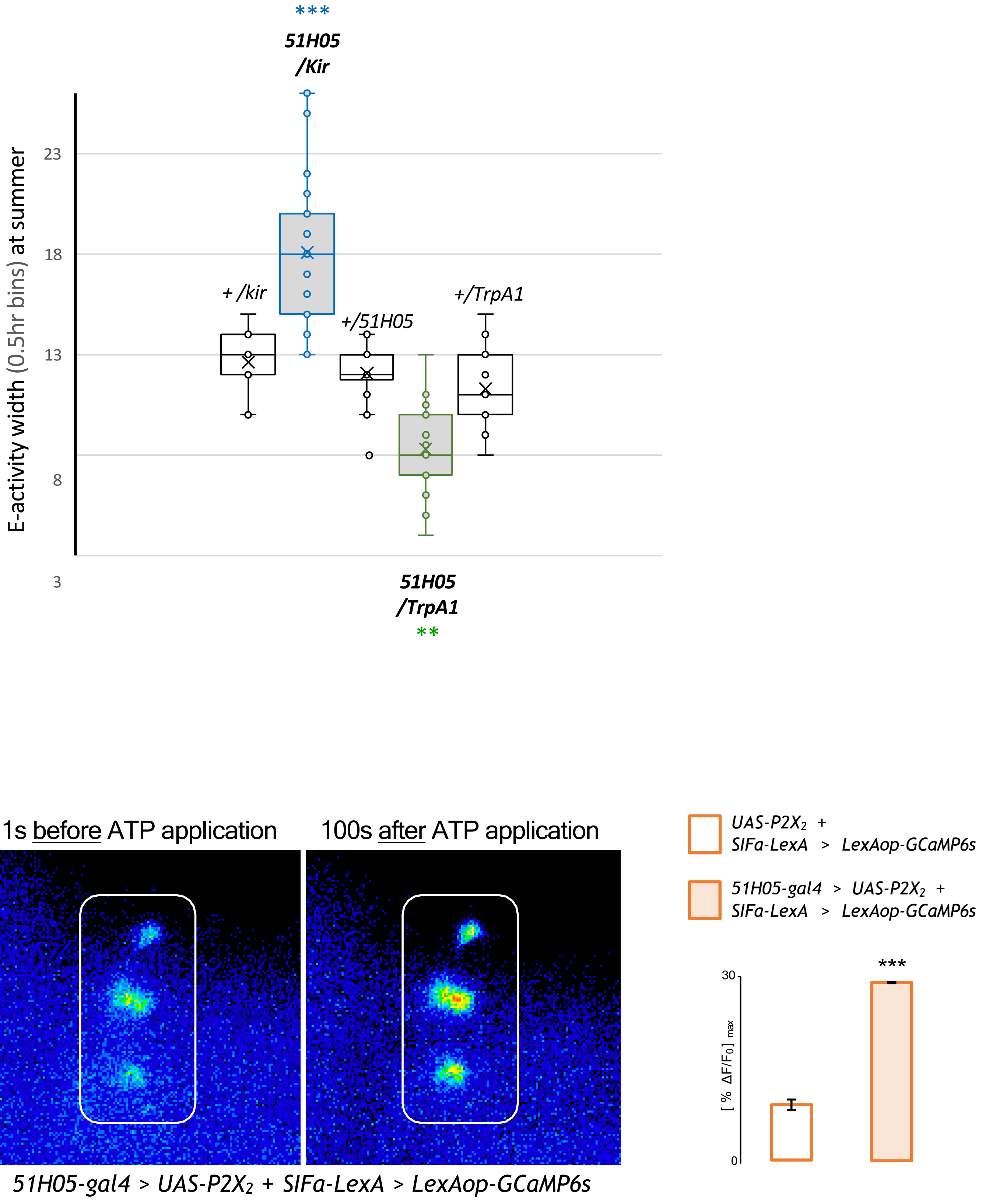

***
51H05
/Kir
E-activity width (0.5hr bins) at summer
23
18
13
8
3
+ /kir
+/51H05
+/TrpA1
51H05
/TrpA1
**
1s before ATP application
100s after ATP application
51H05-gal4 > UAS-P2X2 + SIFa-LexA > LexAop-GCaMP6s
UAS-P2X2 +
SIFa-LexA > LexAop-GCaMP6s
51H05-gal4 > UAS-P2X2 +
SIFa-LexA > LexAop-GCaMP6s
[% ΔF/F0] max
30
0
***

Figure S4. Locomotor-suppressive 51H05 neurons functionally communicate to the SIF*a* neurons

A. 51H05 neurons suppress locomotor activity. Targeted Kir2.1 expression in these neurons increased the width of E-activity in summer. On the other hand, TrpA1 expression in these neurons and elevating the temperature from 22ºC to 30ºC for 24 hours reduced the width of E-activity in summer.

B. Representative result (left panel) and quantification (right panel) from live-imaging experiments in explanted Drosophila brain highlighting that pharmacogenetic activation of the 51H05 DN1p neurons via the ATP-P2X2 system (0.5 mM ATP) triggers calcium rise in the SIF*a* neurons, which is reflected by an increase in GCaMP fluorescence.

A

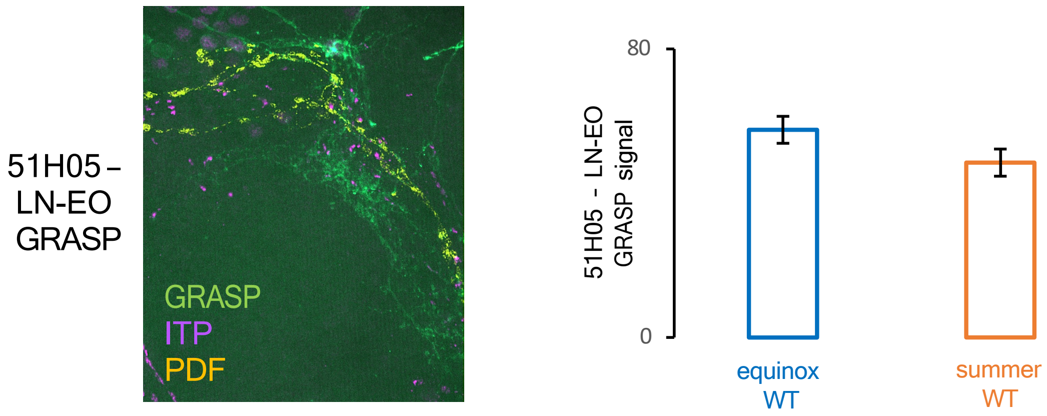

51H05 –
LN-EO
GRASP
GRASP
ITP
PDF
80
0
51H05 - LN-EO
GRASP signal
equinox
WT
summer
WT

B

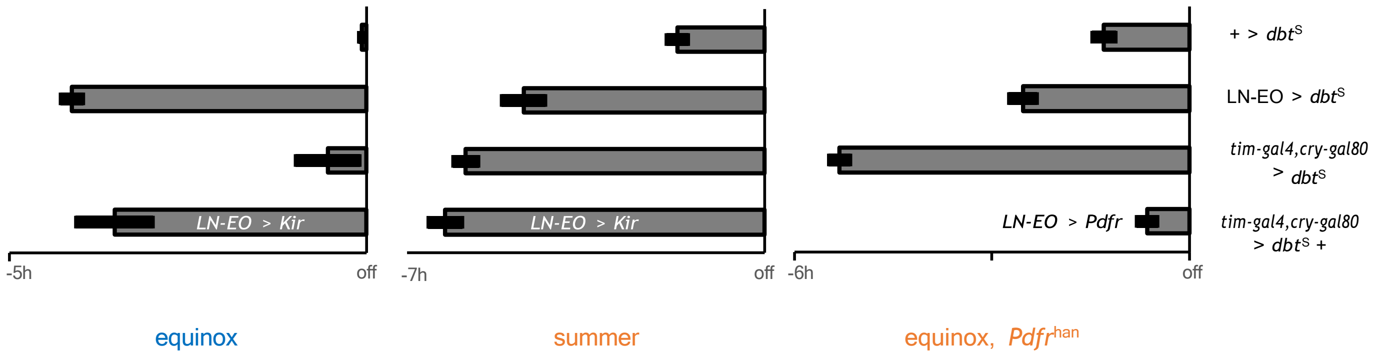

+ > dbt^S
LN-EO > dbt^S
tim-gal4,cry-gal80
> dbt^S
tim-gal4,cry-gal80
> dbt^S +
LN-EO > Kir
LN-EO > Kir
LN-EO > Pdfr
-5h
off
-7h
off
-6h
off
equinox
summer
equinox, Pdfr^han

Figure S5. Seasonally conditioned partitioning of function between LN-EO and 51H05 DN1ps

A. 51H05 DN1ps project ventrally toward the LNds. The strength of this anatomical connection does not vary according to the season. The GRASP signal between 51H05 DN1ps and LN-EO (ITP+ LNd and the 5th sLNv) does not differ between equinox and summer.

B. Seasonal structuring of the E-activity: The phase of the E-activity is regulated by the LN-EO in the ITP+ clock neurons. The 51H05 DN1ps achieve a conditional gain in their importance in phasing E activity in summer-like conditions or in absence of *Pdfr* in equinox or when the LN-EO was functionally removed from the circuit by expressing *Kir*. Additionally, *Pdfr* signaling in the LN-EO is crucial for designating context-dependent functional gain to the 51H05 DN1ps.

**Wild type**	
Drosophila mercatorum	NDSSC (Cornell)
Drosophila yakuba	Jean David, EGCE, Gif-sur-Yvette
Drosophila malerkotliana	Jean David, EGCE, Gif-sur-Yvette
Drosophila melanogaster	w1118 isogenized in Canton-S background for 10 generations
Drosophila melanogaster (Viltain)	Amir Yassin, EGCE, Gif-sur-Yvette
Drosophila melanogaster (Berlin)	Jean-Rene Martin, NeuroPSI, Saclay
**Mutants**	
pdfrhan5304,w	BDSC#33068
pdf0	BDSC#26654
tim0	BDSC#80930
tim2D	*(65)*
per0	BDSC#80928
cry0	BDSC#86227
**Gal4/LexA/Gal80**	
Insite 1190-gal4	BDSC#65501
cry-gal4(39)	(*152*)
cry-gal4(19)	*(29)*
Clk 6939 gal4	(153)
Clk4.1M-gal4	BDSC#36316
tim-gal4	BDSC#7126
cry-gal80	*(17)*
51H05-gal4	BDSC#41275
Clk4.1M-LexA	BDSC#80704
Clk4.1M-gal80	this paper
18H11-gal4	BDSC#48832
MB122B-gal4	*(75)*
51H05-AD	BDSC#70720
ChAT-DBD	BDSC#60318
SIFa-LexA	*(125)*
SIFa-gal4	*(124)*
dvPdf(LN)-LexA	(154)
dvPdf(LN)-gal4	(155)
92H07-LexA	BDSC#54396
ChAT-gal80	BDSC#60321
mGluR-gal4	BDSC#77721
cry-LexA::GAD	this paper
12G04LexA	BDSC#52448
**UAS / LexAop**	
UAS-cd8:GFP	BDSC#32185
UAS-GFP	BDSC#1521
UAS-nls:GFP	BDSC#4775
UAS-PKA-mC* (CA)	(156)
UAS-SGG - KK83-84MI (kinase dead)	BDSC#8712

UAS-SGG-S9A (CA)	BDSC#5255
UAS-sggB	BDSC#5361
UAS-lacZ	BDSC#3955
UAS-Pka-R1.BDK	BDSC#35550
UAS-PER	(157)
UAS-DenMark , UAS syt:GFP	BDSC#33065
UAS-PKA-GFP	(158)
UAS-sgg.10.MYC	(*159*)
UAS-tim-CRISPR KO	BDSC#83100
UAS-Cas9.P2	BDSC#58985
UAS-cycDN	BDSC#36316
UAS-TrpA1	BDSC#26264
UAS-CsChrimson	BDSC#55135
UAS-ChR2-XXL / CsChrimson	BDSC#58374
UAS-GCaMP6s	BDSC#42746
UAS-RFP	BDSC#30556
UAS-hid,rpr	(160)
UAS-Kir	BDSC#6596
UAS-CaLexA; LexAop-GFP	BDSC#66542
UAS-dbts	(161)
UAS-Pdfr	(*162*)
UAS-SGG RNAi HMS01751	BDSC#38293
MCFO	BDSC#64086
GRASP	(*163*)
transTANGO'	BDSC#77124
LexAop-myrRFP	BDSC#56142
LexAop-P2X2	BDSC#76030
LexAop-rpr	(154)
LexAop-Kir	(164)

Table S1. List of the fly lines used in the study

Left: genotype, right: source.

REFERENCES AND NOTES

1. A. Patke, M. W. Young, S. Axelrod, Molecular mechanisms and physiological importance of circadian rhythms. *Nat. Rev. Mol. Cell Biol.* **21**, 67–84 (2020).

2. S. Michel, J. H. Meijer, From clock to functional pacemaker. *Eur. J. Neurosci.* **51**, 482–493 (2020).

3. M. Ahmad, W. Li, D. Top, Integration of circadian clock information in the *Drosophila* circadian neuronal network. *J. Biol. Rhythms* **36**, 203–220 (2021).

4. S. L. Crespo-Flores, A. F. Barber, The *Drosophila* circadian clock circuit is a nonhierarchical network of peptidergic oscillators. *Curr. Opin. Insect Sci.* **52**, 100944 (2022).

5. D. Sidote, J. Majercak, V. Parikh, I. Edery, Differential effects of light and heat on the *Drosophila* circadian clock proteins PER and TIM. *Mol. Cell. Biol.* **18**, 2004–2013 (1998).

6. J. Majercak, D. Sidote, P. E. Hardin, I. Edery, How a circadian clock adapts to seasonal decreases in temperature and day length. *Neuron* **24**, 219–230 (1999).

7. C. Helfrich-Förster, E. Bertolini, P. Menegazzi, Flies as models for circadian clock adaptation to environmental challenges. *Eur. J. Neurosci.* **51**, 166–181 (2020).

8. C. Helfrich-Förster, Light input pathways to the circadian clock of insects with an emphasis on the fruit fly *Drosophila* melanogaster. *J. Comp. Physiol. A Neuroethol. Sens. Neural Behav. Physiol.* **206**, 259–272 (2020).

9. R. George, R. Stanewsky, Peripheral sensory organs contribute to temperature synchronization of the circadian clock in *Drosophila* melanogaster. *Front. Physiol.* **12**, 622545 (2021).

10. Y. Zhang, Y. Liu, D. Bilodeau-Wentworth, P. E. Hardin, P. Emery, Light and temperature control the contribution of specific DN1 neurons to *Drosophila* circadian behavior. *Curr. Biol.* **20**, 600–605 (2010).

11. A. Chatterjee, A. Lamaze, J. De, W. Mena, E. Chélot, B. Martin, P. Hardin, S. Kadener, P. Emery, F. Rouyer, Reconfiguration of a multi-oscillator network by light in the *Drosophila* circadian clock. *Curr. Biol.* **28**, 2007–2017 (2018).

12. M. Schlichting, P. Weidner, M. Diaz, P. Menegazzi, E. Dalla Benetta, C. Helfrich-Förster, M. Rosbash, Light-mediated circuit switching in the *Drosophila* neuronal clock network. *Curr. Biol.* **29**, 3266–3276.e3 (2019).

13. O. T. Shafer, A. C. Keene, The regulation of *Drosophila* sleep. *Curr. Biol.* **31**, R38–R49 (2021).

14. D. Monsivais, A. Ghosh, K. Bhattacharya, R. I. M. Dunbar, K. Kaski, Tracking urban human activity from mobile phone calling patterns. *PLOS Comput. Biol.* **13**, e1005824 (2017).

15. T. Roenneberg, Twitter as a means to study temporal behaviour. *Curr. Biol.* **27**, R830–R832 (2017).

16. B. Grima, E. Chélot, R. Xia, F. Rouyer, Morning and evening peaks of activity rely on different clock neurons of the *Drosophila* brain. *Nature* **431**, 869–873 (2004).

17. D. Stoleru, P. Peng, J. Agosto, M. Rosbash, Coupled oscillators control morning and evening locomotor behavior of *Drosophila*. *Nature* **431**, 862–868 (2004).

18. D. Rieger, O. T. Shafer, K. Tomioka, C. Helfrich-Forster, Functional analysis of circadian pacemaker neurons in *Drosophila* melanogaster. *J. Neurosci.* **26**, 2531–2543 (2006).

19. X. Liang, T. E. Holy, P. H. Taghert, Synchronous *Drosophila* circadian pacemakers display nonsynchronous Ca^{2+} rhythms in vivo. *Science* **351**, 976–981 (2016).

20. E. Bünning, Circadian rhythms and the time measurement in photoperiodism. *Cold Spring Harb. Symp. Quant. Biol.* **25**, 249–256 (1960).

21. R. A. Hut, S. Paolucci, R. Dor, C. P. Kyriacou, S. Daan, Latitudinal clines: An evolutionary view on biological rhythms. *Proc. Biol. Sci.* **280**, 20130433 (2013).

22. D. Saunders, Insect photoperiodism: Bunning's hypothesis, the history and development of an idea. *Eur. J. Entomol.* **118**, 1–13 (2021).

23. H. T. Vanderleest, T. Houben, S. Michel, T. Deboer, H. Albus, M. J. Vansteensel, G. D. Block, J. H. Meijer, Seasonal encoding by the circadian pacemaker of the SCN. *Curr. Biol.* **17**, 468–473 (2007).

24. E. A. Lucassen, H. C. van Diepen, T. Houben, S. Michel, C. S. Colwell, J. H. Meijer, Role of vasoactive intestinal peptide in seasonal encoding by the suprachiasmatic nucleus clock. *Eur. J. Neurosci.* **35**, 1466–1474 (2012).

25. A. Porcu, A. Nilsson, S. Booreddy, S. A. Barnes, D. K. Welsh, D. Dulcis, Seasonal changes in day length induce multisynaptic neurotransmitter switching to regulate hypothalamic network activity and behavior. *Sci. Adv.* **8**, eabn9867 (2022).

26. S. Michel, L. Kervezee, One seasonal clock fits all? *J. Comp. Physiol. A Neuroethol. Sens. Neural Behav. Physiol.* **210**, 641–647 (2024).

27. P. Menegazzi, S. Vanin, T. Yoshii, D. Rieger, C. Hermann, V. Dusik, C. P. Kyriacou, C. Helfrich- Forster, R. Costa, Drosophila clock neurons under natural conditions. *J. Biol. Rhythms* **28**, 3–14 (2013).

28. K. Tomioka, M. Yukizane, A specific area of the compound eye in the cricket *Gryllus* bimaculatus sends photic information to the circadian pacemaker in the contralateral optic lobe. *J. Comp. Physiol. A* **180**, 63–70 (1997).

29. M. Picot, P. Cusumano, A. Klarsfeld, R. Ueda, F. Rouyer, Light activates output from evening neurons and inhibits output from morning neurons in the *Drosophila* circadian clock. *PLOS Biol.* **5**, e315 (2007).

30. D. Stoleru, P. Nawathean, L. Fernandez Mde, J. S. Menet, M. F. Ceriani, M. Rosbash, The *Drosophila* circadian network is a seasonal timer. *Cell* **129**, 207–219 (2007).

31. O. T. Shafer, J. D. Levine, J. W. Truman, J. C. Hall, Flies by night: Effects of changing day length on *Drosophila*'s circadian clock. *Curr. Biol.* **14**, 424–432 (2004).

32. W. Bywalez, P. Menegazzi, D. Rieger, B. Schmid, C. Helfrich-Forster, T. Yoshii, The dual-oscillator system of *Drosophila melanogaster* under natural-like temperature cycles. *Chronobiol. Int.* **29**, 395–407 (2012).

33. C. Pittendrigh, S. Daan, A functional analysis of circadian pacemakers in nocturnal rodents. IV. Entrainment: Pacemaker as clock. *J. Comp. Physiol.* **106**, 291–331 (1976).

34. S. Vanin, S. Bhutani, S. Montelli, P. Menegazzi, E. W. Green, M. Pegoraro, F. Sandrelli, R. Costa, C. P. Kyriacou, Unexpected features of *Drosophila* circadian behavioural rhythms under natural conditions. *Nature* **484**, 371–375 (2012).

35. P. Menegazzi, T. Yoshii, C. Helfrich-Forster, Laboratory versus nature: The two sides of the *Drosophila* circadian clock. *J. Biol. Rhythms* **27**, 433–442 (2012).

36. J. De, V. Varma, S. Saha, V. Sheeba, V. K. Sharma, Significance of activity peaks in fruit flies, *Drosophila melanogaster*, under seminatural conditions. *Proc. Natl. Acad. Sci. U.S.A.* **110**, 8984–8989 (2013).

37. E. W. Green, E. K. O'Callaghan, C. N. Hansen, S. Bastianello, S. Bhutani, S. Vanin, J. D. Armstrong, R. Costa, C. P. Kyriacou, *Drosophila* circadian rhythms in seminatural environments: Summer afternoon component is not an artifact and requires TrpA1 channels. *Proc. Natl. Acad. Sci. U.S.A.* **112**, 8702–8707 (2015).

38. H. Kauranen, P. Menegazzi, R. Costa, C. Helfrich-Forster, A. Kankainen, A. Hoikkala, Flies in the north: Locomotor behavior and clock neuron organization of *Drosophila montana*. *J. Biol. Rhythms* **27**, 377–387 (2012).

39. P. Menegazzi, E. Dalla Benetta, M. Beauchamp, M. Schlichting, I. Steffan-Dewenter, C. Helfrich-Förster, Adaptation of circadian neuronal network to photoperiod in high-Latitude european *Drosophilids*. *Curr. Biol.* **27**, 833–839 (2017).

40. E. Bertolini, F. K. Schubert, D. Zanini, H. Sehadová, C. Helfrich-Förster, P. Menegazzi, Life at high latitudes does not require circadian behavioral rhythmicity under constant darkness. *Curr. Biol.* **29**, 3928–3936.e3 (2019).

41. D. Nagy, P. Cusumano, G. Andreatta, A. M. Anduaga, C. Hermann-Luibl, N. Reinhard, J. Gesto, C. Wegener, G. Mazzotta, E. Rosato, C. P. Kyriacou, C. Helfrich-Förster, R. Costa, Peptidergic signaling from clock neurons regulates reproductive dormancy in *Drosophila melanogaster*. *PLOS Genet.* **15**, e1008158 (2019).

42. S. Hidalgo, M. Anguiano, C. A. Tabuloc, J. C. Chiu, Seasonal cues act through the circadian clock and pigment-dispersing factor to control EYES ABSENT and downstream physiological changes. *Curr. Biol.* **33**, 675–687.e5 (2023).

43. S. Hidalgo, J. C. Chiu, Integration of photoperiodic and temperature cues by the circadian clock to regulate insect seasonal adaptations. *J. Comp. Physiol. A Neuroethol. Sens. Neural Behav. Physiol.* **210**, 585–599 (2024).

44. C. Helfrich-Förster, Neuropeptidergic regulation of insect diapause by the circadian clock. *Curr. Opin. Insect Sci.* **63**, 101198 (2024).

45. A. Abrieux, Y. Xue, Y. Cai, K. M. Lewald, H. N. Nguyen, Y. Zhang, J. C. Chiu, EYES ABSENT and TIMELESS integrate photoperiodic and temperature cues to regulate seasonal physiology in *Drosophila*. *Proc. Natl. Acad. Sci. U.S.A.* **117**, 15293–15304 (2020).

46. S. C. Renn, J. H. Park, M. Rosbash, J. C. Hall, P. H. Taghert, A pdf neuropeptide gene mutation and ablation of PDF neurons each cause severe abnormalities of behavioral circadian rhythms in *Drosophila*. *Cell* **99**, 791–802 (1999).

47. T. Yoshii, C. Wulbeck, H. Sehadova, S. Veleri, D. Bichler, R. Stanewsky, C. Helfrich-Forster, The neuropeptide pigment-dispersing factor adjusts period and phase of *Drosophila*'s clock. *J. Neurosci.* **29**, 2597–2610 (2009).

48. P. Cusumano, A. Klarsfeld, E. Chélot, M. Picot, B. Richier, F. Rouyer, PDF-modulated visual inputs and cryptochrome define diurnal behavior in *Drosophila*. *Nat. Neurosci.* **12**, 1431–1437 (2009).

49. B. C. Lear, L. Zhang, R. Allada, The neuropeptide PDF acts directly on evening pacemaker neurons to regulate multiple features of circadian behavior. *PLOS Biol.* **7**, e1000154 (2009).

50. L. Zhang, B. C. Lear, A. Seluzicki, R. Allada, The CRYPTOCHROME photoreceptor gates PDF neuropeptide signaling to set circadian network hierarchy in *Drosophila*. *Curr. Biol.* **19**, 2050–2055 (2009).

51. M. Schlichting, P. Menegazzi, K. R. Lelito, Z. Yao, E. Buhl, E. Dalla Benetta, A. Bahle, J. Denike, J. J. Hodge, C. Helfrich-Förster, O. T. Shafer, A neural network underlying circadian entrainment and photoperiodic adjustment of cleep and activity in *Drosophila*. *J. Neurosci.* **36**, 9084–9096 (2016).

52. K. M. Vaze, C. Helfrich-Förster, The neuropeptide PDF is crucial for delaying the phase of *Drosophila*'s evening neurons under long zeitgeber periods. *J. Biol. Rhythms* **36**, 442–460 (2021).

53. W. Bachleitner, L. Kempinger, C. Wulbeck, D. Rieger, C. Helfrich-Forster, Moonlight shifts the endogenous clock of *Drosophila* melanogaster. *Proc. Natl. Acad. Sci. U.S.A.* **104**, 3538–3543 (2007).

54. D. Rieger, C. Fraunholz, J. Popp, D. Bichler, R. Dittmann, C. Helfrich-Forster, The fruit fly *Drosophila melanogaster* favors dim light and times its activity peaks to early dawn and late dusk. *J. Biol. Rhythms* **22**, 387–399 (2007).

55. S. Lazopulo, A. Lazopulo, J. D. Baker, S. Syed, Daytime colour preference in *Drosophila* depends on the circadian clock and TRP channels. *Nature* **574**, 108–111 (2019).

56. G. T. Meyerhof, S. Easwaran, A. E. Bontempo, C. Montell, D. J. Montell, Altered circadian rhythm, sleep, and rhodopsin 7-dependent shade preference during diapause in *Drosophila melanogaster*. *Proc. Natl. Acad. Sci. U.S.A.* **121**, e2400964121 (2024).

57. H. Kauranen, O. Ala-Honkola, M. Kankare, A. Hoikkala, Circadian clock of *Drosophila montana* is adapted to high variation in summer day lengths and temperatures prevailing at high latitudes. *J. Insect Physiol.* **89**, 9–18 (2016).

58. M. Beauchamp, E. Bertolini, P. Deppisch, J. Steubing, P. Menegazzi, C. Helfrich-Förster, Closely related fruit fly species living at different latitudes diverge in their circadian clock anatomy and rhythmic behavior. *J. Biol. Rhythms* **33**, 602–613 (2018).

59. K. H. Low, C. Lim, H. W. Ko, I. Edery, Natural variation in the splice site strength of a clock gene and species-specific thermal adaptation. *Neuron* **60**, 1054–1067 (2008).

60. A. Kopp, O. Barmina, Evolutionary history of the *Drosophila* bipectinata species complex. *Genet. Res.* **85**, 23–46 (2005).

61. A. Seluzicki, M. Flourakis, E. Kula-Eversole, L. Zhang, V. Kilman, R. Allada, Dual PDF signaling pathways reset clocks via TIMELESS and acutely excite target neurons to control circadian behavior. *PLoS Biol.* **12**, e1001810 (2014).

62. V. Sabado, L. Vienne, J. M. Nunes, M. Rosbash, E. Nagoshi, Fluorescence circadian imaging reveals a PDF-dependent transcriptional regulation of the *Drosophila* molecular clock. *Sci. Rep.* **7**, 41560 (2017).

63. Y. Li, F. Guo, J. Shen, M. Rosbash, PDF and cAMP enhance PER stability in *Drosophila* clock neurons. *Proc. Natl. Acad. Sci. U.S.A.* **111**, E1284–E1290 (2014).

64. S. Martinek, S. Inonog, A. S. Manoukian, M. W. Young, A role for the segment polarity gene shaggy/GSK-3 in the *Drosophila* circadian clock. *Cell* **105**, 769–779 (2001).

65. D. Top, E. Harms, S. Syed, E. L. Adams, L. Saez, GSK-3 and CK2 kinases converge on timeless to regulate the master clock. *Cell Rep.* **16**, 357–367 (2016).

66. X. Fang, S. X. Yu, Y. Lu, R. C. J. Bast, J. R. Woodgett, G. B. Mills, Phosphorylation and inactivation of glycogen synthase kinase 3 by protein kinase A. *Proc. Natl. Acad. Sci. U.S.A.* **97**, 11960–11965 (2000).

67. M. Li, X. Wang, M. K. Meintzer, T. Laessig, M. J. Birnbaum, K. A. Heidenreich, Cyclic AMP promotes neuronal survival by phosphorylation of glycogen synthase kinase 3beta. *Mol. Cell. Biol.* **20**, 9356–9363 (2000).

68. D. Ma, D. Przybylski, K. C. Abruzzi, M. Schlichting, Q. Li, X. Long, M. Rosbash, A transcriptomic taxonomy of *Drosophila* circadian neurons around the clock. *eLife* **10**, e63056 (2021).

69. A. Lamaze, R. Stanewsky, DN1p or the "fluffy" cerberus of clock outputs. *Front. Physiol.* **10**, 1540 (2019).

70. A. Murad, M. Emery-Le, P. Emery, A subset of dorsal neurons modulates circadian behavior and light responses in *Drosophila*. *Neuron* **53**, 689–701 (2007).

71. M. Kunst, M. E. Hughes, D. Raccuglia, M. Felix, M. Li, G. Barnett, J. Duah, M. N. Nitabach, Calcitonin gene-related peptide neurons mediate sleep-specific circadian output in *Drosophila*. *Curr. Biol.* **24**, 2652–2664 (2014).

72. F. Guo, J. Yu, H. J. Jung, K. C. Abruzzi, W. Luo, L. C. Griffith, M. Rosbash, Circadian neuron feedback controls the *Drosophila* sleep-activity profile. *Nature* **536**, 292–297 (2016).

73. F. Guo, M. Holla, M. M. Díaz, M. Rosbash, A circadian output circuit controls sleep-wake arousal in *Drosophila*. *Neuron* **100**, 624–635.e4 (2018).

74. A. Lamaze, P. Krätschmer, K. F. Chen, S. Lowe, J. E. C. Jepson, A wake-promoting circadian output circuit in *Drosophila*. *Curr. Biol.* **28**, 3098–3105.e3 (2018).

75. F. Guo, X. Chen, M. Rosbash, Temporal calcium profiling of specific circadian neurons in freely moving flies. *Proc. Natl. Acad. Sci. U.S.A.* **114**, E8780–E8787 (2017).

76. R. A. Baines, J. P. Uhler, A. Thompson, S. T. Sweeney, M. Bate, Altered electrical properties in *Drosophila* neurons developing without synaptic transmission. *J. Neurosci.* **21**, 1523–1531 (2001).

77. F. N. Hamada, M. Rosenzweig, K. Kang, S. R. Pulver, A. Ghezzi, T. J. Jegla, P. A. Garrity, An internal thermal sensor controlling temperature preference in *Drosophila*. *Nature* **454**, 217–220 (2008).

78. A. Dawydow, R. Gueta, D. Ljaschenko, S. Ullrich, M. Hermann, N. Ehmann, S. Gao, A. Fiala, T. Langenhan, G. Nagel, R. J. Kittel, Channelrhodopsin-2-XXL, a powerful optogenetic tool for low-light applications. *Proc. Natl. Acad. Sci. U.S.A.* **111**, 13972–13977 (2014).

79. X. Liang, T. E. Holy, P. H. Taghert, A series of suppressive signals within the *Drosophila* circadian neural circuit generates sequential daily outputs. *Neuron* **94**, 1173–1189.e4 (2017).

80. M. Talay, E. B. Richman, N. J. Snell, G. G. Hartmann, J. D. Fisher, A. Sorkaç, J. F. Santoyo, C. Chou-Freed, N. Nair, M. Johnson, J. R. Szymanski, G. Barnea, Transsynaptic mapping of second-order taste neurons in flies by trans-Tango. *Neuron* **96**, 783–795.e4 (2017).

81. Y. Zhang, Y. Zhou, X. Zhang, L. Wang, Y. Zhong, Clock neurons gate memory extinction in *Drosophila*. *Curr. Biol.* **31**, 1337–1343.e4 (2021).

82. L. Bai, Y. Lee, C. T. Hsu, J. A. Williams, D. Cavanaugh, X. Zheng, C. Stein, P. Haynes, H. Wang, D. H. Gutmann, A. Sehgal, A conserved circadian function for the neurofibromatosis 1 gene. *Cell Rep.* **22**, 3416–3426 (2018).

83. D. J. Cavanaugh, J. D. Geratowski, J. R. Wooltorton, J. M. Spaethling, C. E. Hector, X. Zheng, E. C. Johnson, J. H. Eberwine, A. Sehgal, Identification of a circadian output circuit for rest:activity rhythms in *Drosophila*. *Cell* **157**, 689–701 (2014).

84. S. Park, J. Y. Sonn, Y. Oh, C. Lim, J. Choe, SIFamide and SIFamide receptor defines a novel neuropeptide signaling to promote sleep in *Drosophila*. *Mol. Cells* **37**, 295–301 (2014).

85. H. Huang, D. R. Possidente, C. G. Vecsey, Optogenetic activation of SIFamide (SIFa) neurons induces a complex sleep-promoting effect in the fruit fly *Drosophila melanogaster*. *Physiol. Behav.* **239**, 113507 (2021).

86. N. Reinhard, A. Fukuda, G. Manoli, E. Derksen, A. Saito, G. Möller, M. Sekiguchi, D. Rieger, C. Helfrich-Förster, T. Yoshii, M. Zandawala, Synaptic connectome of the *Drosophila* circadian clock. *Nat. Commun.* **15**, 10392 (2024).

87. L. K. Scheffer, C. S. Xu, M. Januszewski, Z. Lu, S. Y. Takemura, K. J. Hayworth, G. B. Huang, K. Shinomiya, J. Maitlin-Shepard, S. Berg, J. Clements, P. M. Hubbard, W. T. Katz, L. Umayam, T. Zhao, D. Ackerman, T. Blakely, J. Bogovic, T. Dolafi, D. Kainmueller, T. Kawase, K. A. Khairy, L. Leavitt, P. H. Li, L. Lindsey, N. Neubarth, D. J. Olbris, H. Otsuna, E. T. Trautman, M. Ito, A. S. Bates, J. Goldammer, T. Wolff, R. Svirskas, P. Schlegel, E. Neace, C. J. Knecht, C. X. Alvarado, D. A. Bailey, S. Ballinger, J. A. Borycz, B. S. Canino, N. Cheatham, M. Cook, M. Dreher, O. Duclos, B. Eubanks, K. Fairbanks, S. Finley, N. Forknall, A. Francis, G. P. Hopkins, E. M. Joyce, S. Kim, N. A. Kirk, J. Kovalyak, S. A. Lauchie, A. Lohff, C. Maldonado, E. A. Manley, S. McLin, C. Mooney, M. Ndama, O. Ogundeyi, N. Okeoma, C. Ordish, N. Padilla, C. M. Patrick, T. Paterson, E. E. Phillips, E. M. Phillips, N. Rampally, C. Ribeiro, M. K. Robertson, J. T. Rymer, S. M. Ryan, M. Sammons, A. K. Scott, A. L. Scott, A. Shinomiya, C. Smith, K. Smith, N. L. Smith, M. A. Sobeski, A. Suleiman, J. Swift, S. Takemura, I. Talebi, D. Tarnogorska, E. Tenshaw, T. Tokhi, J. J. Walsh, T. Yang, J. A. Horne, F. Li, R. Parekh, P. K. Rivlin, V. Jayaraman, M. Costa, G. S. Jefferis, K. Ito, S. Saalfeld, R. George, I. A. Meinertzhagen, G. M. Rubin, H. F. Hess, V. Jain, S. M. Plaza, A connectome and analysis of the adult *Drosophila* central brain. *eLife* **9**, e57443 (2020).

88. M. P. Shahandeh, L. Abuin, L. Lescuyer De Decker, J. Cergneux, R. Koch, E. Nagoshi, R. Benton, Circadian plasticity evolves through regulatory changes in a neuropeptide gene. *Nature* **635**, 951–959 (2024).

89. S. Bhutani, Natural entrainment of the Drosophila melanogaster circadian clock. PhD thesis. University of Leicester UK (2009).

90. J. H. Park, J. C. Hall, Isolation and chronobiological analysis of a neuropeptide pigment-dispersing factor gene in *Drosophila melanogaster*. *J. Biol. Rhythms* **13**, 219–228 (1998).

91. K. C. Abruzzi, A. Zadina, W. Luo, E. Wiyanto, R. Rahman, F. Guo, O. Shafer, M. Rosbash, RNA- seq analysis of *Drosophila* clock and non-clock neurons reveals neuron-specific cycling and novel candidate neuropeptides. *PLOS Genet.* **13**, e1006613 (2017).

92. A. A. Hoffmann, Physiological climatic limits in *Drosophila*: Patterns and implications. *J. Exp. Biol.* **213**, 870–880 (2010).

93. Y. Yang, I. Edery, Daywake, an anti-siesta gene linked to a splicing-based thermostat from an adjoining clock gene. *Curr. Biol.* **29**, 1728–1734.e4 (2019).

94. B. H. Collins, E. Rosato, C. P. Kyriacou, Seasonal behavior in *Drosophila melanogaster* requires the photoreceptors, the circadian clock, and phospholipase C. *Proc. Natl. Acad. Sci. U.S.A.* **101**, 1945–1950 (2004).

95. J. Majercak, W. F. Chen, I. Edery, Splicing of the period gene 3'-terminal intron is regulated by light, circadian clock factors, and phospholipase C. *Mol. Cell. Biol.* **24**, 3359–3372 (2004).

96. C. Breda, E. Rosato, C. P. Kyriacou, Norpa signalling and the seasonal circadian locomotor phenotype in *Drosophila*. *Biology* **9**, 130 (2020).

97. I. Edery, Did a small thermosensitive intron contribute to the temperate adaptation of *Drosophila melanogaster*? *Med. Res. Arch.* **11**, 4624 (2023).

98. F. Guo, I. Cerullo, X. Chen, M. Rosbash, PDF neuron firing phase-shifts key circadian activity neurons in *Drosophila*. *eLife* **3**, e02780 (2014).

99. S. Mezan, J. D. Feuz, B. Deplancke, S. Kadener, PDF signaling is an integral part of the *Drosophila* circadian molecular oscillator. *Cell Rep.* **17**, 708–719 (2016).

100. J. C. Chiu, H. W. Ko, I. Edery, NEMO/NLK phosphorylates PERIOD to initiate a time-delay phosphorylation circuit that sets circadian clock speed. *Cell* **145**, 357–370 (2011).

101. L. B. Duvall, P. H. Taghert, E and M circadian pacemaker neurons use different PDF receptor signalosome components in drosophila. *J. Biol. Rhythms* **28**, 239–248 (2013).

102. D. Top, M. W. Young, Coordination between differentially regulated circadian clocks generates rhythmic behavior. *Cold Spring Harb. Perspect. Biol.* **10**, a033589 (2018).

103. X. Zheng, A. Sehgal, AKT and TOR signaling set the pace of the circadian pacemaker. *Curr. Biol.* **20**, 1203–1208 (2010).

104. Q. Yuan, F. Lin, X. Zheng, A. Sehgal, Serotonin modulates circadian entrainment in *Drosophila*. *Neuron* **47**, 115–127 (2005).

105. E. Tauber, M. Zordan, F. Sandrelli, M. Pegoraro, N. Osterwalder, C. Breda, A. Daga, A. Selmin, K. Monger, C. Benna, E. Rosato, C. P. Kyriacou, R. Costa, Natural selection favors a newly derived timeless allele in *Drosophila melanogaster*. *Science* **316**, 1895–1898 (2007).

106. F. Sandrelli, E. Tauber, M. Pegoraro, G. Mazzotta, P. Cisotto, J. Landskron, R. Stanewsky, A. Piccin, E. Rosato, M. Zordan, R. Costa, C. P. Kyriacou, A molecular basis for natural selection at the timeless locus in *Drosophila melanogaster*. *Science* **316**, 1898–1900 (2007).

107. P. Deppisch, J. M. Prutscher, M. Pegoraro, E. Tauber, C. Wegener, C. Helfrich-Förster, Adaptation of *Drosophila melanogaster* to long photoperiods of high-latitude summers is facilitated by the ls-timeless allele. *J. Biol. Rhythms* **37**, 185–201 (2022).

108. A. Lamaze, C. Chen, S. Leleux, M. Xu, R. George, R. Stanewsky, A natural timeless polymorphism allowing circadian clock synchronization in “white nights”. *Nat. Commun.* **13**, 1724 (2022).

109. K. M. Vaze, G. Manoli, C. Helfrich-Förster, *Drosophila ezoana* uses morning and evening oscillators to adjust its rhythmic activity to different daylengths but only the morning oscillator to measure night length for photoperiodic responses. *J. Comp. Physiol. A Neuroethol. Sens. Neural Behav. Physiol.* **210**, 535–548 (2024).

110. C. E. Boothroyd, H. Wijnen, F. Naef, L. Saez, M. W. Young, Integration of light and temperature in the regulation of circadian gene expression in *Drosophila*. *PLOS Genet.* **3**, e54 (2007).

111. S. Montelli, G. Mazzotta, S. Vanin, L. Caccin, S. Corrà, C. De Pittà, C. Boothroyd, E. W. Green, C. P. Kyriacou, R. Costa, period and timeless mRNA splicing profiles under natural conditions in *Drosophila melanogaster*. *J. Biol. Rhythms* **30**, 217–227 (2015).

112. A. Martin Anduaga, N. Evantal, I. L. Patop, O. Bartok, R. Weiss, S. Kadener, Thermosensitive alternative splicing senses and mediates temperature adaptation in *Drosophila*. *eLife* **8**, e44642 (2019).

113. E. A. Nettnin, T. R. Sallese, A. Nasseri, S. Saurabh, D. J. Cavanaugh, Dorsal clock neurons in *Drosophila* sculpt locomotor outputs but are dispensable for circadian activity rhythms. *iScience* **24**, 103001 (2021).

114. Q. Liu, M. Tabuchi, S. Liu, L. Kodama, W. Horiuchi, J. Daniels, L. Chiu, D. Baldoni, M. N. Wu, Branch-specific plasticity of a bifunctional dopamine circuit encodes protein hunger. *Science* **356**, 534–539 (2017).

115. M. P. Fernández, J. Berni, M. F. Ceriani, Circadian remodeling of neuronal circuits involved in rhythmic behavior. *PLOS Biol.* **6**, e69 (2008).

116. J. M. Duhart, A. Herrero, G. de la Cruz, J. I. Ispizua, N. Pírez, M. F. Ceriani, Circadian structural plasticity drives remodeling of E cell output. *Curr. Biol.* **30**, 5040–5048.e5 (2020).

117. E. A. Gorostiza, A. Depetris-Chauvin, L. Frenkel, N. Pirez, M. F. Ceriani, Circadian pacemaker neurons change synaptic contacts across the day. *Curr. Biol.* **24**, 2161–2167 (2014).

118. M. P. Fernandez, H. L. Pettibone, J. T. Bogart, C. J. Roell, C. E. Davey, A. Pranevicius, K. V. Huynh, S. M. Lennox, B. S. Kostadinov, O. T. Shafer, Sites of circadian clock neuron plasticity mediate sensory integration and entrainment. *Curr. Biol.* **30**, 2225–2237.e5 (2020).

119. A. Herrero, T. Yoshii, J. I. Ispizua, C. Colque, J. A. Veenstra, N. I. Muraro, M. F. Ceriani, Coupling neuropeptide levels to structural plasticity in *Drosophila* clock neurons. *Curr. Biol.* **30**, 3154–3166.e4 (2020).

120. M. P. Nusbaum, D. M. Blitz, E. Marder, Functional consequences of neuropeptide and small-molecule co-transmission. *Nat. Rev. Neurosci.* **18**, 389–403 (2017).

121. V. Svensson, R. Vento-Tormo, S. A. Teichmann, Exponential scaling of single-cell RNA-seq in the past decade. *Nat. Protoc.* **13**, 599–604 (2018).

122. L. Zhang, B. Y. Chung, B. C. Lear, V. L. Kilman, Y. Liu, G. Mahesh, R. A. Meissner, P. E. Hardin, R. Allada, $DN1_p$ circadian neurons coordinate acute light and PDF inputs to produce robust daily behavior in *Drosophila*. *Curr. Biol.* **20**, 591–599 (2010).

123. S. Yadlapalli, C. Jiang, A. Bahle, P. Reddy, E. Meyhofer, O. T. Shafer, Circadian clock neurons constantly monitor environmental temperature to set sleep timing. *Nature* **555**, 98–102 (2018).

124. S. Terhzaz, P. Rosay, S. F. Goodwin, J. A. Veenstra, The neuropeptide SIFamide modulates sexual behavior in *Drosophila*. *Biochem. Biophys. Res. Commun.* **352**, 305–310 (2007).

125. C. Martelli, U. Pech, S. Kobbenbring, D. Pauls, B. Bahl, M. V. Sommer, A. Pooryasin, J. Barth, C. W. P. Arias, C. Vassiliou, A. J. F. Luna, H. Poppinga, F. G. Richter, C. Wegener, A. Fiala, T. Riemensperger, SIFamide translates hunger signals into appetitive and feeding behavior in *Drosophila*. *Cell Rep.* **20**, 464–478 (2017).

126. A. P. Dreyer, M. M. Martin, C. V. Fulgham, D. A. Jabr, L. Bai, J. Beshel, D. J. Cavanaugh, A circadian output center controlling feeding:fasting rhythms in *Drosophila*. *PLOS Genet.* **15**, e1008478 (2019).

127. B. J. Song, S. J. Sharp, D. Rogulja, Daily rewiring of a neural circuit generates a predictive model of environmental light. *Sci. Adv.* **7**, eabe4284 (2021).

128. V. Hartenstein, The neuroendocrine system of invertebrates: A developmental and evolutionary perspective. *J. Endocrinol.* **190**, 555–570 (2006).

129. Y. H. Belgacem, J. R. Martin, Neuroendocrine control of a sexually dimorphic behavior by a few neurons of the pars intercerebralis in *Drosophila*. *Proc. Natl. Acad. Sci. U.S.A.* **99**, 15154–15158 (2002).

130. K. Foltenyi, R. J. Greenspan, J. W. Newport, Activation of EGFR and ERK by rhomboid signaling regulates the consolidation and maintenance of sleep in *Drosophila*. *Nat. Neurosci.* **10**, 1160–1167 (2007).

131. J. B. Henningsen, F. Gauer, V. Simonneaux, RFRP neurons - The doorway to understanding seasonal reproduction in mammals. *Front. Endocrinol.* **7**, 36 (2016).

132. M. Brankatschk, T. Gutmann, O. Knittelfelder, A. Palladini, E. Prince, M. Grzybek, B. Brankatschk, A. Shevchenko, Ü. Coskun, S. Eaton, A temperature-dependent switch in feeding preference improves *Drosophila* development and survival in the cold. *Dev. Cell* **46**, 781–793.e4 (2018).

133. W. S. Lin, S. R. Yeh, S. Z. Fan, L. Y. Chen, J. H. Yen, T. F. Fu, M. S. Wu, P. Y. Wang, Insulin signaling in female *Drosophila* links diet and sexual attractiveness. *FASEB J.* **32**, 3870–3877 (2018).

134. A. C. Keene, E. R. Duboue, D. M. McDonald, M. Dus, G. S. Suh, S. Waddell, J. Blau, Clock and cycle Limit Starvation-Induced Sleep Loss in Drosophila. *Curr. Biol.* **20**, 1209–1215 (2010).

135. M. Cavey, B. Collins, C. Bertet, J. Blau, Circadian rhythms in neuronal activity propagate through output circuits. *Nat. Neurosci.* **19**, 587–595 (2016).

136. K. J. Venken, K. L. Schulze, N. A. Haelterman, H. Pan, Y. He, M. Evans-Holm, J. W. Carlson, R. W. Levis, A. C. Spradling, R. A. Hoskins, H. J. Bellen, MiMIC: A highly versatile transposon insertion resource for engineering *Drosophila melanogaster* genes. *Nat. Methods* **8**, 737–743 (2011).

137. M. Z. Li, S. J. Elledge, Harnessing homologous recombination in vitro to generate recombinant DNA via SLIC. *Nat. Methods* **4**, 251–256 (2007).

138. S. Mansourian, A. Enjin, E. V. Jirle, V. Ramesh, G. Rehermann, P. G. Becher, J. E. Pool, M. C. Stensmyr, Wild African *Drosophila melanogaster* are seasonal specialists on Marula fruit. *Curr. Biol.* **28**, 3960–3968.e3 (2018).

139. L. Soto-Yéber, J. Soto-Ortiz, P. Godoy, R. Godoy-Herrera, The behavior of adult *Drosophila* in the wild. *PLOS ONE* **13**, e0209917 (2018).

140. M. S. Ross, L. B. Flanagan, G. H. L. Roi, Seasonal and successional changes in light quality and quantity in the understory of boreal forest ecosystems. *Can. J. Bot.* **24**, 2792–2799 (1986).

141. M. H. Turnbull, D. J. Yates, Seasonal variation in the red/far-red ratio and photon flux density in an Australian sub-tropical rainforest. *Agric. For. Meteorol.* **64**, 111–127 (1993).

142. H. R. Nuñez, R. C. de Gouvenain, Seasonal variation in understory light near a gap edge and its association with conifer seedling survival in a southern New England forest. *Northeast Nat.* **22**, 613–629 (2015).

143. S. M. Hartikainen, M. Pieristè, J. Lassila, T. M. Robson, Seasonal patterns in spectral irradiance and leaf UV-A absorbance under forest canopies. *Front. Plant Sci.* **10**, 1762 (2019).

144. A. Deckard, R. C. Anafi, J. B. Hogenesch, S. B. Haase, J. Harer, Design and analysis of large-scale biological rhythm studies: A comparison of algorithms for detecting periodic signals in biological data. *Bioinformatics* **29**, 3174–3180 (2013).

145. O. M. Chaves, G. Avalos, Do seasonal changes in light availability influence the inverse leafing phenology of the neotropical dry forest understory shrub *Bonellia nervosa* (*Theophrastaceae*). *Rev. Biol. Trop.* **56**, 257–268 (2008).

146. S. Tripathi, R. Bhadouria, P. Srivastava, R. S. Devi, R. Chaturvedi, A. S. Raghubanshi, Effects of light availability on leaf attributes and seedling growth of four tree species in tropical dry forest. *Ecol. Process* **9**, 2 (2020).

147. C. Choi, G. Cao, A. K. Tanenhaus, E. V. McCarthy, M. Jung, W. Schleyer, Y. Shang, M. Rosbash, J. C. Yin, M. N. Nitabach, Autoreceptor control of peptide/neurotransmitter corelease from PDF neurons determines allocation of circadian activity in *Drosophila*. *Cell Rep.* **2**, 332–344 (2012).

148. M. Damulewicz, G. M. Mazzotta, E. Sartori, E. Rosato, R. Costa, E. M. Pyza, Cryptochrome is a regulator of synaptic plasticity in the visual system of *Drosophila melanogaster*. *Front. Mol. Neurosci.* **10**, 1660 (2017).

149. J. Bischof, E. M. Sheils, M. Bjorklund, K. Basler, Generation of a transgenic ORFeome library in *Drosophila*. *Nat. Protoc.* **9**, 1607–1620 (2014).

150. B. Hudry, S. Viala, Y. Graba, S. Merabet, Visualization of protein interactions in living *Drosophila* embryos by the bimolecular fluorescence complementation assay. *BMC Biol.* **9**, 5 (2011).

151. F. Alejevski, A. Saint-Charles, C. Michard-Vanhée, B. Martin, S. Galant, D. Vasiliauskas, F. Rouyer, The HisCl1 histamine receptor acts in photoreceptors to synchronize *Drosophila* behavioral rhythms with light-dark cycles. *Nat. Commun.* **10**, 252 (2019).

152. A. Klarsfeld, S. Malpel, C. Michard-Vanhée, M. Picot, E. Chélot, F. Rouyer, Novel features of cryptochrome-mediated photoreception in the brain circadian clock of *Drosophila*. *J. Neurosci.* **24**, 1468–1477 (2004).

153. J. O. Gummadova, G. A. Coutts, N. R. Glossop, Analysis of the *Drosophila* clock promoter reveals heterogeneity in expression between subgroups of central oscillator cells and identifies a novel enhancer region. *J. Biol. Rhythms* **24**, 353–367 (2009).

154. M. T. Li, L. H. Cao, N. Xiao, M. Tang, B. Deng, T. Yang, T. Yoshii, D. G. Luo, Hub-organized parallel circuits of central circadian pacemaker neurons for visual photoentrainment in *Drosophila*. *Nat. Commun.* **9**, 4247 (2018).

155. J. H. Bahn, G. Lee, J. H. Park, Comparative analysis of Pdf-mediated circadian behaviors between Drosophila melanogaster and D. virilis. *Genetics* **181**, 965–975 (2009).

156. W. Li, J. T. Ohlmeyer, M. E. Lane, D. Kalderon, Function of protein kinase A in hedgehog signal transduction and *Drosophila* imaginal disc development. *Cell* **80**, 553–562 (1995).

157. E. Blanchardon, B. Grima, A. Klarsfeld, E. Chélot, P. E. Hardin, T. Préat, F. Rouyer, Defining the role of *Drosophila* lateral neurons in the control of circadian rhythms in motor

activity and eclosion by targeted genetic ablation and PERIOD protein overexpression. *Eur. J. Neurosci.* **13**, 871–888 (2001).

158. T. Copf, Developmental shaping of dendritic arbors in *Drosophila* relies on tightly regulated intra-neuronal activity of protein kinase A (PKA). *Dev. Biol.* **393**, 282–297 (2014).

159. H. Kanuka, E. Kuranaga, K. Takemoto, T. Hiratou, H. Okano, M. Miura, Drosophila caspase transduces Shaggy/GSK-3beta kinase activity in neural precursor development. *EMBO J.* **24**, 3793–3806 (2005).

160. S. Tanoue, P. Krishnan, B. Krishnan, S. E. Dryer, P. E. Hardin, Circadian clocks in antennal neurons are necessary and sufficient for olfaction rhythms in *Drosophila*. *Curr. Biol.* **14**, 638–649 (2004).

161. M. J. Muskus, F. Preuss, J. Y. Fan, E. S. Bjes, J. L. Price, Drosophila DBT lacking protein kinase activity produces long-period and arrhythmic circadian behavioral and molecular rhythms. *Mol. Cell. Biol.* **27**, 8049–8064 (2007).

162. S. Hyun, Y. Lee, S. T. Hong, S. Bang, D. Paik, J. Kang, J. Shin, J. Lee, K. Jeon, S. Hwang, E. Bae, J. Kim, Drosophila GPCR Han is a receptor for the circadian clock neuropeptide PDF. *Neuron* **48**, 267–278 (2005).

163. M. D. Gordon, K. Scott, Motor control in a *Drosophila* taste circuit. *Neuron* **61**, 373–384 (2009).

164. K. Feng, M. T. Palfreyman, M. Hasemeyer, A. Talsma, B. J. Dickson, Ascending SAG neurons control sexual receptivity of *Drosophila* females. *Neuron* **83**, 135–148 (2014).
